# Six tris­(bipyrid­yl)iron(II) complexes with 2-substituted 1,1,3,3-tetra­cyano­propenide, perchlorate and tetra­fluorido­borate anions; order *versus* disorder, hydrogen bonding and C—N⋯π inter­actions

**DOI:** 10.1107/S2056989018015426

**Published:** 2018-11-06

**Authors:** Abderezak Addala, Zouaoui Setifi, Yukio Morimoto, Beñat Artetxe, Takashi Matsumoto, Juan M. Gutiérrez-Zorrilla, Christopher Glidewell

**Affiliations:** a Laboratoire de Chimie, Ingénierie Moléculaire et Nanostructures (LCIMN), Université Ferhat Abbas Sétif 1, Sétif 19000, Algeria; b Département de Technologie, Faculté de Technologie, Université 20 Août 1955-Skikda, BP 26, Route d’El-Hadaiek, Skikda 21000, Algeria; c Division of Quantum Beam Material Science, Research Reactor Institute, Kyoto University, Kumatori, Osaka 590-0494, Japan; d Departamento de Química Inorgánica, Facultad de Ciencia y Tecnología, Universidad de País Vasco UPV/EHU, PO Box 644, E-48080 Bilbao, Spain; e Application Laboratories, Rigaku Corporation, 3-9-12, Matsubara-cho, Akishima, Tokyo 196-8666, Japan; f School of Chemistry, University of St Andrews, St Andrews, Fife KY16 9ST, UK

**Keywords:** synthesis, tris­(bipyrid­yl)iron(II) complexes, polynitrile anions, crystal structure, disorder, hydrogen bonding, C—N⋯π inter­actions, supra­molecular assembly

## Abstract

Structures are reported for six closely related salts of tris­(bipyrid­yl)iron(II) cations, namely tris­(2,2′-bi­pyridine)­iron(II) bis­(1,1,3,3-tetra­cyano-2-meth­oxy­propenide) 0.776-hydrate, [Fe(C_10_H_8_N_2_)_3_](C_8_H_3_N_4_O)_2_.0.776H_2_O, (I), tris­(2,2′-bi­pyridine)­iron(II) 1,1,3,3-tetra­cyano-2-(propyl­sulfan­yl)propenide perchlor­ate, [Fe(C_10_H_8_N_2_)_3_](C_10_H_7_N_4_S)(ClO_4_), (II), tris­(5,5′-dimethyl-2,2′-bi­pyridine)­iron(II) 1,1,3,3-tetra­cyano-2-meth­oxy­propenide tetra­fluorido­borate ethanol 0.926-solvate, [Fe(C_12_H_12_N_2_)_3_](C_8_H_3_N_4_O)(BF_4_).0.926C_2_H_2_O, (III), tris­(5,5′-dimethyl-2,2′-bi­pyridine)­iron(II) 1,1,3,3-tetra­cyano-2-eth­oxy­propenide tetra­fluorido­borate, [Fe(C_12_H_12_N_2_)_3_](C_9_H_5_N_4_O)(BF_4_), (IV), tris­(5,5′-dimethyl-2,2′-bi­pyridine)­iron(II) 1,1,3,3-tetra­cyano-2-(ethyl­sufanyl)propenide tetra­fluorido­borate, [Fe(C_12_H_12_N_2_)_3_](C_9_H_5_N_4_S)(BF_4_), (V), and tris­(5,5′-dimethyl-2,2′-bi­pyri­dine)­iron(II) 1,1,3,3-tetra­cyano-2-prop­oxypropenide tetra­fluorido­borate, [Fe(C_12_H_12_N_2_)_3_](C_10_H_7_N_4_O)(BF_4_), (VI). In compound (I), one of the anions is disordered over two sets of atomic sites with equal occupancies while, in the second anion, just one of the C(CN)_2_ units is disordered, again over two sets of atomic sites with equal occupancies: the anionic components are linked by multiple C—H⋯N hydrogen bonds to form a three-dimensional framework. In compound (II), the polynitrile anion is disordered over two sets of atomic sites with occupancies in the approximate ratio 3:1, while the perchlorate anion is disordered over three sets of atomic sites: there are C—N⋯π inter­actions between the cations and the polynitrile anion. The polynitrile anion in compound (III) is fully ordered, but the tetra­fluorido­borate anion is disordered over two sets of atomic sites with occupancies 0.671 (4) and 0.329 (4): the cations and the tetra­fluorido­borate anions are linked by C—H⋯F inter­actions to form an inter­rupted chain. Compounds (IV) and (V) are isostructural and all of the ionic components are fully ordered in both of them: the cations and tetra­fluorido­borate anions are linked into *C*_2_^2^(12) chains. The polynitrile anion in compound (VI) is disordered over two sets of atomic sites with approximately equal occupancies, and here the chains formed by the cations and the tetra­fluorido­borate anions are of the *C*_2_^2^(13) type.

## Abstract 2

The structures are reported of six racemic tris­(bipyrid­yl)iron salts with a range of 2-substituted-1,1,3,3-tetra­cyano­propenide anions, mostly also containing either perchlorate or tetra­fluorido­borate as co-anions. In three of the compounds the polynitrile anions are fully ordered, and in three others they are disordered, while the co-anion is also ordered in three compounds, but disordered in two others. Supra­molecular assemblies range from no continuous aggregation up to a three-dimensional hydrogen-bonded framework structure.

## Chemical context   

The use of polynitrile anions as ligands, either alone or in combination with neutral co-ligands, is a very versatile and effective strategy for developing mol­ecular architectures with different topologies and dimensionalities, as a result of their ability to coordinate and bridge metal ions in many different ways (Benmansour *et al.*, 2008 ▸, 2010 ▸, 2012 ▸; Atmani *et al.*, 2008 ▸; Gaamoune *et al.*, 2010 ▸; Setifi, Setifi, El Ammari *et al.*, 2014 ▸; Addala *et al.*, 2015 ▸). The presence of other potential donor groups such as –OH, –SH or –NH_2_, together with their rigidity and their electronic delocalization, can lead to the synthesis of new magnetic and luminescent coordination polymers with transition-metal ions (Benmansour *et al.*, 2010 ▸; Yuste *et al.*, 2009 ▸; Setifi *et al.*, 2009 ▸; Setifi, Zambon *et al.*, 2017 ▸; Kayukov *et al.*, 2017 ▸; Lehchili *et al.*, 2017 ▸). Furthermore, these ligands have shown both coordinating and bridging capabilities in novel discrete and polymeric bi-stable materials (Setifi, Milin *et al.*, 2014 ▸; Milin *et al.*, 2016 ▸; Pittala *et al.*, 2017 ▸).
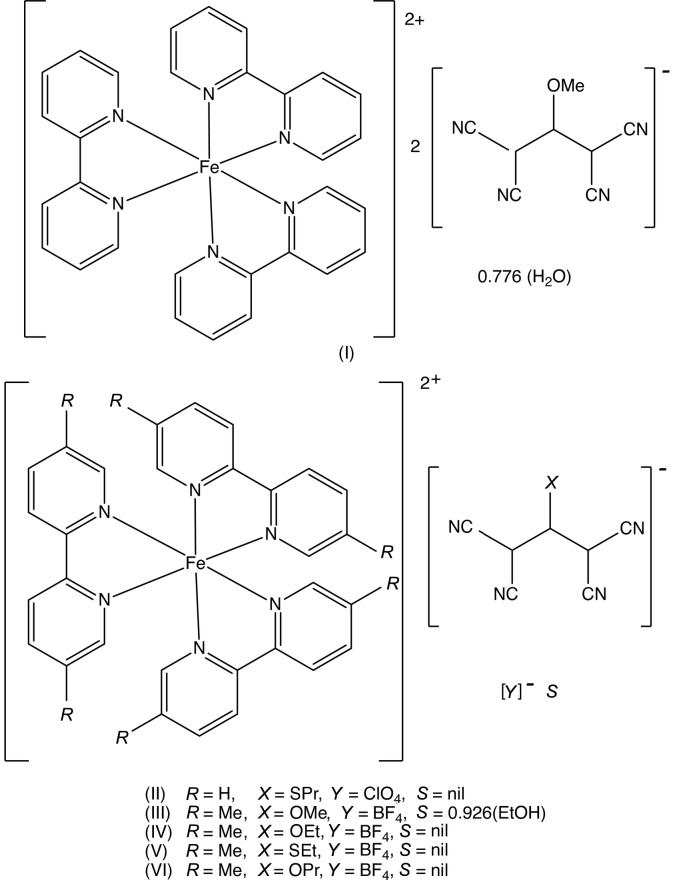


**Figure 1:**
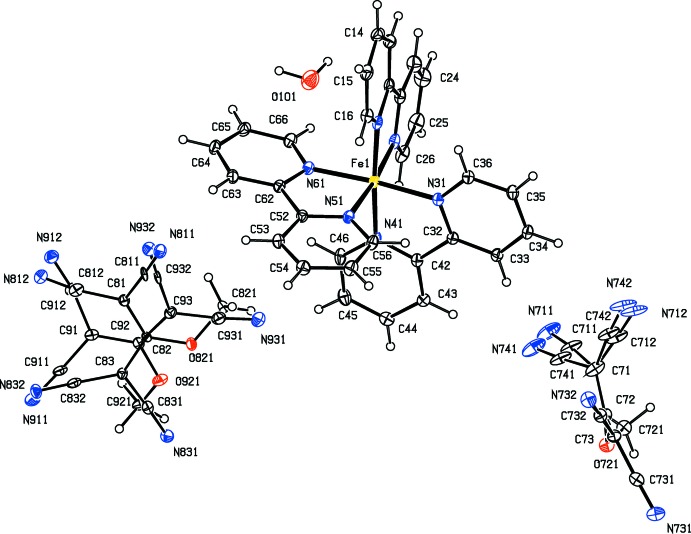
The independent ionic components in compound (I), showing the atom-labelling scheme. Displacement ellipsoids are drawn at the 30% probability level.

As a part of our continuing study of the structural and magnetic properties of iron(II) complexes containing both polynitrile and polypyridyl units (Setifi *et al.*, 2010 ▸; Setifi, Domasevitch *et al.*, 2013 ▸; Setifi, Setifi *et al.*, 2013 ▸; Setifi, Setifi, Boughzala *et al.*, 2014 ▸; Setifi, Setifi, El Ammari *et al.*, 2014 ▸), we report here the mol­ecular and supra­molecular structures of six tris­(bipyrid­yl)iron(II) compounds each containing a 2-substituted-1,1,3,3-tetra­cyano­propenide anion as counter-ion, namely tris­(2,2′-bi­pyridine)­iron(II), bis­(1,1,3,3-tetra­cyano-2-meth­oxy­propenide) 0.776(hydrate) (I), tris­(2,2′-bi­pyridine)­iron(II) 1,1,3,3-tetra­cyano-2-(propyl­sulfan­yl)propenide perchlorate (II), tris­(5,5′-dimethyl-2,2′-bi­pyridine)­iron(II) 1,1,3,3-tetra­cyano-2-meth­oxy­propenide tetra­fluorido­borate 0.926-ethanol solvate (III), tris­(5,5′-dimethyl-2,2′-bi­pyridine)­iron(II) 1,1,3,3- tetra­cyano-2-eth­oxy­propenide tetra­fluorido­borate (IV), tris­(5,5′-dimethyl-2,2′-bi­pyridine)­iron(II) 1,1,3,3- tetra­cyano-2-(ethyl­sufanyl)propenide tetra­fluorido­borate (V), and tris­(5,5′-dimethyl-2,2′-bi­pyridine)­iron(II) 1,1,3,3-tetra­cyano-2-prop­oxypropenide tetra­fluorido­borate (VI) (Figs. 1 ▸–6 ▸ ▸ ▸ ▸ ▸).

The polynitrile anions all have the constitution 1,1,3,3-tetra­cyano-2-*X*-propenide (tcn*X*), and it will be convenient to use abbreviations as follows: *X* = OMe, tcnome; *X* = OEt, tcnoet; *X* = OPr, tcnopr; *X* = SEt, tcnset; *X* = SPr, tcnspr (*cf* Scheme). The compounds were all prepared using solvothermal reactions between mixtures of iron(II) salts, a 2,2′-bi­pyridine and polynitrile salts of the type K(tcn*X*), where the substituent *X* is as defined above.

## Structural commentary   

Compounds (I)–(VI) all contain a tris­(bi­pyridine)­iron(II) cation and a 2-substituted-1,1,3,3-tetra­cyano­propenide anion. In compounds (I) and (II), the ligand is the unsubstituted 2,2′-bi­pyridine, and in compounds (III)–(VI), it is 5,5′-di­meth­yl-2,2′-bi­pyridine. In compound (I) there are two propenide anions, along with a water mol­ecule having occupancy 0.776 (6); in compound (II), there is a single propenide anion and a perchlorate ion, while in each of (III)–(VI) there is a single propenide anion and a tetra­fluorido­borate ion. All of the compounds crystallize in centrosymmetric space groups (Table 3 ▸), so that they contain equal numbers of cations having the Δ and Λ configurations: in each case the reference cation was selected to be the one having the Δ configuration.

In several of the compounds, the anions exhibit disorder. One of the propenide anions in compound (I), that containing atom O721 (Fig. 1 ▸) exhibits disorder of one of the C(CN)_2_ units over two orientations with occupancies which refined to values which are equal within experimental uncertainly, 0.501 (7) and 0.499 (7), while the other anion, containing atom O821, exhibits whole anion disorder, again over two sets of atomic sites with refined occupancies 0.502 (2) and 0.498 (2): all of these occupancies were therefore set to 0.5. In compound (II), the propenide anion exhibits whole anion disorder over two sets of atomic sites with occupancies 0.754 (2) and 0.246 (2), while the disorder of the perchlorate anion was modelled using three sets of sites having occupancies 0.439 (3), 0.377 (3) and 0.184 (3).

The propenide anion of compound (III) is fully ordered, but the tetra­fluorido­borate anion is disordered over two sets of atomic sites with occupancies 0.671 (4) and 0.329 (4): there is also an ethanol mol­ecule present in the structure of (III) with occupancy 0.926 (5). There is no detectable disorder in the isostructural compounds (IV) and (V), but in compound (VI) the propenide anion is disordered over two sets of atomic sites with occupancies 0.508 (6) and 0.492 (6).

In none of compounds (I)–(VI) do the polynitrile units act as ligands towards the iron(II) centres, but they are always present as free anions. This is consistent with the behaviour observed in a wide range of other iron(II) complexes containing polypyridyl ligands as anions of the general type tcn*X* (Setifi *et al.*, 2010 ▸; Setifi, Domasevitch *et al.*, 2013 ▸; Setifi, Setifi *et al.*, 2013 ▸; Setifi, Setifi, Boughzala *et al.*, 2014 ▸). Likewise, free tcnoet anions are present in meso-di-μ-chlorido-bis­(2,2′-bi­pyridine)­cadmium bis­(1,1,3,3-tetra­cyano-2-eth­oxy­propenide 0.81-hydrate (Setifi, Morgenstern *et al.*, 2017 ▸). On the other hand, tcnoet has been found to act as a monodentate ligand in both mononuclear (Setifi, Setifi, El Ammari *et al.*, 2014 ▸) and dinuclear (Addala *et al.*, 2015 ▸) copper(II) complexes. By contrast, the simpler anion dicyanamide [N(CN)_2_]^−^, containing just two cyano groups as opposed to the four cyano groups in anions of type (tcn*X*)^−^, readily acts as a ligand towards iron(II) (Setifi, Konieczny *et al.*, 2017 ▸; Setifi, Geiger *et al.*, 2018 ▸).

It is inter­esting to note that the polynitrile anions in compounds (II)–(V) are fully ordered while those in compounds (I), (II) and (VI) are disordered, and it is tempting to look to the direction-specific inter­ionic inter­actions involv­ing these ions for clues to the differences in behaviour. However, in (III)– (V) each of the ordered polynitrile anions only participates in a single hydrogen bond (Table 1 ▸), as is the case also for the disordered anion in (VI), whereas in both (I) and (II) the polynitrile anion participates in a large number of hydrogen bonds: in (I), also one of the C(CN_2_) units in each orientation is involved, but in (II) both C(CN_2_) units in both orientations are involved in hydrogen bonds, thus tethering these anions at both ends. Hence, no plausible explanation of polynitrile order versus disorder can be gleaned from hydrogen bonding: nor do the C—N⋯π contacts provide any explanation, as there are more of these in (II) than in (III), while such short contacts are absent from the structures of (I) and (IV)–(VI).

The Fe—N distances in compounds (I)–(VI) all lie within a narrow range of less than 0.03 Å, with extreme values of 1.9579 (12) Å in (V) and 1.985 (3) Å in (III). These values indicate, in each compound, the presence of low-spin Fe^II^; in comparable high-spin complexes, the Fe—N distances are always around 2.15 Å (Orpen *et al.*, 1989 ▸).

## Supra­molecular features   

With the exception of the isostructural pair of compounds (IV) and (V), the analysis of the supra­molecular assembly is generally complicated by the various forms of anion disorder.

The supra­molecular aggregation in compounds (I)–(VI) depends upon hydrogen bonds of a number of different types (Table 1 ▸); nearly all of the hydrogen bonds involve a donor from the cation and an acceptor from one of the anions, and so these may be regarded as charge-assisted hydrogen bonds (Gilli *et al.*, 1994 ▸). The links between the cations and the polynitrile anions are based on C—H⋯N hydrogen bonds, augmented in compounds (II) and (III) by C—N⋯π inter­actions (Table 2 ▸). C—H⋯O hydrogen bonds are present in the perchlorate salt (II) and C—H⋯F hydrogen bonds in the salts (III)–(VI). In addition, the partial hydrate (I) contains a C—H⋯O hydrogen bond together with O—H⋯N hydrogen bonds involving just one of the two independent polynitrile anions; by contrast the partial ethanol solvate (III) contains just one O—H⋯N hydrogen bond linking the ethanol component to the ordered polynitrile anion.

**Figure 7:**
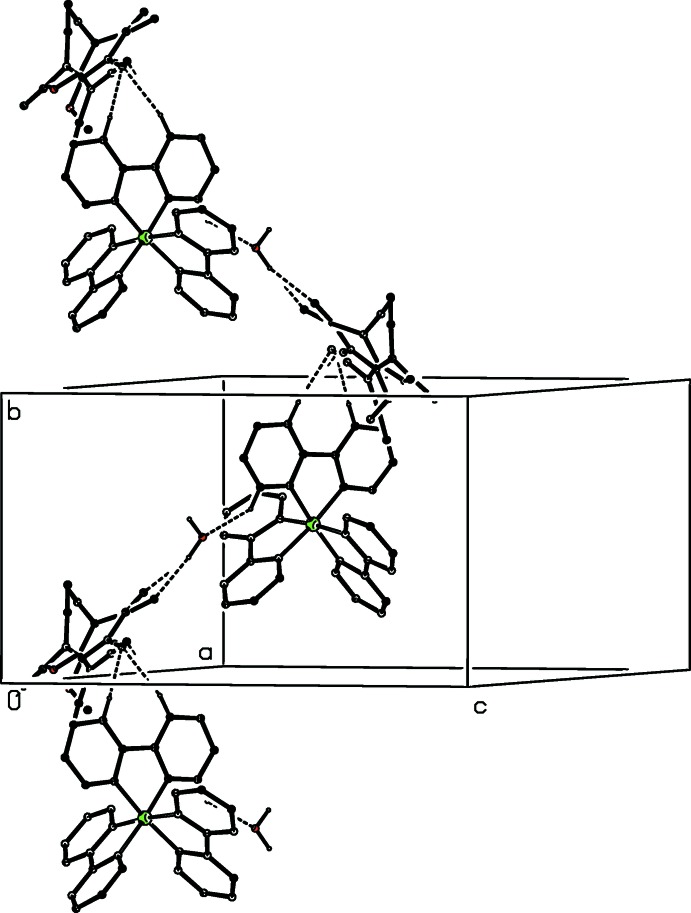
Part of the crystal structure of compound (I) showing the formation of a hydrogen-bonded chain running parallel to the [010] direction. For the sake of clarity, the type 1 anion and the H atoms not involved in the motif shown have been omitted.

**Figure 8:**
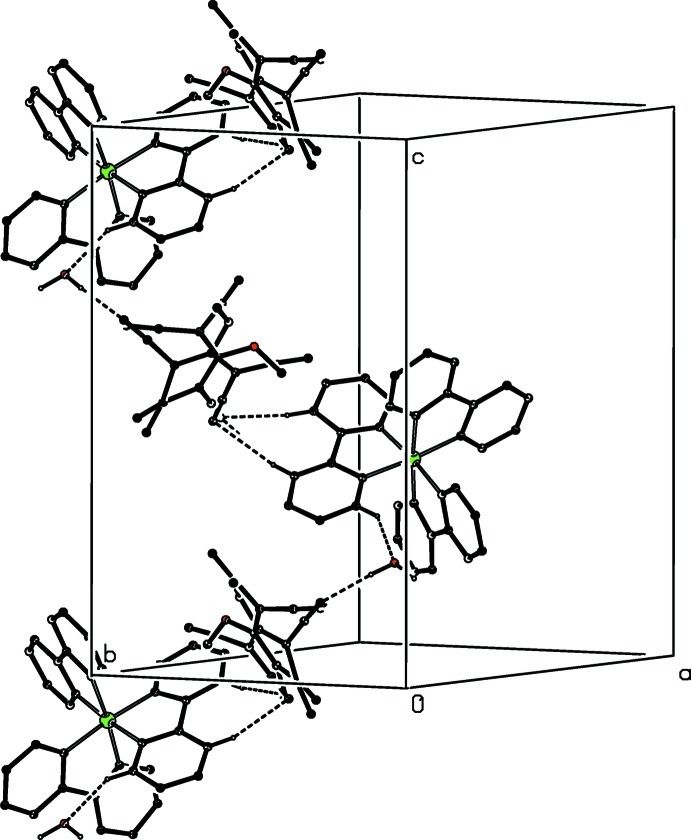
Part of the crystal structure of compound (I) showing the formation of a hydrogen-bonded chain running parallel to the [001] direction. For the sake of clarity, the type 1 anion and the H atoms not involved in the motif shown have been omitted.

**Figure 9:**
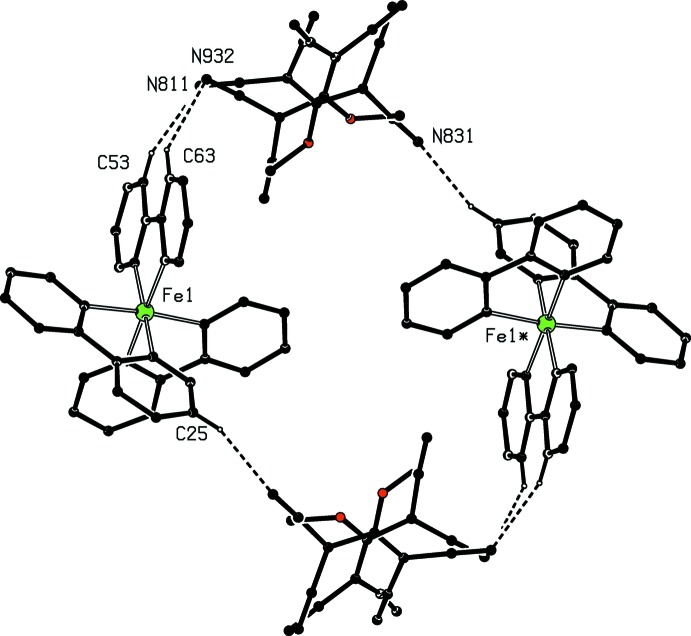
Part of the crystal structure of compound (I) showing the formation of the hydrogen-bonded ring motif, which links the (100) sheets. For the sake of clarity, the type 1 anion and the water mol­ecule, the H atoms not involved in the motif shown, and the unit-cell outline have all been omitted. The Fe atom marked with an asterisk (*) is at the symmetry position (1 − *x*, 1 − *y*, 1 − *z*).

In compound (I), the independent components are linked by a substantial number of hydrogen bonds, six of which lie within the selected asymmetric unit (Fig. 1 ▸, Table 1 ▸), to form a three-dimensional framework structure, whose formation can be readily analysed in terms of three simpler sub-structures (Ferguson *et al.*, 1998*a* ▸,*b* ▸; Gregson *et al.*, 2000 ▸): it will be convenient to refer to the anions containing atoms O721 and O821 as anions 1 and 2 respectively. Aggregates consisting of the cation, anion 2 and the water component, which are related by the 2_1_ screw axis along (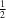, *y*, 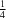) are linked to form a complex chain running parallel to the [010] direction (Fig. 7 ▸), while similar aggregates which are related by the *c*-glide plane at *y* = 1 form a second, equally complex chain running parallel to the [001] direction (Fig. 8 ▸). The combination of these two chain motifs gives rise to a sheet structure lying parallel to (100) and adjacent sheets are linked by a centrosymmetric motif involving only the cations and the type 2 anions (Fig. 9 ▸). Despite the disorder, the cooperative action of the hydrogen bonds leads to a coherent three-dimensional structure.

In compound (II), the occupancies of the tcnspr anion, 0.754 (2) and 0.246 (2), mean that inter­actions involving only the minor component can probably be ignored from the point of view of the supra­molecular aggregation; in any event, of the C—H⋯N contacts, only that within the selected asymmetric unit has a *D*—H⋯*A* angle greater than 140°, so that the others can probably be discounted as structurally unimportant (Wood *et al.*, 2009 ▸). All of the disorder components of the perchlorate anion have occupancies significantly less than 0.5, and the inter­actions involving these do not lead to any continuous aggregation.

**Figure 10:**
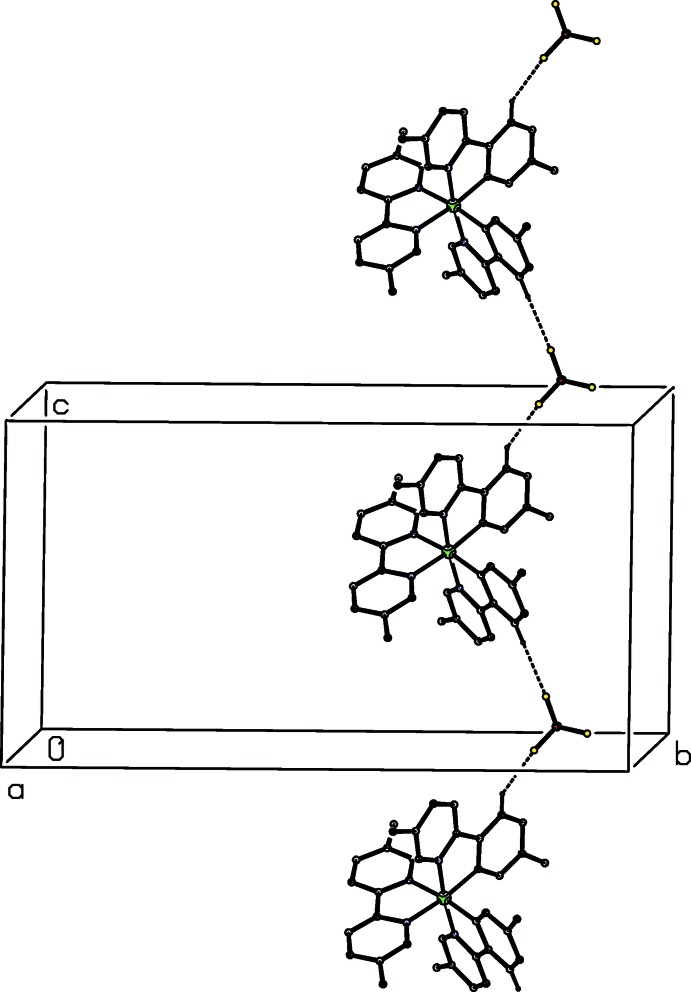
Part of the crystal structure of compound (III) showing the formation of a hydrogen-bonded 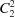
(12) chain running parallel to the [001] direction. For the sake of clarity, the tcnome anion, the ethanol component and the H atoms not involved in the motif shown have been omitted.

The partial-occupancy ethanol component in compound (III) is linked to the tcnome anion by an O—H⋯N hydrogen bond, but these two components play no further role in the supra­molecular assembly: it seems likely that the ethanol component is present primarily in a space-filling role. The cation and the major disorder component of the tetra­fluorido­borate anion are linked by a C–H⋯F hydrogen bond within the selected asymmetric unit and bimolecular aggregates of this type which are related by translation are linked to form a 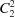
(12) (Bernstein *et al.*, 1995 ▸) chain running parallel to the [001] direction (Fig. 10 ▸): this will be an inter­rupted chain because of the disorder exhibited by the tetra­fluorido­borate anion.

**Figure 11:**
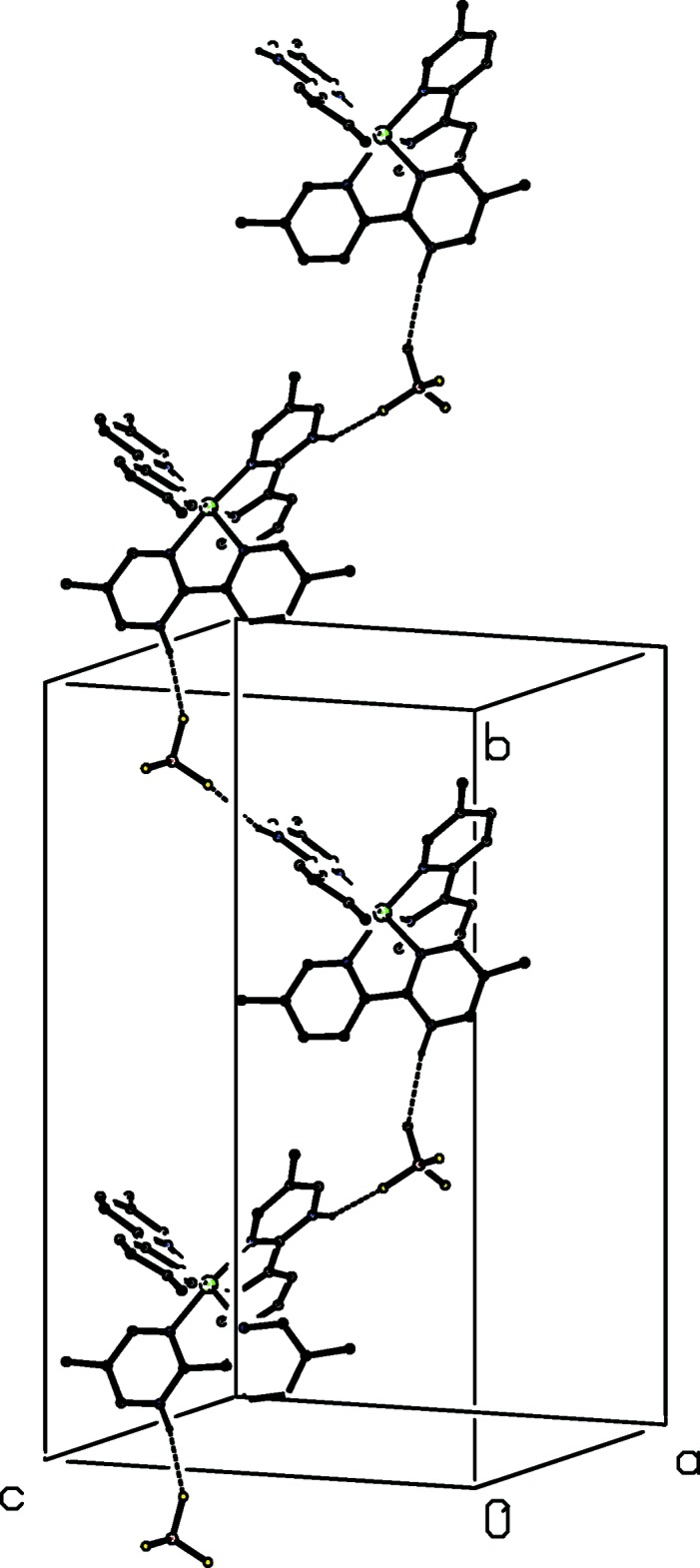
Part of the crystal structure of compound (IV) showing the formation of a hydrogen-bonded 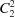
(12) chain running parallel to the [010] direction. For the sake of clarity, the tcnoet anion and the H atoms not involved in the motif shown have been omitted.

**Figure 12:**
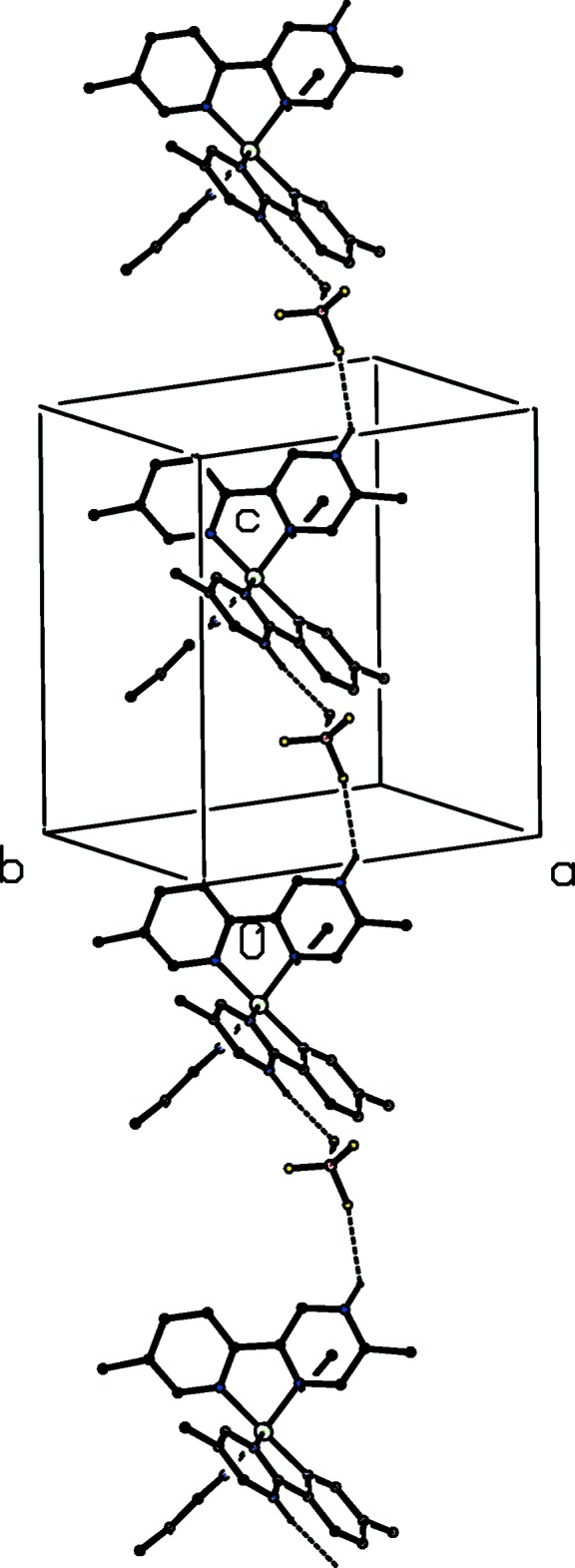
Part of the crystal structure of compound (VI) showing the formation of a hydrogen-bonded 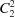
(12) chain running parallel to the [001] direction. For the sake of clarity, the tcnopr anion and the H atoms not involved in the motif shown have been omitted.

A similar type of 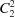
(12) chain is formed in each of compounds (IV) and (V), but now the cation–tetra­fluorido­borate aggregates are related by the 2_1_ screw axis along (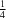, *y*, 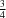) (Fig. 11 ▸): the tcnoet anion in (IV) and the tcnset anion in (V) are pendent from this type of chain but play no other part in the aggregation. The cation-tetra­fluorido­borate chain in compound (VI) is of the 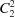
(13) type, built from aggregates related by translation along the [001] direction (Fig. 12 ▸): again the polycyano anion is simply pendent from this chain.

The inter­actions between aromatic rings and both covalent C—Cl bonds and chloride ions have recently been reviewed (Imai *et al.*, 2008 ▸; Schottel *et al.*, 2008 ▸), and the consensus from a range of experimental and computational studies indicates that aryl-Cl⋯centroid distances cluster around 3.6 Å while Cl^−^⋯centroid distances cluster around 3.1 Å, and F⋯centroid distances lie in the range 2.7–2.9 Å. Although no systematic studies have been made on N-containing anions, it is probable that optimal N⋯centroid distances in such systems will be less than the covalent C—Cl⋯centroid opti­mum distance of 3.6 Å. Thus, in the tris­(phen­ethroline)iron(II) salt with the anion (tcn*X*)^−^ where *X* here represents the 2-hydoxyeth­oxy group (incorrectly described in the original report as 2-hy­droxy­eth­yl), one of the cyano groups forms contacts with two different pyridyl rings within the selected asymmetric unit, with N⋯centroid distances of 3.212 (2) and 3.418 (2) Å (Setifi, Domasevitch *et al.*, 2013 ▸). Here we have limited our attention to tncn*X*⋯centroid contacts (where *X* represents an alk­oxy or alkyl­sulfanyl group) of less than 3.4 Å (Table 2 ▸). On this basis there are significant anion⋯π inter­actions only in compounds (II) and (III): in (II), two such inter­actions link the cations and the major disorder component of the tcn*X* anion into a centrosymmetric four-ion aggregate, while in compound (III), the sole inter­action of this type does not lead to any continuous aggregation as there are no hydrogen bonds between the cation and the polycyano anion (Table 1 ▸).

## Database survey   

The structures of compounds containing tcn*X* anions have been reported in recent years for a variety of systems, including complexes of cadmium (Setifi, Morgenstern *et al.*, 2017 ▸), copper (Setifi, Setifi, El-Ammari *et al.*, 2014 ▸; Addala *et al.*, 2015 ▸) and iron (Setifi *et al.*, 2010 ▸; Setifi, Domasevitch *et al.*, 2013 ▸; Setifi, Setifi *et al.*, 2013 ▸; Setifi, Setifi, Boughzala *et al.*, 2014 ▸), as well as salts of purely organic cations mostly based on polypyridines (Setifi, Lehchili *et al.*, 2014 ▸; Setifi *et al.*, 2015 ▸, 2016 ▸). Only in the complexes do the tcn*X* units acts as ligands, while the occur as free anions in all of the cadmium, iron and polypyridinium salts. In all of these salts, as in compounds (I)–(VI) reported here, the bond distances in the anions indicate delocalization of the negative charge over the whole of the tetra­cyano­propenide skeleton of the anion.

## Synthesis and crystallization   

All chemical reagents and solvents are commercially available and were used without further purification. For the synthesis of compounds (III)–(VI), mixtures of 5,5′-dimethyl-2,2′-bi­pyridine (18.4 mg, 0.1 mmol), iron(II) tetra­fluorido­borate hexa­hydrate (33.8 mg, 0.1 mmol), and 0.2 mmol of the appropriate polynitrile salt: [K(tcnome) for (III), K(tcnoet) for (IV), K(tcnset) for (V) or K(tcnopr) for (VI)] in water–ethanol (4:1 *v*/*v*, 20 cm^3^) were heated at 423 K for 3 d in a sealed Teflon-lined stainless steel vessel under autogenous pressure and then cooled gradually to room temperature at a rate of 10 K h^−1^. After the reaction vessels had cooled to ambient temperature, crystals suitable for single-crystal X-ray diffraction were collected by filtration and dried in air. For the synthesis of compounds (I) and (II), a similar procedure was employed using 0.1 mmol of 2,2′-bi­pyridine, 0.1 mmol of iron(II) perchlorate hexa­hydrate and either 0.2 mmol of tcnome, for (I), or tcnspr, for (II).

## Refinement   

Crystal data, data collection and structure refinement details are summarized in Table 3 ▸. Apart from the isostructural pair of compounds (IV) and (V), it was apparent at an early stage in the refinements that there was extensive disorder in the anionic components, although the cations were all fully ordered: in each of (I)–(VI), the asymmetric unit was selected such that the reference cation was the one having the Δ configuration. Several low-angle reflections which had been atten­uated by the beam stop were omitted from the final refinements: (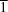
01) and (021) for (IV), and (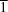
21) for (V). Similarly, some bad outlier reflections were omitted:[Chem scheme1] (186) and (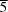
71) for (III), and (354), (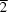
42), (344), (528), (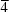
54) and (628) for (IV). In compound (I), one of the tcnome anions, that containing atom O721, exhibits orientational disorder of one of the C(CN)_2_ units over two sets of atomic sites, while the other anion exhibits disorder of the whole anion, again over two sets of atomic sites. The tcnspr anion in compound (II) is disordered over two sets of atomic sites, while the perchlorate anion was found to be disordered over three sets of sites. In compound (III), the tcnome anion is fully ordered but the tetra­fluorido­borate anion is disordered over two sets of sites, whereas in (VI), the tetra­fluorido­borate anion is fully ordered but the tcnopr anion is disordered over two sets of sites. For compounds (IV) and (V), all H atoms were located in difference maps and then treated as riding atoms in geometrically idealized positions with C—H distances of 0.95 Å (pyrid­yl), 0.98 Å (CH_3_) or 0.99 Å (CH_2_) and with *U*_iso_(H) = *kU*_eq_(C), where *k* = 1.5 for the methyl groups, which were permitted to rotate but not to tilt, and 1.2 for all other H atoms. The H atoms bonded to C atoms in compounds (I)–(III) and (VI) were included in the calculations on the same basis. For the H atoms in the water component of compound (I), the atomic coordinates were refined, with *U*_iso_(H) = 1.5*U*_eq_(O), giving O—H distances of 0.96 (2) Å. For each of the disordered components, the bonded distances and the (1,3) non-bonded distances of the minor components were restrained to be equal to those of the corresponding major components, subject to s.u. values of 0.005 and 0.01 Å, respectively. In addition, the anisotropic displacement parameters of corresponding pairs of atoms were constrained to be identical. On this basis, the refined occupancies for the two anions in (I) were 0.500 (7) and 0.500 (7) in one anion and 0.502 (2) and 0.498 (2) in the other, so that thereafter these occupancies were all fixed at 0.5: the refined occupancy for the water component in the crystal selected for data collection was 0.776 (6). The refined tcnspr occupancies in (II) were 0.754 (2) and 0.246 (2), with perchlorate occupancies of 0.439 (3), 0.277 (3) and 0.184 (3). The refined tetra­fluorido­borate occupancies in (III) were 0.671 (4) and 0.329 (4), while the tcnopr occupancies in (VI) were 0.508 (6) and 0.492 (6). The largest peak in the difference map for compound (II) was located close to atom N832 of occupancy 0.246 (2). After the final refinement for (II), there was a large residual density, 2.23 Å^−3^, situated 1.03 Å from atom N832 and 1.05 Å from atom C832 [occupancies 0.246 (2)].

## Supplementary Material

Crystal structure: contains datablock(s) global, I, II, III, IV, V, VI. DOI: 10.1107/S2056989018015426/sj5565sup1.cif

Structure factors: contains datablock(s) I. DOI: 10.1107/S2056989018015426/sj5565Isup2.hkl

Structure factors: contains datablock(s) II. DOI: 10.1107/S2056989018015426/sj5565IIsup3.hkl

Structure factors: contains datablock(s) III. DOI: 10.1107/S2056989018015426/sj5565IIIsup4.hkl

Structure factors: contains datablock(s) IV. DOI: 10.1107/S2056989018015426/sj5565IVsup5.hkl

Structure factors: contains datablock(s) V. DOI: 10.1107/S2056989018015426/sj5565Vsup6.hkl

Structure factors: contains datablock(s) VI. DOI: 10.1107/S2056989018015426/sj5565VIsup7.hkl

CCDC references: 1876478, 1876479, 1876480, 1876481, 1876482, 1876483

Additional supporting information:  crystallographic information; 3D view; checkCIF report

## Figures and Tables

**Figure 2:**
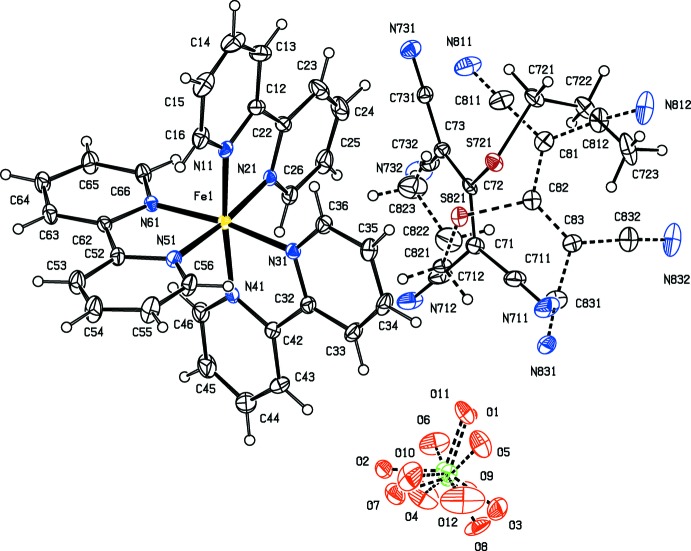
The independent ionic components in compound (II), showing the atom-labelling scheme. Displacement ellipsoids are drawn at the 30% probability level.

**Figure 3:**
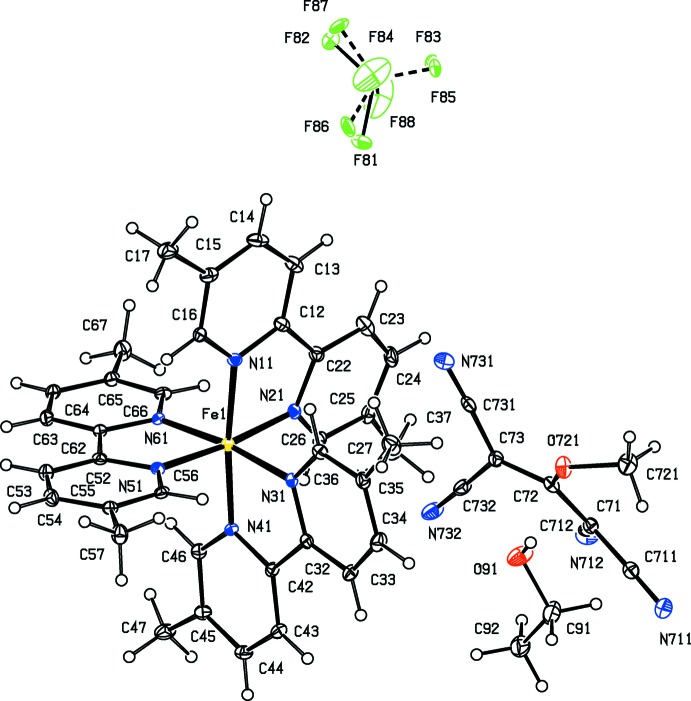
The independent ionic components in compound (III), showing the atom-labelling scheme. Displacement ellipsoids are drawn at the 30% probability level.

**Figure 4:**
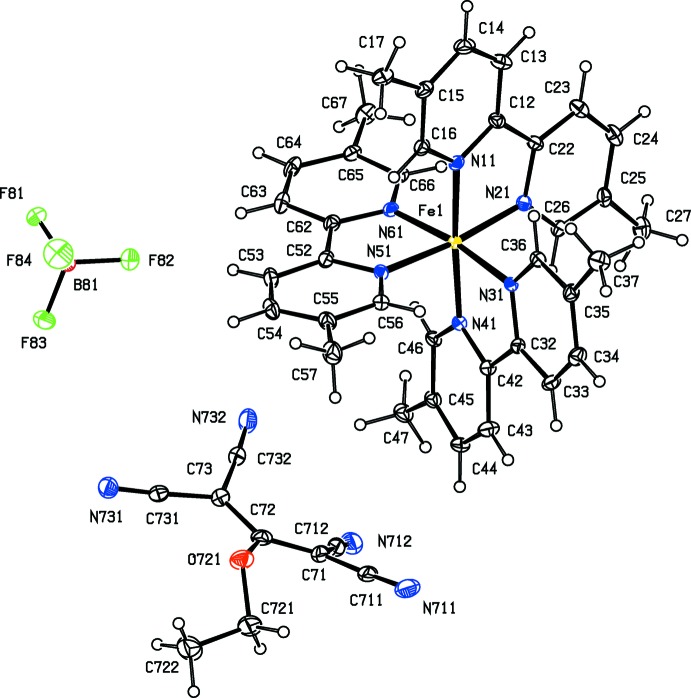
The independent ionic components in compound (IV), showing the atom-labelling scheme. Displacement ellipsoids are drawn at the 30% probability level.

**Figure 5:**
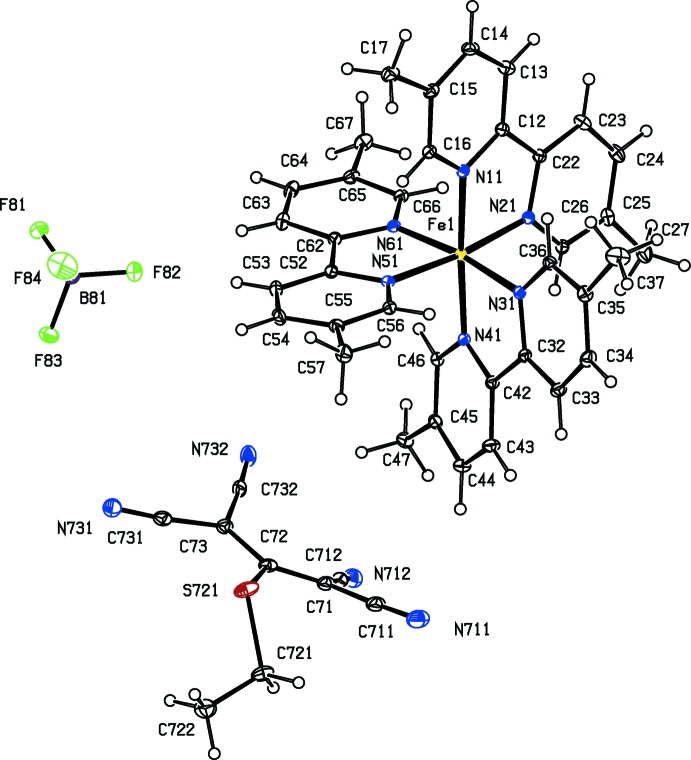
The independent ionic components in compound (V), showing the atom-labelling scheme. Displacement ellipsoids are drawn at the 30% probability level.

**Figure 6:**
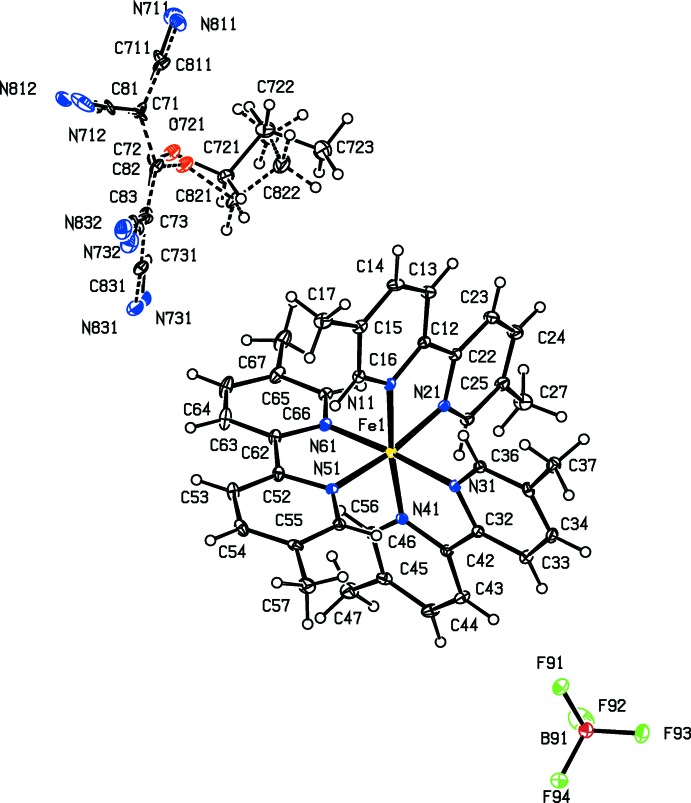
The independent ionic components in compound (VI), showing the atom-labelling scheme. Displacement ellipsoids are drawn at the 30% probability level.

**Table 1:** Hydrogen bonds and short intra- and inter­molecular contacts (Å, °)

Compound	*D*—H⋯*A*		*D*—H	H⋯*A*	*D*⋯*A*	*D*—H⋯*A*
(I)	C34—H34⋯N742		0.95	2.62	3.51 (3)	156
	C43—H43⋯N741		0.95	2.59	3.525 (7)	170
	C53—H53⋯N811		0.95	2.58	3.496 (15)	161
	C63—H63⋯N811		0.95	2.47	3.329 (16)	151
	C63—H63⋯N932		0.95	2.59	3.434 (16)	148
	C66—H66⋯O101		0.95	2.49	3.297 (3)	142
	C25—H25⋯N831^i^		0.95	2.48	3.398 (3)	162
	C54—H54⋯N742^ii^		0.95	2.61	3.51 (3)	157
	O101—H101⋯N812^iii^		0.96 (2)	2.23 (3)	3.143 (4)	159 (2)
	O101—H101⋯N912^iii^		0.96 (2)	2.13 (3)	3.085 (5)	175 (3)
	O101—H102⋯N832^iv^		0.95 (3)	2.13 (3)	3.017 (12)	154 (3)
	O101—H102⋯N911^iv^		0.95 (3)	2.02 (3)	2.931 (14)	161 (3)
(II)	C15—H15⋯N832^v^		0.95	2.50	3.267 (13)	138
	C24—H24⋯N731		0.95	2.59	3.471 (6)	154
	C35—H35⋯N712^vi^		0.95	2.57	3.207 (7)	125
	C54—H54⋯N812^vii^		0.95	2.54	3.215 (15)	128
	C13—H13⋯O7^viii^		0.95	2.34	3.258 (10)	163
	C33—H33⋯O10		0.95	2.41	3.351 (17)	172
	C43—H43⋯O10		0.95	2.57	3.521 (17)	174
	C53—H53⋯O3^ix^		0.95	2.51	3.432 (9)	165
	C63—H63⋯O5^ix^		0.95	2.59	3.512 (8)	163
(III)	O91—H91⋯N712		0.84	2.11	2.895 (5)	156
	C13—H13⋯F81		0.95	2.45	3.298 (4)	149
	C43—H43⋯F87^*x*^		0.95	2.40	3.277 (6)	154
	C63—H63⋯F83^ix^		0.95	2.50	3.276 (4)	138
	C63—H63⋯F85^ix^		0.95	2.39	3.330 (6)	170
(IV)	C23—H23⋯F81^xi^		0.95	2.38	3.259 (4)	154
	C44—H44⋯N711		0.95	2.58	3.461 (5)	155
	C53—H53⋯F82		0.95	2.40	3.342 (4)	171
(V)	C23—H23⋯F81^xi^		0.95	2.40	3.3206 (18)	163
	C44—H44⋯N711		0.95	2.67	3.582 (2)	161
	C53—H53⋯F82		0.95	2.41	3.3598 (18)	176
(VI)	C43—H43⋯F91		0.95	2.37	3.308 (3)	170
	C54—H54⋯F93^xii^		0.95	2.54	3.316 (3)	139
	C64—H64⋯N831		0.95	2.54	3.414 (7)	154

Symmetry codes: (i) 1 − *x*, 1 − *y*, 1 − *z*; (ii) *x*, 1 + *y*, *z*; (iii) 1 − *x*, −1 + *y*, 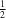 − *z*; (iv) *x*, 2 − *y*, −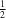 + *z*; (v) −1 + *x*, *y*, −1 + *z*; (vi) −1 + *x*, *y*, *z*; (vii) 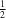 − *x*, −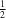 + *y*, 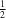 − *z*; (viii) 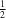 − *x*, 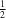 + *y*, 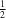 − *z*; (ix) *x*, *y*, −1 + *z*; (*x*) 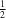 − *x*, −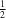 + *y*, 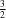 − *z*; (xi) 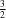 − *x*, 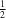 + *y*, 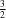 − *z*; (xii) *x*, *y*, 1 + *z*.

**Table 2:** Parameters (Å, °) for C—N⋯π contacts in compounds (II) and (III) *Cg*1, *Cg*2 and *Cg*3 represent the centroids of the rings (N11, C12–C16), (N61, C62–C66) and (N31, C32–C36) respectively.

Compound	C—N⋯*Cg*		N⋯*Cg*	C⋯*Cg*	C—N⋯*Cg*
(II)	C731—N731⋯*Cg*1^i^		3.186 (5)	3.640 (4)	104.0 (3)
	C731—N731⋯*Cg*2^i^		3.023 (4)	4.077 (5)	152.3 (4)
	C812—N812⋯*Cg*3^ii^		3.105 (14)	3.873 (16)	124.9 (13)
(III)	C711—N711⋯*Cg*2^iii^		3.088 (3)	4.092 (4)	145.5 (2)

Symmetry codes: (i) 1 − *x*, 1 − *y*, −*z*; (ii) 1 − *x*, 1 − *y*, 1 − *z*; (iii) 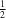 − *x*, −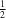 + *y*, 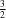 − *z*.

**Table:** 

	(I)	(II)	(III)
Crystal data			
Chemical formula	[Fe(C_10_H_8_N_2_)_3_](C_8_H_3_N_4_O)_2_·0.776H_2_O	[Fe(C_10_H_8_N_2_)_3_](C_10_H_7_N_4_S)(ClO_4_)	[Fe(C_12_H_12_N_2_)_3_](C_8_H_3_N_4_O)(BF_4_)·0.926C_2_H_2_O
*M*_r_	880.65	839.11	909.18
Crystal system, space group	Monoclinic, *C*2/*c*	Monoclinic, *P*2_1_/*n*	Monoclinic, *P*2_1_/*n*
Temperature (K)	100	100	100
*a*, *b*, *c* (Å)	38.3410 (3), 11.2756 (1), 19.33740 (16)	11.6644 (3), 23.1692 (4), 13.9599 (3)	11.6979 (4), 25.7716 (7), 14.1055 (4)
α, β, γ (°)	90, 97.503 (1), 90	90, 97.202 (2), 90	90, 100.444 (3), 90
*V* (Å^3^)	8288.32 (12)	3742.96 (14)	4182.0 (2)
*Z*	8	4	4
Radiation type	Cu *K*α	Mo *K*α	Mo *K*α
μ (mm^−1^)	3.42	0.59	0.43
Crystal size (mm)	0.15 × 0.05 × 0.02	0.24 × 0.22 × 0.17	0.29 × 0.24 × 0.20

Data collection			
Diffractometer	Rigaku XtaLAB Synergy-S	Rigaku SuperNova, Single source at offset, Eos	Rigaku SuperNova, Single source at offset, Eos
Absorption correction	Multi-scan (*CrysAlis PRO*; Rigaku OD, 2015 ▸)	Multi-scan (*CrysAlis PRO*; Rigaku OD, 2015 ▸)	Multi-scan (*CrysAlis PRO*; Rigaku OD, 2015 ▸)
*T*_min_, *T*_max_	0.845, 0.934	0.724, 0.905	0.540, 0.917
No. of measured, independent and observed [*I* > 2σ(*I*)] reflections	26027, 7579, 6565	30922, 8586, 5903	32301, 8711, 5956
*R*_int_	0.042	0.056	0.090
(sin θ/λ)_max_ (Å^−1^)	0.602	0.667	0.629

Refinement			
*R*[*F* ^2^ > 2σ(*F* ^2^)], *wR*(*F* ^2^), *S*	0.033, 0.081, 1.05	0.059, 0.170, 1.05	0.062, 0.123, 1.05
No. of reflections	7579	8586	8711
No. of parameters	694	721	627
No. of restraints	560	151	10
H-atom treatment	H atoms treated by a mixture of independent and constrained refinement	H-atom parameters constrained	H-atom parameters constrained
Δρ_max_, Δρ_min_ (e Å^−3^)	0.22, −0.38	2.23, −0.42	0.46, −0.50

**Table:** 

	(IV)	(V)	(VI)
Crystal data			
Chemical formula	[Fe(C_12_H_12_N_2_)_3_](C_9_H_5_N_4_O)(BF_4_)	[Fe(C_12_H_12_N_2_)_3_](C_9_H_5_N_4_S)(BF_4_)	[Fe(C_12_H_12_N_2_)_3_](C_10_H_7_N_4_O)(BF_4_)
*M*_r_	880.54	896.60	894.56
Crystal system, space group	Monoclinic, *P*2_1_/*n*	Monoclinic, *P*2_1_/*n*	Triclinic, *P* 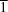
Temperature (K)	100	100	100
*a*, *b*, *c* (Å)	11.5865 (3), 25.5914 (5), 14.4997 (3)	11.6027 (5), 25.0774 (10), 14.7438 (6)	11.6246 (5), 14.2404 (6), 14.3224 (6)
α, β, γ (°)	90, 104.641 (3), 90	90, 104.211 (2), 90	65.340 (2), 76.040 (3), 87.571 (3)
*V* (Å^3^)	4159.77 (17)	4158.7 (3)	2086.49 (16)
*Z*	4	4	2
Radiation type	Cu *K*α	Ga *K*α, λ = 1.34139 Å	Ga *K*α, λ = 1.34139 Å
μ (mm^−1^)	3.48	2.67	2.37
Crystal size (mm)	0.14 × 0.03 × 0.02	0.13 × 0.11 × 0.03	0.06 × 0.03 × 0.03

Data collection			
Diffractometer	Rigaku XtaLAB Synergy-S	Bruker Venture Metaljet	Bruker Venture Metaljet
Absorption correction	Multi-scan (*CrysAlis PRO*; Rigaku OD, 2015 ▸)	Multi-scan (*SADABS*; Bruker, 2014 ▸)	Multi-scan (*SADABS*; Bruker, 2014 ▸)
*T*_min_, *T*_max_	0.746, 0.920	0.832, 0.923	0.868, 0.931
No. of measured, independent and observed [*I* > 2σ(*I*)] reflections	30853, 7607, 5392	64342, 9563, 8430	60005, 9584, 7914
*R*_int_	0.079	0.037	0.052
(sin θ/λ)_max_ (Å^−1^)	0.602	0.650	0.650

Refinement			
*R*[*F* ^2^ > 2σ(*F* ^2^)], *wR*(*F* ^2^), *S*	0.059, 0.162, 1.02	0.033, 0.086, 1.04	0.045, 0.111, 1.08
No. of reflections	7607	9563	9584
No. of parameters	566	566	712
No. of restraints	0	0	30
H-atom treatment	H-atom parameters constrained	H-atom parameters constrained	H-atom parameters constrained
Δρ_max_, Δρ_min_ (e Å^−3^)	1.71, −0.45	0.40, −0.35	0.68, −0.37

Computer programs: *CrysAlis PRO* (Rigaku OD, 2015 ▸), *APEX2* and *SAINT* (Bruker, 2013 ▸), *SHELXT* (Sheldrick, 2015*a* ▸), *Olex2.solve* (Dolomanov *et al.*, 2009 ▸), *SHELXL2014* (Sheldrick, 2015*b* ▸) and *PLATON* (Spek, 2009 ▸).

## supplementary crystallographic information

### Tris(2,2'-bipyridine)iron(II) bis(1,1,3,3-tetracyano-2-methoxypropenide) 0.776-hydrate (I) Crystal data

**Table:**

[Fe(C_10_H_8_N_2_)_3_](C_8_H_3_N_4_O)_2_·0.776H_2_O	*F*(000) = 3630
*M**_r_* = 880.65	*D*_x_ = 1.411 Mg m^−^^3^
Monoclinic, *C*2/*c*	Cu *K*α radiation, λ = 1.54184 Å
*a* = 38.3410 (3) Å	Cell parameters from 7579 reflections
*b* = 11.2756 (1) Å	θ = 4.1–68.2°
*c* = 19.33740 (16) Å	µ = 3.42 mm^−^^1^
β = 97.503 (1)°	*T* = 100 K
*V* = 8288.32 (12) Å^3^	Needle, red
*Z* = 8	0.15 × 0.05 × 0.02 mm

### Tris(2,2'-bipyridine)iron(II) bis(1,1,3,3-tetracyano-2-methoxypropenide) 0.776-hydrate (I) Data collection

**Table:**

Rigaku XtaLAB Synergy-S diffractometer	7579 independent reflections
Radiation source: sealed tube	6565 reflections with *I* > 2σ(*I*)
Detector resolution: 5.811 pixels mm^-1^	*R*_int_ = 0.042
ω scans	θ_max_ = 68.2°, θ_min_ = 4.1°
Absorption correction: multi-scan (CrysAlis PRO; Rigaku OD, 2015)	*h* = −37→46
*T*_min_ = 0.845, *T*_max_ = 0.934	*k* = −13→11
26027 measured reflections	*l* = −23→23

### Tris(2,2'-bipyridine)iron(II) bis(1,1,3,3-tetracyano-2-methoxypropenide) 0.776-hydrate (I) Refinement

**Table:**

Refinement on *F*^2^	560 restraints
Least-squares matrix: full	Hydrogen site location: mixed
*R*[*F*^2^ > 2σ(*F*^2^)] = 0.033	H atoms treated by a mixture of independent and constrained refinement
*wR*(*F*^2^) = 0.081	*w* = 1/[σ^2^(*F*_o_^2^) + (0.0369*P*)^2^ + 3.4626*P*] where *P* = (*F*_o_^2^ + 2*F*_c_^2^)/3
*S* = 1.05	(Δ/σ)_max_ = 0.004
7579 reflections	Δρ_max_ = 0.22 e Å^−^^3^
694 parameters	Δρ_min_ = −0.38 e Å^−^^3^

### Tris(2,2'-bipyridine)iron(II) bis(1,1,3,3-tetracyano-2-methoxypropenide) 0.776-hydrate (I) Special details

**Table:**

Geometry. All esds (except the esd in the dihedral angle between two l.s. planes) are estimated using the full covariance matrix. The cell esds are taken into account individually in the estimation of esds in distances, angles and torsion angles; correlations between esds in cell parameters are only used when they are defined by crystal symmetry. An approximate (isotropic) treatment of cell esds is used for estimating esds involving l.s. planes.

### Tris(2,2'-bipyridine)iron(II) bis(1,1,3,3-tetracyano-2-methoxypropenide) 0.776-hydrate (I) Fractional atomic coordinates and isotropic or equivalent isotropic displacement parameters (Å^2^)

**Table:**

	*x*	*y*	*z*	*U*_iso_*/*U*_eq_	Occ. (<1)
Fe1	0.63020 (2)	0.51545 (2)	0.36805 (2)	0.01784 (8)	
N11	0.65493 (3)	0.53184 (12)	0.28524 (7)	0.0198 (3)	
C12	0.64166 (4)	0.46533 (14)	0.22925 (9)	0.0220 (3)	
C13	0.65452 (5)	0.47382 (16)	0.16571 (9)	0.0281 (4)	
H13	0.6449	0.4259	0.1275	0.034*	
C14	0.68138 (5)	0.55208 (17)	0.15817 (9)	0.0292 (4)	
H14	0.6900	0.5603	0.1146	0.035*	
C15	0.69551 (4)	0.61845 (16)	0.21524 (9)	0.0277 (4)	
H15	0.7144	0.6719	0.2117	0.033*	
C16	0.68182 (4)	0.60589 (15)	0.27739 (9)	0.0237 (3)	
H16	0.6918	0.6514	0.3164	0.028*	
N21	0.60283 (3)	0.39919 (12)	0.30723 (7)	0.0229 (3)	
C22	0.61261 (4)	0.38738 (15)	0.24245 (9)	0.0244 (4)	
C23	0.59581 (5)	0.30908 (17)	0.19369 (11)	0.0337 (4)	
H23	0.6033	0.3020	0.1490	0.040*	
C24	0.56809 (5)	0.24158 (18)	0.21051 (12)	0.0410 (5)	
H24	0.5563	0.1873	0.1778	0.049*	
C25	0.55784 (5)	0.25471 (18)	0.27583 (12)	0.0387 (5)	
H25	0.5386	0.2102	0.2885	0.046*	
C26	0.57570 (5)	0.33299 (16)	0.32268 (10)	0.0299 (4)	
H26	0.5685	0.3404	0.3677	0.036*	
N31	0.66037 (3)	0.38781 (11)	0.41112 (7)	0.0186 (3)	
C32	0.65038 (4)	0.33959 (14)	0.46985 (8)	0.0189 (3)	
C33	0.66942 (4)	0.24931 (15)	0.50592 (9)	0.0224 (3)	
H33	0.6619	0.2170	0.5468	0.027*	
C34	0.69950 (4)	0.20654 (15)	0.48204 (9)	0.0235 (3)	
H34	0.7129	0.1449	0.5062	0.028*	
C35	0.70956 (4)	0.25577 (15)	0.42208 (9)	0.0238 (4)	
H35	0.7301	0.2283	0.4045	0.029*	
C36	0.68950 (4)	0.34510 (15)	0.38806 (9)	0.0214 (3)	
H36	0.6966	0.3777	0.3468	0.026*	
N41	0.60540 (3)	0.48407 (12)	0.44904 (7)	0.0215 (3)	
C42	0.61868 (4)	0.39386 (14)	0.49126 (8)	0.0199 (3)	
C43	0.60337 (4)	0.35995 (15)	0.54935 (8)	0.0224 (3)	
H43	0.6128	0.2955	0.5774	0.027*	
C44	0.57418 (4)	0.42113 (16)	0.56607 (9)	0.0254 (4)	
H44	0.5635	0.3997	0.6059	0.030*	
C45	0.56094 (5)	0.51383 (16)	0.52369 (10)	0.0297 (4)	
H45	0.5411	0.5575	0.5342	0.036*	
C46	0.57693 (4)	0.54205 (16)	0.46596 (10)	0.0281 (4)	
H46	0.5675	0.6052	0.4368	0.034*	
N51	0.65666 (3)	0.64356 (12)	0.42118 (7)	0.0197 (3)	
C52	0.64292 (4)	0.75371 (14)	0.40960 (8)	0.0200 (3)	
C53	0.65725 (4)	0.85275 (15)	0.44540 (9)	0.0245 (4)	
H53	0.6470	0.9288	0.4364	0.029*	
C54	0.68670 (4)	0.83901 (16)	0.49433 (9)	0.0269 (4)	
H54	0.6969	0.9053	0.5197	0.032*	
C55	0.70104 (4)	0.72676 (16)	0.50567 (9)	0.0278 (4)	
H55	0.7213	0.7154	0.5388	0.033*	
C56	0.68566 (4)	0.63181 (15)	0.46859 (9)	0.0240 (4)	
H56	0.6958	0.5553	0.4766	0.029*	
N61	0.60056 (3)	0.64995 (12)	0.33182 (7)	0.0203 (3)	
C62	0.61195 (4)	0.75802 (14)	0.35579 (8)	0.0203 (3)	
C63	0.59599 (4)	0.86231 (15)	0.33035 (9)	0.0246 (4)	
H63	0.6045	0.9368	0.3482	0.030*	
C64	0.56748 (5)	0.85656 (16)	0.27866 (9)	0.0277 (4)	
H64	0.5564	0.9270	0.2598	0.033*	
C65	0.55536 (4)	0.74666 (16)	0.25490 (9)	0.0266 (4)	
H65	0.5356	0.7405	0.2199	0.032*	
C66	0.57225 (4)	0.64599 (16)	0.28246 (9)	0.0249 (4)	
H66	0.5636	0.5708	0.2660	0.030*	
C72	0.69853 (4)	0.03189 (15)	0.76960 (9)	0.0233 (3)	
C73	0.73040 (4)	0.06663 (15)	0.80710 (9)	0.0230 (3)	
C71	0.69130 (5)	0.03611 (19)	0.69654 (10)	0.0349 (4)	0.5
C711	0.65742 (9)	0.0407 (5)	0.6542 (2)	0.0373 (6)	0.5
N711	0.63014 (13)	0.0548 (6)	0.6228 (3)	0.0524 (14)	0.5
C712	0.72008 (14)	0.0137 (8)	0.6580 (3)	0.0373 (6)	0.5
N712	0.7426 (5)	−0.004 (3)	0.6258 (10)	0.048 (3)	0.5
C74	0.69130 (5)	0.03611 (19)	0.69654 (10)	0.0349 (4)	0.5
C741	0.65633 (9)	0.0724 (5)	0.6693 (2)	0.0373 (6)	0.5
N741	0.62855 (12)	0.0995 (6)	0.6437 (3)	0.0524 (14)	0.5
C742	0.71740 (14)	0.0318 (8)	0.6508 (3)	0.0373 (6)	0.5
N742	0.7387 (5)	0.019 (3)	0.6148 (10)	0.048 (3)	0.5
O721	0.67432 (3)	−0.00543 (11)	0.80958 (6)	0.0262 (3)	
C721	0.65061 (4)	−0.10027 (16)	0.78297 (9)	0.0270 (4)	
H72A	0.6314	−0.0674	0.7501	0.032*	
H72B	0.6409	−0.1382	0.8218	0.032*	
H72C	0.6636	−0.1591	0.7591	0.032*	
C731	0.73998 (4)	0.03020 (15)	0.87735 (9)	0.0241 (4)	
N731	0.74938 (4)	0.00079 (15)	0.93384 (8)	0.0310 (4)	
C732	0.75460 (4)	0.14209 (15)	0.77815 (9)	0.0244 (4)	
N732	0.77397 (4)	0.20489 (14)	0.75575 (8)	0.0315 (3)	
C81	0.54650 (7)	1.1235 (3)	0.48817 (15)	0.0176 (6)	0.5
C82	0.5402 (5)	1.0580 (9)	0.5457 (7)	0.013 (2)	0.5
C83	0.51553 (8)	1.0960 (3)	0.59211 (15)	0.0171 (6)	0.5
C811	0.5778 (3)	1.1118 (6)	0.4577 (6)	0.0172 (11)	0.5
N811	0.6031 (3)	1.1034 (14)	0.4306 (8)	0.0236 (19)	0.5
C812	0.5233 (6)	1.218 (2)	0.4605 (12)	0.022 (2)	0.5
N812	0.50462 (10)	1.2848 (3)	0.4312 (2)	0.0283 (8)	0.5
O821	0.55423 (6)	0.95522 (19)	0.56635 (11)	0.0203 (5)	0.5
C821	0.5713 (3)	0.8826 (8)	0.5190 (4)	0.0209 (17)	0.5
H82A	0.5611	0.8998	0.4709	0.031*	0.5
H82B	0.5677	0.7987	0.5292	0.031*	0.5
H82C	0.5965	0.9002	0.5249	0.031*	0.5
C831	0.5074 (3)	1.0183 (11)	0.6457 (6)	0.0188 (17)	0.5
N831	0.50032 (7)	0.9583 (3)	0.69043 (14)	0.0228 (6)	0.5
C832	0.50228 (16)	1.2143 (5)	0.5954 (2)	0.0229 (8)	0.5
N832	0.4923 (4)	1.3088 (9)	0.5996 (7)	0.0245 (16)	0.5
C91	0.51837 (8)	1.1779 (3)	0.52930 (15)	0.0211 (7)	0.5
C92	0.5362 (5)	1.0758 (9)	0.5529 (7)	0.017 (3)	0.5
C93	0.56295 (8)	1.0217 (3)	0.51734 (16)	0.0199 (7)	0.5
C911	0.50149 (16)	1.2498 (5)	0.5745 (3)	0.0229 (8)	0.5
N911	0.4875 (4)	1.3128 (12)	0.6101 (7)	0.038 (3)	0.5
C912	0.5162 (6)	1.217 (2)	0.4576 (12)	0.022 (2)	0.5
N912	0.51267 (10)	1.2623 (4)	0.40475 (18)	0.0280 (9)	0.5
O921	0.53230 (6)	1.0179 (2)	0.61084 (11)	0.0255 (5)	0.5
C921	0.4991 (2)	1.0262 (12)	0.6387 (6)	0.022 (2)	0.5
H92A	0.4999	1.0929	0.6713	0.032*	0.5
H92B	0.4948	0.9525	0.6631	0.032*	0.5
H92C	0.4800	1.0389	0.6004	0.032*	0.5
C931	0.5753 (3)	0.9057 (8)	0.5361 (4)	0.0264 (18)	0.5
N931	0.58600 (10)	0.8107 (3)	0.54668 (17)	0.0386 (8)	0.5
C932	0.5802 (3)	1.0784 (6)	0.4647 (6)	0.0172 (11)	0.5
N932	0.5952 (3)	1.1207 (14)	0.4241 (8)	0.0235 (18)	0.5
O101	0.51547 (6)	0.47541 (18)	0.18585 (11)	0.0536 (8)	0.776 (6)
H101	0.5074 (9)	0.4061 (16)	0.1599 (16)	0.080*	0.776 (6)
H102	0.5013 (8)	0.5380 (19)	0.1643 (17)	0.080*	0.776 (6)

### Tris(2,2'-bipyridine)iron(II) bis(1,1,3,3-tetracyano-2-methoxypropenide) 0.776-hydrate (I) Atomic displacement parameters (Å^2^)

**Table:**

	*U*^11^	*U*^22^	*U*^33^	*U*^12^	*U*^13^	*U*^23^
Fe1	0.01377 (12)	0.01514 (13)	0.02387 (14)	−0.00043 (9)	−0.00034 (9)	0.00311 (10)
N11	0.0178 (6)	0.0160 (7)	0.0242 (7)	0.0018 (5)	−0.0028 (5)	0.0031 (5)
C12	0.0188 (8)	0.0189 (8)	0.0266 (9)	0.0049 (6)	−0.0034 (6)	0.0015 (7)
C13	0.0257 (9)	0.0309 (10)	0.0262 (9)	0.0079 (7)	−0.0021 (7)	−0.0021 (7)
C14	0.0255 (9)	0.0367 (11)	0.0255 (9)	0.0088 (8)	0.0037 (7)	0.0069 (8)
C15	0.0237 (8)	0.0256 (9)	0.0342 (9)	0.0010 (7)	0.0055 (7)	0.0064 (8)
C16	0.0200 (8)	0.0204 (9)	0.0301 (9)	−0.0022 (6)	0.0008 (7)	0.0021 (7)
N21	0.0180 (7)	0.0166 (7)	0.0323 (8)	−0.0008 (5)	−0.0033 (6)	0.0050 (6)
C22	0.0212 (8)	0.0194 (8)	0.0308 (9)	0.0020 (6)	−0.0030 (7)	0.0015 (7)
C23	0.0314 (10)	0.0280 (10)	0.0391 (11)	−0.0025 (8)	−0.0053 (8)	−0.0060 (8)
C24	0.0376 (11)	0.0311 (11)	0.0501 (13)	−0.0101 (9)	−0.0095 (9)	−0.0060 (9)
C25	0.0302 (10)	0.0282 (10)	0.0540 (13)	−0.0129 (8)	−0.0081 (9)	0.0065 (9)
C26	0.0240 (9)	0.0245 (9)	0.0388 (10)	−0.0070 (7)	−0.0050 (7)	0.0091 (8)
N31	0.0165 (6)	0.0160 (7)	0.0227 (7)	−0.0021 (5)	0.0002 (5)	−0.0006 (5)
C32	0.0176 (7)	0.0165 (8)	0.0219 (8)	−0.0024 (6)	−0.0001 (6)	−0.0006 (6)
C33	0.0232 (8)	0.0198 (8)	0.0238 (8)	0.0000 (6)	0.0012 (6)	0.0015 (7)
C34	0.0223 (8)	0.0199 (8)	0.0273 (9)	0.0039 (6)	−0.0006 (7)	0.0034 (7)
C35	0.0181 (8)	0.0226 (9)	0.0305 (9)	0.0018 (6)	0.0027 (7)	0.0010 (7)
C36	0.0179 (8)	0.0205 (8)	0.0260 (8)	−0.0004 (6)	0.0036 (6)	0.0015 (7)
N41	0.0169 (6)	0.0182 (7)	0.0290 (7)	0.0008 (5)	0.0010 (5)	0.0024 (6)
C42	0.0173 (7)	0.0172 (8)	0.0244 (8)	−0.0021 (6)	−0.0006 (6)	−0.0012 (6)
C43	0.0214 (8)	0.0221 (8)	0.0231 (8)	−0.0026 (6)	0.0011 (6)	0.0008 (7)
C44	0.0219 (8)	0.0272 (9)	0.0278 (9)	−0.0056 (7)	0.0061 (7)	−0.0030 (7)
C45	0.0207 (8)	0.0255 (9)	0.0444 (11)	0.0012 (7)	0.0101 (8)	−0.0012 (8)
C46	0.0201 (8)	0.0222 (9)	0.0428 (11)	0.0052 (7)	0.0076 (7)	0.0070 (8)
N51	0.0167 (6)	0.0196 (7)	0.0226 (7)	0.0007 (5)	0.0018 (5)	0.0029 (5)
C52	0.0197 (7)	0.0188 (8)	0.0218 (8)	0.0009 (6)	0.0047 (6)	0.0027 (6)
C53	0.0250 (8)	0.0185 (8)	0.0300 (9)	0.0006 (7)	0.0039 (7)	0.0009 (7)
C54	0.0272 (9)	0.0219 (9)	0.0308 (9)	−0.0052 (7)	0.0010 (7)	−0.0024 (7)
C55	0.0226 (8)	0.0274 (9)	0.0311 (9)	−0.0028 (7)	−0.0049 (7)	0.0015 (7)
C56	0.0195 (8)	0.0217 (9)	0.0293 (9)	0.0012 (6)	−0.0026 (7)	0.0046 (7)
N61	0.0166 (6)	0.0203 (7)	0.0236 (7)	−0.0002 (5)	0.0016 (5)	0.0031 (6)
C62	0.0177 (8)	0.0211 (8)	0.0224 (8)	0.0015 (6)	0.0043 (6)	0.0035 (6)
C63	0.0264 (9)	0.0195 (9)	0.0281 (9)	0.0039 (7)	0.0043 (7)	0.0022 (7)
C64	0.0274 (9)	0.0272 (10)	0.0287 (9)	0.0104 (7)	0.0037 (7)	0.0085 (7)
C65	0.0221 (8)	0.0310 (10)	0.0259 (9)	0.0052 (7)	−0.0003 (7)	0.0051 (7)
C66	0.0189 (8)	0.0268 (9)	0.0280 (9)	0.0002 (7)	−0.0003 (7)	0.0030 (7)
C72	0.0199 (8)	0.0232 (9)	0.0270 (9)	−0.0017 (6)	0.0040 (6)	0.0049 (7)
C73	0.0224 (8)	0.0250 (9)	0.0221 (8)	−0.0018 (7)	0.0041 (6)	0.0008 (7)
C71	0.0257 (9)	0.0488 (12)	0.0281 (10)	−0.0135 (8)	−0.0040 (7)	0.0142 (9)
C711	0.0372 (9)	0.053 (2)	0.0182 (11)	−0.0224 (9)	−0.0070 (7)	0.0119 (8)
N711	0.0408 (13)	0.074 (5)	0.036 (3)	−0.0212 (19)	−0.0168 (17)	0.022 (2)
C712	0.0372 (9)	0.053 (2)	0.0182 (11)	−0.0224 (9)	−0.0070 (7)	0.0119 (8)
N712	0.046 (3)	0.086 (9)	0.012 (5)	−0.035 (3)	0.000 (4)	0.006 (4)
C74	0.0257 (9)	0.0488 (12)	0.0281 (10)	−0.0135 (8)	−0.0040 (7)	0.0142 (9)
C741	0.0372 (9)	0.053 (2)	0.0182 (11)	−0.0224 (9)	−0.0070 (7)	0.0119 (8)
N741	0.0408 (13)	0.074 (5)	0.036 (3)	−0.0212 (19)	−0.0168 (17)	0.022 (2)
C742	0.0372 (9)	0.053 (2)	0.0182 (11)	−0.0224 (9)	−0.0070 (7)	0.0119 (8)
N742	0.046 (3)	0.086 (9)	0.012 (5)	−0.035 (3)	0.000 (4)	0.006 (4)
O721	0.0206 (6)	0.0299 (7)	0.0288 (6)	−0.0049 (5)	0.0057 (5)	0.0013 (5)
C721	0.0216 (8)	0.0283 (10)	0.0315 (9)	−0.0059 (7)	0.0045 (7)	0.0013 (7)
C731	0.0190 (8)	0.0255 (9)	0.0282 (10)	−0.0069 (6)	0.0046 (7)	−0.0059 (7)
N731	0.0272 (8)	0.0400 (9)	0.0254 (8)	−0.0127 (7)	0.0020 (6)	−0.0002 (7)
C732	0.0221 (8)	0.0244 (9)	0.0263 (8)	0.0001 (7)	0.0019 (7)	−0.0016 (7)
N732	0.0285 (8)	0.0297 (8)	0.0370 (9)	−0.0055 (6)	0.0073 (7)	0.0009 (7)
C81	0.0179 (15)	0.0160 (15)	0.0189 (15)	−0.0005 (12)	0.0021 (12)	−0.0010 (12)
C82	0.010 (3)	0.015 (3)	0.015 (3)	0.001 (3)	0.003 (3)	−0.002 (3)
C83	0.0171 (15)	0.0154 (16)	0.0189 (15)	0.0002 (13)	0.0030 (13)	0.0020 (13)
C811	0.0203 (14)	0.003 (4)	0.028 (2)	0.001 (2)	0.0027 (15)	−0.004 (2)
N811	0.020 (5)	0.024 (4)	0.027 (3)	0.001 (3)	0.001 (3)	0.002 (2)
C812	0.018 (7)	0.0232 (11)	0.0216 (18)	−0.011 (3)	−0.004 (3)	−0.0005 (12)
N812	0.032 (2)	0.027 (2)	0.026 (2)	0.0033 (15)	0.0028 (16)	0.0046 (17)
O821	0.0211 (11)	0.0167 (11)	0.0238 (11)	0.0040 (9)	0.0052 (9)	0.0011 (9)
C821	0.026 (3)	0.018 (3)	0.020 (4)	0.005 (2)	0.005 (3)	−0.003 (3)
C831	0.011 (4)	0.020 (3)	0.024 (3)	0.001 (2)	−0.005 (3)	−0.005 (2)
N831	0.0245 (14)	0.0211 (15)	0.0232 (15)	−0.0033 (11)	0.0044 (11)	−0.0002 (12)
C832	0.0207 (10)	0.036 (3)	0.012 (3)	0.0038 (18)	0.0005 (18)	0.0045 (15)
N832	0.023 (3)	0.021 (3)	0.031 (4)	0.006 (2)	0.010 (3)	0.002 (2)
C91	0.0204 (15)	0.0266 (18)	0.0163 (15)	−0.0018 (13)	0.0026 (12)	0.0009 (13)
C92	0.014 (5)	0.018 (4)	0.019 (3)	0.000 (3)	−0.001 (3)	−0.003 (3)
C93	0.0211 (16)	0.0186 (16)	0.0194 (16)	−0.0024 (13)	−0.0002 (13)	0.0018 (13)
C911	0.0207 (10)	0.036 (3)	0.012 (3)	0.0038 (18)	0.0005 (18)	0.0045 (15)
N911	0.033 (5)	0.058 (5)	0.026 (4)	0.022 (3)	0.015 (3)	0.012 (3)
C912	0.018 (7)	0.0232 (11)	0.0216 (18)	−0.011 (3)	−0.004 (3)	−0.0005 (12)
N912	0.029 (2)	0.032 (2)	0.024 (2)	0.0027 (15)	0.0052 (15)	0.0040 (17)
O921	0.0230 (12)	0.0351 (14)	0.0186 (11)	−0.0015 (11)	0.0034 (10)	0.0065 (10)
C921	0.018 (5)	0.030 (3)	0.016 (3)	−0.006 (3)	0.001 (3)	0.002 (2)
C931	0.033 (3)	0.025 (4)	0.021 (4)	−0.002 (3)	0.003 (3)	0.002 (3)
N931	0.060 (2)	0.0267 (19)	0.0300 (17)	0.0072 (16)	0.0087 (16)	0.0065 (14)
C932	0.0203 (14)	0.003 (4)	0.028 (2)	0.001 (2)	0.0027 (15)	−0.004 (2)
N932	0.017 (4)	0.027 (5)	0.027 (3)	0.000 (3)	0.004 (4)	0.001 (2)
O101	0.0717 (16)	0.0340 (12)	0.0508 (14)	−0.0051 (10)	−0.0084 (10)	0.0019 (9)

### Tris(2,2'-bipyridine)iron(II) bis(1,1,3,3-tetracyano-2-methoxypropenide) 0.776-hydrate (I) Geometric parameters (Å, º)

**Table:**

Fe1—N31	1.9624 (13)	N61—C66	1.349 (2)
Fe1—N41	1.9677 (14)	N61—C62	1.355 (2)
Fe1—N61	1.9684 (13)	C62—C63	1.386 (2)
Fe1—N21	1.9700 (14)	C63—C64	1.383 (2)
Fe1—N11	1.9732 (14)	C63—H63	0.9500
Fe1—N51	1.9746 (14)	C64—C65	1.381 (3)
N11—C16	1.350 (2)	C64—H64	0.9500
N11—C12	1.360 (2)	C65—C66	1.379 (2)
C12—C13	1.386 (2)	C65—H65	0.9500
C12—C22	1.467 (2)	C66—H66	0.9500
C13—C14	1.378 (3)	C72—O721	1.350 (2)
C13—H13	0.9500	C72—C73	1.393 (2)
C14—C15	1.384 (3)	C72—C71	1.405 (3)
C14—H14	0.9500	C73—C731	1.421 (2)
C15—C16	1.380 (2)	C73—C732	1.426 (2)
C15—H15	0.9500	C71—C712	1.433 (4)
C16—H16	0.9500	C71—C711	1.443 (3)
N21—C26	1.345 (2)	C711—N711	1.150 (4)
N21—C22	1.360 (2)	C712—N712	1.147 (4)
C22—C23	1.387 (3)	C741—N741	1.155 (4)
C23—C24	1.380 (3)	C742—N742	1.150 (4)
C23—H23	0.9500	O721—C721	1.453 (2)
C24—C25	1.379 (3)	C721—H72A	0.9800
C24—H24	0.9500	C721—H72B	0.9800
C25—C26	1.381 (3)	C721—H72C	0.9800
C25—H25	0.9500	C731—N731	1.153 (2)
C26—H26	0.9500	C732—N732	1.151 (2)
N31—C36	1.345 (2)	C81—C82	1.383 (10)
N31—C32	1.359 (2)	C81—C811	1.412 (7)
C32—C33	1.387 (2)	C81—C812	1.448 (17)
C32—C42	1.468 (2)	C82—O821	1.318 (10)
C33—C34	1.384 (2)	C82—C83	1.45 (2)
C33—H33	0.9500	C83—C831	1.423 (11)
C34—C35	1.385 (2)	C83—C832	1.431 (6)
C34—H34	0.9500	C811—N811	1.165 (14)
C35—C36	1.381 (2)	C812—N812	1.136 (13)
C35—H35	0.9500	O821—C821	1.445 (8)
C36—H36	0.9500	C821—H82A	0.9800
N41—C46	1.349 (2)	C821—H82B	0.9800
N41—C42	1.361 (2)	C821—H82C	0.9800
C42—C43	1.387 (2)	C831—N831	1.158 (10)
C43—C44	1.388 (2)	C832—N832	1.139 (13)
C43—H43	0.9500	C91—C92	1.385 (10)
C44—C45	1.383 (3)	C91—C911	1.410 (7)
C44—H44	0.9500	C91—C912	1.446 (18)
C45—C46	1.379 (3)	C92—O921	1.322 (10)
C45—H45	0.9500	C92—C93	1.44 (2)
C46—H46	0.9500	C93—C931	1.422 (11)
N51—C56	1.352 (2)	C93—C932	1.435 (7)
N51—C52	1.356 (2)	C911—N911	1.166 (14)
C52—C53	1.389 (2)	C912—N912	1.136 (12)
C52—C62	1.474 (2)	O921—C921	1.449 (9)
C53—C54	1.384 (2)	C921—H92A	0.9800
C53—H53	0.9500	C921—H92B	0.9800
C54—C55	1.386 (3)	C921—H92C	0.9800
C54—H54	0.9500	C931—N931	1.156 (10)
C55—C56	1.378 (2)	C932—N932	1.137 (14)
C55—H55	0.9500	O101—H101	0.958 (10)
C56—H56	0.9500	O101—H102	0.953 (10)
			
N31—Fe1—N41	81.36 (5)	C53—C52—C62	123.73 (15)
N31—Fe1—N61	175.56 (6)	C54—C53—C52	119.03 (16)
N41—Fe1—N61	96.04 (6)	C54—C53—H53	120.5
N31—Fe1—N21	90.22 (5)	C52—C53—H53	120.5
N41—Fe1—N21	94.61 (6)	C53—C54—C55	118.81 (16)
N61—Fe1—N21	93.58 (6)	C53—C54—H54	120.6
N31—Fe1—N11	95.40 (5)	C55—C54—H54	120.6
N41—Fe1—N11	174.98 (6)	C56—C55—C54	119.56 (16)
N61—Fe1—N11	87.44 (5)	C56—C55—H55	120.2
N21—Fe1—N11	81.54 (6)	C54—C55—H55	120.2
N31—Fe1—N51	94.67 (5)	N51—C56—C55	122.35 (16)
N41—Fe1—N51	89.22 (6)	N51—C56—H56	118.8
N61—Fe1—N51	81.67 (5)	C55—C56—H56	118.8
N21—Fe1—N51	174.20 (6)	C66—N61—C62	117.72 (14)
N11—Fe1—N51	94.88 (5)	C66—N61—Fe1	126.87 (12)
C16—N11—C12	117.59 (15)	C62—N61—Fe1	115.19 (10)
C16—N11—Fe1	127.14 (12)	N61—C62—C63	122.27 (14)
C12—N11—Fe1	115.15 (11)	N61—C62—C52	113.93 (14)
N11—C12—C13	121.89 (16)	C63—C62—C52	123.79 (15)
N11—C12—C22	113.92 (15)	C64—C63—C62	119.17 (16)
C13—C12—C22	124.17 (16)	C64—C63—H63	120.4
C14—C13—C12	119.69 (17)	C62—C63—H63	120.4
C14—C13—H13	120.2	C65—C64—C63	118.84 (16)
C12—C13—H13	120.2	C65—C64—H64	120.6
C13—C14—C15	118.82 (17)	C63—C64—H64	120.6
C13—C14—H14	120.6	C66—C65—C64	119.31 (15)
C15—C14—H14	120.6	C66—C65—H65	120.3
C16—C15—C14	119.01 (16)	C64—C65—H65	120.3
C16—C15—H15	120.5	N61—C66—C65	122.68 (16)
C14—C15—H15	120.5	N61—C66—H66	118.7
N11—C16—C15	122.96 (16)	C65—C66—H66	118.7
N11—C16—H16	118.5	O721—C72—C73	114.29 (15)
C15—C16—H16	118.5	O721—C72—C71	121.76 (15)
C26—N21—C22	117.67 (15)	C73—C72—C71	123.95 (16)
C26—N21—Fe1	127.14 (13)	C72—C73—C731	121.06 (15)
C22—N21—Fe1	115.18 (11)	C72—C73—C732	122.33 (15)
N21—C22—C23	121.92 (17)	C731—C73—C732	116.59 (15)
N21—C22—C12	114.10 (14)	C72—C71—C712	117.0 (3)
C23—C22—C12	123.97 (17)	C72—C71—C711	128.0 (3)
C24—C23—C22	119.63 (19)	C712—C71—C711	114.1 (3)
C24—C23—H23	120.2	N711—C711—C71	173.7 (6)
C22—C23—H23	120.2	N712—C712—C71	178.6 (13)
C25—C24—C23	118.49 (18)	C72—O721—C721	118.13 (13)
C25—C24—H24	120.8	O721—C721—H72A	109.5
C23—C24—H24	120.8	O721—C721—H72B	109.5
C24—C25—C26	119.57 (18)	H72A—C721—H72B	109.5
C24—C25—H25	120.2	O721—C721—H72C	109.5
C26—C25—H25	120.2	H72A—C721—H72C	109.5
N21—C26—C25	122.72 (19)	H72B—C721—H72C	109.5
N21—C26—H26	118.6	N731—C731—C73	176.79 (18)
C25—C26—H26	118.6	N732—C732—C73	178.5 (2)
C36—N31—C32	118.09 (14)	C82—C81—C811	122.3 (9)
C36—N31—Fe1	126.23 (11)	C82—C81—C812	121.8 (14)
C32—N31—Fe1	115.68 (10)	C811—C81—C812	115.5 (11)
N31—C32—C33	121.79 (15)	O821—C82—C81	127.2 (16)
N31—C32—C42	113.78 (14)	O821—C82—C83	110.3 (8)
C33—C32—C42	124.41 (15)	C81—C82—C83	122.5 (9)
C34—C33—C32	119.68 (15)	C831—C83—C832	115.4 (5)
C34—C33—H33	120.2	C831—C83—C82	119.4 (5)
C32—C33—H33	120.2	C832—C83—C82	124.5 (3)
C33—C34—C35	118.35 (15)	N811—C811—C81	177.9 (11)
C33—C34—H34	120.8	N812—C812—C81	171 (3)
C35—C34—H34	120.8	C82—O821—C821	120.5 (9)
C36—C35—C34	119.51 (15)	O821—C821—H82A	109.5
C36—C35—H35	120.2	O821—C821—H82B	109.5
C34—C35—H35	120.2	H82A—C821—H82B	109.5
N31—C36—C35	122.57 (15)	O821—C821—H82C	109.5
N31—C36—H36	118.7	H82A—C821—H82C	109.5
C35—C36—H36	118.7	H82B—C821—H82C	109.5
C46—N41—C42	117.91 (14)	N831—C831—C83	177.7 (11)
C46—N41—Fe1	126.62 (12)	N832—C832—C83	178.2 (10)
C42—N41—Fe1	115.46 (11)	C92—C91—C911	121.3 (8)
N41—C42—C43	121.79 (15)	C92—C91—C912	122.2 (14)
N41—C42—C32	113.65 (14)	C911—C91—C912	116.5 (12)
C43—C42—C32	124.55 (15)	O921—C92—C91	125.8 (16)
C42—C43—C44	119.39 (16)	O921—C92—C93	111.1 (8)
C42—C43—H43	120.3	C91—C92—C93	123.0 (9)
C44—C43—H43	120.3	C931—C93—C932	115.0 (5)
C45—C44—C43	118.84 (16)	C931—C93—C92	120.0 (5)
C45—C44—H44	120.6	C932—C93—C92	124.9 (4)
C43—C44—H44	120.6	N911—C911—C91	177.4 (8)
C46—C45—C44	119.10 (16)	N912—C912—C91	170 (3)
C46—C45—H45	120.5	C92—O921—C921	119.0 (9)
C44—C45—H45	120.5	O921—C921—H92A	109.5
N41—C46—C45	122.95 (16)	O921—C921—H92B	109.5
N41—C46—H46	118.5	H92A—C921—H92B	109.5
C45—C46—H46	118.5	O921—C921—H92C	109.5
C56—N51—C52	117.97 (14)	H92A—C921—H92C	109.5
C56—N51—Fe1	127.03 (11)	H92B—C921—H92C	109.5
C52—N51—Fe1	114.95 (10)	N931—C931—C93	175.2 (9)
N51—C52—C53	122.28 (15)	N932—C932—C93	177.0 (9)
N51—C52—C62	113.99 (14)	H101—O101—H102	104.3 (14)
			
C16—N11—C12—C13	−1.2 (2)	C56—N51—C52—C62	178.47 (14)
Fe1—N11—C12—C13	175.16 (12)	Fe1—N51—C52—C62	−3.82 (17)
C16—N11—C12—C22	179.89 (13)	N51—C52—C53—C54	0.4 (2)
Fe1—N11—C12—C22	−3.77 (17)	C62—C52—C53—C54	−179.13 (16)
N11—C12—C13—C14	−0.4 (2)	C52—C53—C54—C55	0.4 (3)
C22—C12—C13—C14	178.40 (16)	C53—C54—C55—C56	−0.4 (3)
C12—C13—C14—C15	1.6 (3)	C52—N51—C56—C55	1.1 (2)
C13—C14—C15—C16	−1.3 (3)	Fe1—N51—C56—C55	−176.34 (13)
C12—N11—C16—C15	1.6 (2)	C54—C55—C56—N51	−0.3 (3)
Fe1—N11—C16—C15	−174.25 (12)	C66—N61—C62—C63	−1.3 (2)
C14—C15—C16—N11	−0.4 (3)	Fe1—N61—C62—C63	173.68 (12)
C26—N21—C22—C23	−0.9 (2)	C66—N61—C62—C52	179.89 (14)
Fe1—N21—C22—C23	−179.95 (13)	Fe1—N61—C62—C52	−5.13 (17)
C26—N21—C22—C12	178.22 (14)	N51—C52—C62—N61	5.9 (2)
Fe1—N21—C22—C12	−0.88 (17)	C53—C52—C62—N61	−174.61 (15)
N11—C12—C22—N21	3.0 (2)	N51—C52—C62—C63	−172.94 (15)
C13—C12—C22—N21	−175.86 (15)	C53—C52—C62—C63	6.6 (3)
N11—C12—C22—C23	−177.92 (16)	N61—C62—C63—C64	−0.1 (2)
C13—C12—C22—C23	3.2 (3)	C52—C62—C63—C64	178.60 (15)
N21—C22—C23—C24	0.7 (3)	C62—C63—C64—C65	1.2 (3)
C12—C22—C23—C24	−178.30 (17)	C63—C64—C65—C66	−1.0 (3)
C22—C23—C24—C25	0.2 (3)	C62—N61—C66—C65	1.6 (2)
C23—C24—C25—C26	−0.9 (3)	Fe1—N61—C66—C65	−172.72 (13)
C22—N21—C26—C25	0.1 (2)	C64—C65—C66—N61	−0.5 (3)
Fe1—N21—C26—C25	179.11 (14)	O721—C72—C73—C731	18.9 (2)
C24—C25—C26—N21	0.8 (3)	C71—C72—C73—C731	−161.99 (18)
C36—N31—C32—C33	0.3 (2)	O721—C72—C73—C732	−159.56 (16)
Fe1—N31—C32—C33	−179.51 (12)	C71—C72—C73—C732	19.5 (3)
C36—N31—C32—C42	178.94 (13)	O721—C72—C71—C712	−147.4 (4)
Fe1—N31—C32—C42	−0.92 (17)	C73—C72—C71—C712	33.6 (5)
N31—C32—C33—C34	0.1 (2)	O721—C72—C71—C711	21.3 (4)
C42—C32—C33—C34	−178.34 (15)	C73—C72—C71—C711	−157.7 (3)
C32—C33—C34—C35	−0.2 (2)	C73—C72—O721—C721	−145.54 (15)
C33—C34—C35—C36	−0.1 (2)	C71—C72—O721—C721	35.4 (2)
C32—N31—C36—C35	−0.7 (2)	C811—C81—C82—O821	−23.7 (18)
Fe1—N31—C36—C35	179.16 (12)	C812—C81—C82—O821	163.8 (16)
C34—C35—C36—N31	0.5 (2)	C811—C81—C82—C83	157.2 (11)
C46—N41—C42—C43	1.2 (2)	C812—C81—C82—C83	−15.3 (18)
Fe1—N41—C42—C43	−178.44 (12)	O821—C82—C83—C831	−6.2 (14)
C46—N41—C42—C32	−177.82 (14)	C81—C82—C83—C831	173.0 (10)
Fe1—N41—C42—C32	2.55 (17)	O821—C82—C83—C832	164.0 (7)
N31—C32—C42—N41	−1.07 (19)	C81—C82—C83—C832	−16.8 (16)
C33—C32—C42—N41	177.49 (15)	C81—C82—O821—C821	−17.4 (18)
N31—C32—C42—C43	179.96 (15)	C83—C82—O821—C821	161.8 (7)
C33—C32—C42—C43	−1.5 (3)	C911—C91—C92—O921	18.6 (18)
N41—C42—C43—C44	−1.5 (2)	C912—C91—C92—O921	−161.6 (14)
C32—C42—C43—C44	177.37 (15)	C911—C91—C92—C93	−158.8 (9)
C42—C43—C44—C45	0.6 (2)	C912—C91—C92—C93	20.9 (17)
C43—C44—C45—C46	0.5 (3)	O921—C92—C93—C931	14.6 (14)
C42—N41—C46—C45	0.0 (3)	C91—C92—C93—C931	−167.6 (10)
Fe1—N41—C46—C45	179.58 (14)	O921—C92—C93—C932	−162.3 (9)
C44—C45—C46—N41	−0.8 (3)	C91—C92—C93—C932	15.5 (17)
C56—N51—C52—C53	−1.1 (2)	C91—C92—O921—C921	26.6 (18)
Fe1—N51—C52—C53	176.63 (12)	C93—C92—O921—C921	−155.7 (10)

### Tris(2,2'-bipyridine)iron(II) bis(1,1,3,3-tetracyano-2-methoxypropenide) 0.776-hydrate (I) Hydrogen-bond geometry (Å, º)

**Table:**

*D*—H···*A*	*D*—H	H···*A*	*D*···*A*	*D*—H···*A*
C34—H34···N742	0.95	2.62	3.51 (3)	156
C43—H43···N741	0.95	2.59	3.525 (7)	170
C53—H53···N811	0.95	2.58	3.496 (15)	161
C63—H63···N811	0.95	2.47	3.329 (16)	151
C63—H63···N932	0.95	2.59	3.434 (16)	148
C66—H66···O101	0.95	2.49	3.297 (3)	142
C25—H25···N831^i^	0.95	2.48	3.398 (3)	162
C54—H54···N742^ii^	0.95	2.61	3.51 (3)	157
O101—H101···N812^iii^	0.96 (2)	2.23 (3)	3.143 (4)	159 (2)
O101—H101···N912^iii^	0.96 (2)	2.13 (3)	3.085 (5)	175 (3)
O101—H102···N832^iv^	0.95 (3)	2.13 (3)	3.017 (12)	154 (3)
O101—H102···N911^iv^	0.95 (3)	2.02 (3)	2.931 (14)	161 (3)

Symmetry codes: (i) −*x*+1, −*y*+1, −*z*+1; (ii) *x*, *y*+1, *z*; (iii) −*x*+1, *y*−1, −*z*+1/2; (iv) *x*, −*y*+2, *z*−1/2.

### Tris(2,2'-bipyridine)iron(II) 1,1,3,3-tetracyano-2-(propylsulfanyl)propenide perchlorate (II) Crystal data

**Table:**

[Fe(C_10_H_8_N_2_)_3_](C_10_H_7_N_4_S)(ClO_4_)	*F*(000) = 1728
*M**_r_* = 839.11	*D*_x_ = 1.489 Mg m^−^^3^
Monoclinic, *P*2_1_/*n*	Mo *K*α radiation, λ = 0.71073 Å
*a* = 11.6644 (3) Å	Cell parameters from 8586 reflections
*b* = 23.1692 (4) Å	θ = 1.7–28.3°
*c* = 13.9599 (3) Å	µ = 0.59 mm^−^^1^
β = 97.202 (2)°	*T* = 100 K
*V* = 3742.96 (14) Å^3^	Block, red
*Z* = 4	0.24 × 0.22 × 0.17 mm

### Tris(2,2'-bipyridine)iron(II) 1,1,3,3-tetracyano-2-(propylsulfanyl)propenide perchlorate (II) Data collection

**Table:**

SuperNova, Single source at offset, Eos diffractometer	8586 independent reflections
Radiation source: SuperNova (Mo) X-ray Source	5903 reflections with *I* > 2σ(*I*)
Mirror monochromator	*R*_int_ = 0.056
ω scans	θ_max_ = 28.3°, θ_min_ = 1.7°
Absorption correction: multi-scan (CrysAlis PRO; Rigaku OD, 2015)	*h* = −14→14
*T*_min_ = 0.724, *T*_max_ = 0.905	*k* = −30→25
30922 measured reflections	*l* = −16→18

### Tris(2,2'-bipyridine)iron(II) 1,1,3,3-tetracyano-2-(propylsulfanyl)propenide perchlorate (II) Refinement

**Table:**

Refinement on *F*^2^	151 restraints
Least-squares matrix: full	Hydrogen site location: inferred from neighbouring sites
*R*[*F*^2^ > 2σ(*F*^2^)] = 0.059	H-atom parameters constrained
*wR*(*F*^2^) = 0.170	*w* = 1/[σ^2^(*F*_o_^2^) + (0.0654*P*)^2^ + 4.7746*P*] where *P* = (*F*_o_^2^ + 2*F*_c_^2^)/3
*S* = 1.05	(Δ/σ)_max_ < 0.001
8586 reflections	Δρ_max_ = 2.23 e Å^−^^3^
721 parameters	Δρ_min_ = −0.42 e Å^−^^3^

### Tris(2,2'-bipyridine)iron(II) 1,1,3,3-tetracyano-2-(propylsulfanyl)propenide perchlorate (II) Special details

**Table:**

Geometry. All esds (except the esd in the dihedral angle between two l.s. planes) are estimated using the full covariance matrix. The cell esds are taken into account individually in the estimation of esds in distances, angles and torsion angles; correlations between esds in cell parameters are only used when they are defined by crystal symmetry. An approximate (isotropic) treatment of cell esds is used for estimating esds involving l.s. planes.

### Tris(2,2'-bipyridine)iron(II) 1,1,3,3-tetracyano-2-(propylsulfanyl)propenide perchlorate (II) Fractional atomic coordinates and isotropic or equivalent isotropic displacement parameters (Å^2^)

**Table:**

	*x*	*y*	*z*	*U*_iso_*/*U*_eq_	Occ. (<1)
Fe1	0.30977 (4)	0.32978 (2)	0.02224 (3)	0.01816 (14)	
N11	0.2100 (2)	0.39443 (11)	−0.02718 (18)	0.0238 (6)	
C12	0.2509 (3)	0.44802 (14)	−0.0004 (2)	0.0290 (8)	
C13	0.1904 (4)	0.49783 (16)	−0.0313 (3)	0.0446 (11)	
H13	0.2206	0.5348	−0.0125	0.054*	
C14	0.0865 (4)	0.4930 (2)	−0.0896 (3)	0.0516 (12)	
H14	0.0450	0.5266	−0.1124	0.062*	
C15	0.0438 (4)	0.4397 (2)	−0.1142 (3)	0.0484 (11)	
H15	−0.0288	0.4357	−0.1529	0.058*	
C16	0.1069 (3)	0.39120 (17)	−0.0823 (2)	0.0343 (8)	
H16	0.0762	0.3542	−0.1001	0.041*	
N21	0.4086 (2)	0.39372 (10)	0.07593 (17)	0.0210 (5)	
C22	0.3612 (3)	0.44721 (13)	0.0612 (2)	0.0272 (7)	
C23	0.4166 (4)	0.49601 (15)	0.1016 (3)	0.0422 (10)	
H23	0.3808	0.5328	0.0924	0.051*	
C24	0.5233 (4)	0.49113 (17)	0.1547 (3)	0.0470 (11)	
H24	0.5616	0.5242	0.1835	0.056*	
C25	0.5743 (4)	0.43723 (17)	0.1658 (3)	0.0415 (10)	
H25	0.6494	0.4329	0.2000	0.050*	
C26	0.5141 (3)	0.38968 (15)	0.1261 (2)	0.0291 (7)	
H26	0.5490	0.3527	0.1347	0.035*	
N31	0.2378 (2)	0.32277 (10)	0.14181 (18)	0.0223 (6)	
C32	0.2781 (3)	0.27876 (13)	0.2008 (2)	0.0267 (7)	
C33	0.2307 (4)	0.26647 (16)	0.2849 (2)	0.0365 (9)	
H33	0.2602	0.2355	0.3252	0.044*	
C34	0.1406 (3)	0.29947 (16)	0.3095 (3)	0.0378 (9)	
H34	0.1078	0.2917	0.3671	0.045*	
C35	0.0986 (3)	0.34391 (16)	0.2494 (3)	0.0356 (8)	
H35	0.0355	0.3667	0.2642	0.043*	
C36	0.1497 (3)	0.35459 (14)	0.1676 (2)	0.0290 (7)	
H36	0.1217	0.3859	0.1273	0.035*	
N41	0.4098 (2)	0.26850 (11)	0.08602 (18)	0.0247 (6)	
C42	0.3745 (3)	0.24706 (13)	0.1678 (2)	0.0282 (7)	
C43	0.4294 (4)	0.20011 (16)	0.2161 (3)	0.0451 (11)	
H43	0.4022	0.1849	0.2723	0.054*	
C44	0.5239 (5)	0.17604 (18)	0.1811 (3)	0.0567 (13)	
H44	0.5615	0.1435	0.2123	0.068*	
C45	0.5634 (4)	0.19931 (18)	0.1009 (3)	0.0551 (13)	
H45	0.6306	0.1844	0.0777	0.066*	
C46	0.5032 (3)	0.24502 (15)	0.0545 (3)	0.0362 (9)	
H46	0.5292	0.2604	−0.0021	0.043*	
N51	0.2070 (2)	0.27103 (11)	−0.04430 (19)	0.0247 (6)	
C52	0.2343 (3)	0.25462 (14)	−0.1326 (2)	0.0277 (7)	
C53	0.1713 (4)	0.21218 (16)	−0.1864 (3)	0.0411 (10)	
H53	0.1931	0.2003	−0.2467	0.049*	
C54	0.0771 (4)	0.18738 (19)	−0.1519 (3)	0.0505 (12)	
H54	0.0336	0.1582	−0.1879	0.061*	
C55	0.0475 (4)	0.20547 (18)	−0.0647 (3)	0.0484 (11)	
H55	−0.0184	0.1898	−0.0404	0.058*	
C56	0.1144 (3)	0.24667 (16)	−0.0128 (2)	0.0359 (9)	
H56	0.0939	0.2583	0.0480	0.043*	
N61	0.3799 (2)	0.32495 (10)	−0.09840 (18)	0.0209 (5)	
C62	0.3311 (3)	0.28605 (13)	−0.1643 (2)	0.0232 (7)	
C63	0.3711 (3)	0.27837 (15)	−0.2527 (2)	0.0309 (8)	
H63	0.3354	0.2509	−0.2974	0.037*	
C64	0.4633 (3)	0.31090 (15)	−0.2759 (3)	0.0335 (8)	
H64	0.4909	0.3067	−0.3367	0.040*	
C65	0.5142 (3)	0.34954 (16)	−0.2083 (3)	0.0359 (8)	
H65	0.5788	0.3719	−0.2214	0.043*	
C66	0.4704 (3)	0.35550 (14)	−0.1215 (2)	0.0288 (7)	
H66	0.5060	0.3825	−0.0758	0.035*	
C71	0.7798 (4)	0.42159 (18)	0.4708 (3)	0.0307 (10)	0.754 (2)
C72	0.7509 (4)	0.47479 (17)	0.4250 (3)	0.0240 (9)	0.754 (2)
C73	0.8200 (4)	0.50329 (17)	0.3655 (3)	0.0235 (9)	0.754 (2)
C711	0.7226 (5)	0.4012 (2)	0.5487 (4)	0.0394 (12)	0.754 (2)
N711	0.6791 (6)	0.3829 (3)	0.6125 (4)	0.0497 (17)	0.754 (2)
C712	0.8658 (6)	0.3846 (3)	0.4411 (6)	0.0412 (16)	0.754 (2)
N712	0.9299 (5)	0.35346 (19)	0.4133 (4)	0.0545 (14)	0.754 (2)
S721	0.61100 (10)	0.49930 (5)	0.43745 (9)	0.0301 (3)	0.754 (2)
C721	0.6244 (4)	0.57608 (19)	0.4640 (4)	0.0362 (11)	0.754 (2)
H71A	0.6552	0.5958	0.4097	0.043*	0.754 (2)
H71B	0.5467	0.5921	0.4691	0.043*	0.754 (2)
C722	0.7024 (5)	0.5888 (2)	0.5562 (4)	0.0410 (14)	0.754 (2)
H72A	0.7084	0.6312	0.5648	0.049*	0.754 (2)
H72B	0.7807	0.5739	0.5502	0.049*	0.754 (2)
C723	0.6607 (7)	0.5625 (3)	0.6455 (5)	0.067 (2)	0.754 (2)
H73A	0.6645	0.5204	0.6417	0.100*	0.754 (2)
H73B	0.5808	0.5745	0.6492	0.100*	0.754 (2)
H73C	0.7101	0.5759	0.7033	0.100*	0.754 (2)
C731	0.7746 (4)	0.54784 (18)	0.3018 (3)	0.0248 (9)	0.754 (2)
N731	0.7414 (4)	0.58433 (17)	0.2496 (3)	0.0359 (10)	0.754 (2)
C732	0.9400 (5)	0.4924 (2)	0.3637 (4)	0.0307 (12)	0.754 (2)
N732	1.0356 (4)	0.4860 (2)	0.3599 (4)	0.0463 (12)	0.754 (2)
C81	0.7049 (11)	0.5420 (3)	0.5119 (6)	0.031 (3)	0.246 (2)
C82	0.7168 (10)	0.4822 (3)	0.5283 (5)	0.026 (2)	0.246 (2)
C83	0.7263 (11)	0.4558 (3)	0.6187 (5)	0.030 (3)	0.246 (2)
C811	0.7222 (12)	0.5672 (5)	0.4215 (8)	0.038 (3)	0.246 (2)
N811	0.7388 (13)	0.5885 (6)	0.3494 (8)	0.053 (4)	0.246 (2)
C812	0.6719 (17)	0.5803 (6)	0.5832 (11)	0.038 (3)	0.246 (2)
N812	0.6464 (11)	0.6069 (7)	0.6460 (8)	0.052 (3)	0.246 (2)
S821	0.7045 (3)	0.43981 (16)	0.4227 (2)	0.0315 (10)	0.246 (2)
C821	0.8319 (8)	0.3935 (5)	0.4354 (13)	0.025 (4)	0.246 (2)
H82A	0.8329	0.3704	0.4951	0.030*	0.246 (2)
H82B	0.8263	0.3664	0.3802	0.030*	0.246 (2)
C822	0.9441 (8)	0.4265 (7)	0.4395 (11)	0.054 (4)	0.246 (2)
H82C	1.0089	0.3986	0.4458	0.065*	0.246 (2)
H82D	0.9528	0.4510	0.4980	0.065*	0.246 (2)
C823	0.9526 (19)	0.4644 (9)	0.3518 (15)	0.062 (6)	0.246 (2)
H82E	0.9363	0.4413	0.2930	0.092*	0.246 (2)
H82F	1.0307	0.4805	0.3554	0.092*	0.246 (2)
H82G	0.8964	0.4959	0.3506	0.092*	0.246 (2)
C831	0.712 (2)	0.3949 (4)	0.6254 (12)	0.034 (5)	0.246 (2)
N831	0.6866 (12)	0.3475 (4)	0.6352 (9)	0.037 (3)	0.246 (2)
C832	0.7683 (12)	0.4819 (6)	0.7086 (6)	0.038 (3)	0.246 (2)
N832	0.8066 (10)	0.4990 (7)	0.7808 (7)	0.052 (3)	0.246 (2)
Cl91	0.3052 (6)	0.1693 (3)	0.5253 (8)	0.0257 (3)	0.439 (3)
O1	0.3079 (11)	0.2311 (3)	0.5216 (8)	0.039 (3)	0.439 (3)
O2	0.2135 (8)	0.1478 (4)	0.4583 (7)	0.065 (3)	0.439 (3)
O3	0.2832 (10)	0.1515 (3)	0.6211 (5)	0.055 (2)	0.439 (3)
O4	0.4106 (6)	0.1451 (4)	0.5063 (8)	0.067 (3)	0.439 (3)
Cl92	0.3087 (7)	0.1656 (3)	0.5265 (8)	0.0257 (3)	0.377 (3)
O5	0.2209 (6)	0.2042 (3)	0.5509 (6)	0.049 (2)	0.377 (3)
O6	0.3995 (6)	0.1976 (3)	0.4927 (6)	0.053 (2)	0.377 (3)
O7	0.2590 (10)	0.1281 (4)	0.4488 (8)	0.049 (3)	0.377 (3)
O8	0.3521 (9)	0.1314 (4)	0.6059 (6)	0.055 (3)	0.377 (3)
Cl93	0.3061 (12)	0.1701 (8)	0.5163 (9)	0.0257 (3)	0.184 (3)
O9	0.4143 (13)	0.1588 (10)	0.5739 (14)	0.065 (6)	0.184 (3)
O10	0.310 (2)	0.1504 (8)	0.4204 (9)	0.070 (8)	0.184 (3)
O11	0.287 (2)	0.2321 (6)	0.5125 (18)	0.071 (15)	0.184 (3)
O12	0.2142 (13)	0.1437 (9)	0.5554 (18)	0.092 (9)	0.184 (3)

### Tris(2,2'-bipyridine)iron(II) 1,1,3,3-tetracyano-2-(propylsulfanyl)propenide perchlorate (II) Atomic displacement parameters (Å^2^)

**Table:**

	*U*^11^	*U*^22^	*U*^33^	*U*^12^	*U*^13^	*U*^23^
Fe1	0.0182 (2)	0.0193 (2)	0.0174 (2)	−0.00055 (16)	0.00370 (17)	−0.00130 (16)
N11	0.0246 (15)	0.0298 (14)	0.0189 (13)	0.0039 (11)	0.0095 (11)	0.0023 (11)
C12	0.036 (2)	0.0251 (16)	0.0304 (18)	0.0082 (14)	0.0203 (15)	0.0055 (13)
C13	0.062 (3)	0.0296 (19)	0.048 (2)	0.0146 (18)	0.029 (2)	0.0114 (17)
C14	0.053 (3)	0.059 (3)	0.047 (2)	0.039 (2)	0.022 (2)	0.022 (2)
C15	0.039 (2)	0.074 (3)	0.034 (2)	0.031 (2)	0.0105 (17)	0.012 (2)
C16	0.0261 (19)	0.051 (2)	0.0260 (18)	0.0113 (16)	0.0049 (14)	−0.0009 (16)
N21	0.0239 (15)	0.0230 (13)	0.0171 (13)	−0.0033 (10)	0.0069 (10)	−0.0019 (10)
C22	0.036 (2)	0.0201 (15)	0.0289 (18)	−0.0030 (13)	0.0165 (15)	−0.0002 (13)
C23	0.052 (3)	0.0267 (18)	0.052 (2)	−0.0111 (16)	0.024 (2)	−0.0096 (17)
C24	0.061 (3)	0.039 (2)	0.043 (2)	−0.026 (2)	0.014 (2)	−0.0185 (18)
C25	0.044 (2)	0.053 (2)	0.0268 (19)	−0.0232 (19)	0.0014 (16)	−0.0066 (17)
C26	0.032 (2)	0.0331 (18)	0.0222 (16)	−0.0049 (14)	0.0023 (14)	−0.0016 (13)
N31	0.0231 (14)	0.0224 (13)	0.0215 (13)	−0.0024 (10)	0.0028 (11)	−0.0014 (10)
C32	0.0307 (19)	0.0266 (16)	0.0218 (16)	−0.0043 (13)	−0.0004 (13)	−0.0021 (13)
C33	0.048 (2)	0.038 (2)	0.0240 (18)	−0.0105 (17)	0.0063 (16)	0.0064 (15)
C34	0.042 (2)	0.050 (2)	0.0232 (18)	−0.0116 (18)	0.0135 (16)	0.0028 (16)
C35	0.031 (2)	0.048 (2)	0.0302 (19)	−0.0005 (16)	0.0163 (15)	−0.0031 (16)
C36	0.0289 (19)	0.0327 (17)	0.0270 (17)	−0.0002 (14)	0.0094 (14)	−0.0002 (14)
N41	0.0293 (16)	0.0243 (13)	0.0199 (13)	0.0045 (11)	0.0009 (11)	−0.0055 (10)
C42	0.036 (2)	0.0261 (16)	0.0218 (16)	0.0010 (14)	−0.0015 (14)	−0.0005 (13)
C43	0.069 (3)	0.035 (2)	0.029 (2)	0.0148 (19)	−0.0020 (19)	0.0089 (16)
C44	0.083 (4)	0.047 (2)	0.037 (2)	0.036 (2)	−0.005 (2)	0.0017 (19)
C45	0.070 (3)	0.056 (3)	0.038 (2)	0.039 (2)	0.001 (2)	−0.009 (2)
C46	0.039 (2)	0.040 (2)	0.0294 (19)	0.0144 (16)	0.0041 (16)	−0.0036 (15)
N51	0.0258 (15)	0.0267 (14)	0.0215 (13)	−0.0051 (11)	0.0028 (11)	−0.0002 (11)
C52	0.034 (2)	0.0289 (17)	0.0204 (16)	−0.0024 (14)	0.0021 (14)	−0.0042 (13)
C53	0.048 (3)	0.047 (2)	0.0286 (19)	−0.0193 (18)	0.0059 (17)	−0.0107 (17)
C54	0.062 (3)	0.059 (3)	0.031 (2)	−0.037 (2)	0.0063 (19)	−0.0138 (19)
C55	0.052 (3)	0.062 (3)	0.032 (2)	−0.038 (2)	0.0083 (18)	−0.0059 (18)
C56	0.042 (2)	0.045 (2)	0.0210 (17)	−0.0195 (17)	0.0046 (15)	−0.0033 (15)
N61	0.0201 (14)	0.0222 (13)	0.0208 (13)	0.0018 (10)	0.0034 (10)	−0.0022 (10)
C62	0.0243 (17)	0.0230 (15)	0.0227 (16)	0.0030 (12)	0.0038 (13)	−0.0032 (12)
C63	0.032 (2)	0.0345 (18)	0.0268 (18)	0.0063 (14)	0.0054 (14)	−0.0088 (14)
C64	0.035 (2)	0.0403 (19)	0.0280 (18)	0.0047 (15)	0.0133 (15)	−0.0059 (15)
C65	0.031 (2)	0.045 (2)	0.034 (2)	−0.0049 (16)	0.0156 (16)	−0.0064 (16)
C66	0.0246 (18)	0.0341 (18)	0.0295 (18)	−0.0049 (14)	0.0102 (14)	−0.0078 (14)
C71	0.035 (3)	0.026 (2)	0.035 (3)	0.0025 (19)	0.019 (2)	0.0028 (19)
C72	0.028 (2)	0.017 (2)	0.028 (2)	−0.0057 (18)	0.0102 (18)	−0.0081 (17)
C73	0.024 (2)	0.023 (2)	0.024 (2)	−0.0009 (16)	0.0042 (17)	0.0008 (16)
C711	0.047 (3)	0.028 (2)	0.047 (3)	0.010 (2)	0.019 (3)	0.010 (2)
N711	0.051 (5)	0.052 (3)	0.054 (4)	0.005 (3)	0.035 (3)	0.021 (3)
C712	0.046 (4)	0.035 (3)	0.048 (4)	0.010 (3)	0.022 (3)	0.020 (3)
N712	0.068 (4)	0.042 (2)	0.063 (3)	0.026 (2)	0.046 (3)	0.024 (2)
S721	0.0297 (6)	0.0273 (6)	0.0362 (7)	0.0003 (4)	0.0155 (5)	−0.0045 (5)
C721	0.040 (3)	0.026 (2)	0.043 (3)	0.007 (2)	0.009 (2)	−0.006 (2)
C722	0.038 (4)	0.034 (3)	0.053 (4)	0.001 (2)	0.011 (3)	−0.013 (3)
C723	0.078 (5)	0.071 (4)	0.055 (4)	−0.013 (4)	0.021 (4)	−0.041 (4)
C731	0.024 (2)	0.027 (2)	0.024 (2)	0.0001 (17)	0.0065 (17)	0.0002 (18)
N731	0.034 (2)	0.038 (2)	0.036 (2)	0.0019 (18)	0.0099 (18)	0.0086 (18)
C732	0.036 (3)	0.026 (3)	0.031 (3)	0.000 (2)	0.006 (2)	0.008 (2)
N732	0.026 (3)	0.051 (3)	0.063 (3)	0.002 (2)	0.008 (2)	0.015 (2)
C81	0.021 (7)	0.039 (5)	0.033 (5)	−0.003 (4)	0.000 (4)	0.001 (3)
C82	0.011 (6)	0.036 (4)	0.032 (4)	0.003 (4)	0.002 (4)	−0.002 (3)
C83	0.026 (7)	0.031 (5)	0.033 (4)	0.002 (4)	0.006 (4)	0.004 (3)
C811	0.020 (7)	0.045 (7)	0.048 (6)	0.006 (6)	0.002 (5)	0.015 (5)
N811	0.055 (10)	0.049 (8)	0.057 (7)	0.009 (7)	0.012 (6)	0.023 (6)
C812	0.023 (5)	0.054 (5)	0.038 (4)	−0.006 (4)	0.011 (3)	−0.006 (4)
N812	0.016 (4)	0.107 (8)	0.031 (4)	0.012 (5)	0.000 (3)	−0.015 (4)
S821	0.033 (2)	0.034 (2)	0.0282 (19)	−0.0003 (16)	0.0059 (15)	−0.0042 (15)
C821	0.024 (6)	0.022 (7)	0.029 (8)	0.004 (5)	0.003 (5)	0.007 (6)
C822	0.040 (7)	0.053 (9)	0.071 (10)	−0.008 (6)	0.014 (6)	0.001 (7)
C823	0.056 (13)	0.052 (11)	0.082 (13)	−0.003 (11)	0.030 (10)	0.014 (10)
C831	0.022 (10)	0.037 (5)	0.044 (10)	0.002 (4)	0.003 (7)	0.007 (4)
N831	0.046 (8)	0.036 (5)	0.031 (7)	0.000 (5)	0.016 (6)	0.006 (5)
C832	0.023 (5)	0.054 (5)	0.038 (4)	−0.006 (4)	0.011 (3)	−0.006 (4)
N832	0.016 (4)	0.107 (8)	0.031 (4)	0.012 (5)	0.000 (3)	−0.015 (4)
Cl91	0.0208 (5)	0.0310 (7)	0.0254 (7)	0.0002 (4)	0.0034 (5)	−0.0002 (5)
O1	0.061 (6)	0.026 (6)	0.032 (6)	−0.008 (4)	0.021 (4)	0.005 (4)
O2	0.058 (7)	0.083 (8)	0.045 (5)	−0.023 (5)	−0.024 (5)	−0.001 (5)
O3	0.083 (8)	0.048 (5)	0.038 (4)	−0.018 (5)	0.024 (5)	0.002 (4)
O4	0.030 (4)	0.063 (5)	0.114 (8)	0.008 (3)	0.039 (5)	−0.012 (5)
Cl92	0.0208 (5)	0.0310 (7)	0.0254 (7)	0.0002 (4)	0.0034 (5)	−0.0002 (5)
O5	0.030 (4)	0.056 (5)	0.062 (6)	0.002 (3)	0.016 (4)	−0.020 (4)
O6	0.030 (4)	0.059 (5)	0.076 (6)	−0.009 (4)	0.020 (4)	0.023 (4)
O7	0.060 (8)	0.040 (6)	0.040 (6)	0.013 (5)	−0.017 (5)	−0.010 (4)
O8	0.074 (8)	0.046 (5)	0.037 (5)	−0.019 (5)	−0.028 (5)	0.022 (4)
Cl93	0.0208 (5)	0.0310 (7)	0.0254 (7)	0.0002 (4)	0.0034 (5)	−0.0002 (5)
O9	0.027 (10)	0.089 (17)	0.074 (15)	0.013 (10)	−0.011 (10)	−0.003 (13)
O10	0.14 (3)	0.034 (10)	0.025 (9)	0.032 (12)	−0.018 (11)	−0.004 (8)
O11	0.12 (3)	0.04 (2)	0.05 (2)	0.027 (18)	0.020 (19)	−0.015 (14)
O12	0.025 (11)	0.087 (16)	0.18 (3)	0.000 (10)	0.057 (14)	0.050 (17)

### Tris(2,2'-bipyridine)iron(II) 1,1,3,3-tetracyano-2-(propylsulfanyl)propenide perchlorate (II) Geometric parameters (Å, º)

**Table:**

Fe1—N61	1.965 (3)	C63—C64	1.384 (5)
Fe1—N31	1.967 (3)	C63—H63	0.9500
Fe1—N21	1.967 (2)	C64—C65	1.380 (5)
Fe1—N11	1.968 (3)	C64—H64	0.9500
Fe1—N51	1.968 (3)	C65—C66	1.380 (5)
Fe1—N41	1.977 (3)	C65—H65	0.9500
N11—C16	1.347 (4)	C66—H66	0.9500
N11—C12	1.365 (4)	C71—C72	1.410 (6)
C12—C13	1.393 (5)	C71—C712	1.419 (7)
C12—C22	1.455 (5)	C71—C711	1.427 (6)
C13—C14	1.377 (6)	C72—C73	1.394 (6)
C13—H13	0.9500	C72—S721	1.757 (4)
C14—C15	1.360 (6)	C73—C731	1.420 (5)
C14—H14	0.9500	C73—C732	1.426 (7)
C15—C16	1.386 (5)	C711—N711	1.159 (7)
C15—H15	0.9500	C712—N712	1.142 (8)
C16—H16	0.9500	S721—C721	1.820 (4)
N21—C26	1.341 (4)	C721—C722	1.509 (8)
N21—C22	1.362 (4)	C721—H71A	0.9900
C22—C23	1.386 (5)	C721—H71B	0.9900
C23—C24	1.372 (6)	C722—C723	1.522 (8)
C23—H23	0.9500	C722—H72A	0.9900
C24—C25	1.383 (6)	C722—H72B	0.9900
C24—H24	0.9500	C723—H73A	0.9800
C25—C26	1.384 (5)	C723—H73B	0.9800
C25—H25	0.9500	C723—H73C	0.9800
C26—H26	0.9500	C731—N731	1.151 (5)
N31—C36	1.350 (4)	C732—N732	1.132 (7)
N31—C32	1.357 (4)	C81—C82	1.409 (7)
C32—C33	1.389 (5)	C81—C812	1.421 (8)
C32—C42	1.464 (5)	C81—C811	1.427 (8)
C33—C34	1.378 (5)	C82—C83	1.395 (7)
C33—H33	0.9500	C82—S821	1.762 (6)
C34—C35	1.379 (5)	C83—C831	1.424 (7)
C34—H34	0.9500	C83—C832	1.424 (8)
C35—C36	1.375 (5)	C811—N811	1.160 (8)
C35—H35	0.9500	C812—N812	1.140 (9)
C36—H36	0.9500	S821—C821	1.823 (6)
N41—C46	1.340 (4)	C821—C822	1.509 (9)
N41—C42	1.355 (4)	C821—H82A	0.9900
C42—C43	1.393 (5)	C821—H82B	0.9900
C43—C44	1.378 (6)	C822—C823	1.520 (9)
C43—H43	0.9500	C822—H82C	0.9900
C44—C45	1.372 (6)	C822—H82D	0.9900
C44—H44	0.9500	C823—H82E	0.9800
C45—C46	1.386 (5)	C823—H82F	0.9800
C45—H45	0.9500	C823—H82G	0.9800
C46—H46	0.9500	C831—N831	1.151 (7)
N51—C56	1.340 (4)	C832—N832	1.121 (8)
N51—C52	1.365 (4)	Cl91—O4	1.405 (8)
C52—C53	1.390 (5)	Cl91—O2	1.420 (7)
C52—C62	1.459 (5)	Cl91—O1	1.434 (7)
C53—C54	1.379 (5)	Cl91—O3	1.453 (11)
C53—H53	0.9500	Cl92—O8	1.405 (8)
C54—C55	1.372 (6)	Cl92—O6	1.421 (8)
C54—H54	0.9500	Cl92—O5	1.433 (7)
C55—C56	1.379 (5)	Cl92—O7	1.452 (12)
C55—H55	0.9500	Cl93—O12	1.404 (9)
C56—H56	0.9500	Cl93—O10	1.421 (8)
N61—C66	1.344 (4)	Cl93—O9	1.433 (7)
N61—C62	1.360 (4)	Cl93—O11	1.453 (12)
C62—C63	1.385 (5)		
			
N61—Fe1—N31	171.97 (10)	N51—C56—H56	118.5
N61—Fe1—N21	94.74 (10)	C55—C56—H56	118.5
N31—Fe1—N21	91.77 (10)	C66—N61—C62	117.5 (3)
N61—Fe1—N11	91.95 (10)	C66—N61—Fe1	126.8 (2)
N31—Fe1—N11	93.66 (11)	C62—N61—Fe1	115.7 (2)
N21—Fe1—N11	81.52 (11)	N61—C62—C63	121.9 (3)
N61—Fe1—N51	81.40 (11)	N61—C62—C52	113.5 (3)
N31—Fe1—N51	92.51 (11)	C63—C62—C52	124.5 (3)
N21—Fe1—N51	173.70 (11)	C64—C63—C62	119.8 (3)
N11—Fe1—N51	93.59 (11)	C64—C63—H63	120.1
N61—Fe1—N41	93.42 (11)	C62—C63—H63	120.1
N31—Fe1—N41	81.32 (11)	C65—C64—C63	118.3 (3)
N21—Fe1—N41	94.87 (11)	C65—C64—H64	120.9
N11—Fe1—N41	173.76 (10)	C63—C64—H64	120.9
N51—Fe1—N41	90.35 (11)	C66—C65—C64	119.4 (3)
C16—N11—C12	117.6 (3)	C66—C65—H65	120.3
C16—N11—Fe1	127.2 (2)	C64—C65—H65	120.3
C12—N11—Fe1	115.2 (2)	N61—C66—C65	123.1 (3)
N11—C12—C13	121.5 (4)	N61—C66—H66	118.4
N11—C12—C22	113.8 (3)	C65—C66—H66	118.4
C13—C12—C22	124.8 (3)	C72—C71—C712	122.2 (4)
C14—C13—C12	119.4 (4)	C72—C71—C711	121.8 (4)
C14—C13—H13	120.3	C712—C71—C711	115.9 (4)
C12—C13—H13	120.3	C73—C72—C71	124.1 (4)
C15—C14—C13	119.3 (4)	C73—C72—S721	121.3 (3)
C15—C14—H14	120.3	C71—C72—S721	114.2 (3)
C13—C14—H14	120.3	C72—C73—C731	121.2 (4)
C14—C15—C16	119.5 (4)	C72—C73—C732	124.7 (4)
C14—C15—H15	120.2	C731—C73—C732	114.1 (4)
C16—C15—H15	120.2	N711—C711—C71	177.5 (6)
N11—C16—C15	122.6 (4)	N712—C712—C71	175.8 (8)
N11—C16—H16	118.7	C72—S721—C721	106.2 (2)
C15—C16—H16	118.7	C722—C721—S721	113.1 (3)
C26—N21—C22	118.1 (3)	C722—C721—H71A	109.0
C26—N21—Fe1	127.0 (2)	S721—C721—H71A	109.0
C22—N21—Fe1	114.9 (2)	C722—C721—H71B	109.0
N21—C22—C23	121.4 (3)	S721—C721—H71B	109.0
N21—C22—C12	114.4 (3)	H71A—C721—H71B	107.8
C23—C22—C12	124.2 (3)	C721—C722—C723	113.6 (5)
C24—C23—C22	119.8 (4)	C721—C722—H72A	108.9
C24—C23—H23	120.1	C723—C722—H72A	108.9
C22—C23—H23	120.1	C721—C722—H72B	108.9
C23—C24—C25	118.9 (3)	C723—C722—H72B	108.9
C23—C24—H24	120.6	H72A—C722—H72B	107.7
C25—C24—H24	120.6	C722—C723—H73A	109.5
C24—C25—C26	119.0 (4)	C722—C723—H73B	109.5
C24—C25—H25	120.5	H73A—C723—H73B	109.5
C26—C25—H25	120.5	C722—C723—H73C	109.5
N21—C26—C25	122.7 (3)	H73A—C723—H73C	109.5
N21—C26—H26	118.7	H73B—C723—H73C	109.5
C25—C26—H26	118.7	N731—C731—C73	177.8 (5)
C36—N31—C32	117.8 (3)	N732—C732—C73	176.8 (6)
C36—N31—Fe1	127.0 (2)	C82—C81—C812	122.0 (7)
C32—N31—Fe1	115.1 (2)	C82—C81—C811	121.6 (7)
N31—C32—C33	121.5 (3)	C812—C81—C811	116.4 (7)
N31—C32—C42	114.0 (3)	C83—C82—C81	125.0 (6)
C33—C32—C42	124.5 (3)	C83—C82—S821	120.0 (5)
C34—C33—C32	119.7 (3)	C81—C82—S821	114.7 (5)
C34—C33—H33	120.2	C82—C83—C831	119.8 (7)
C32—C33—H33	120.2	C82—C83—C832	126.2 (7)
C33—C34—C35	119.1 (3)	C831—C83—C832	113.2 (7)
C33—C34—H34	120.5	N811—C811—C81	178.1 (17)
C35—C34—H34	120.5	N812—C812—C81	173.8 (17)
C36—C35—C34	118.8 (4)	C82—S821—C821	105.5 (5)
C36—C35—H35	120.6	C822—C821—S821	113.5 (7)
C34—C35—H35	120.6	C822—C821—H82A	108.9
N31—C36—C35	123.2 (3)	S821—C821—H82A	108.9
N31—C36—H36	118.4	C822—C821—H82B	108.9
C35—C36—H36	118.4	S821—C821—H82B	108.9
C46—N41—C42	118.2 (3)	H82A—C821—H82B	107.7
C46—N41—Fe1	127.0 (2)	C821—C822—C823	113.9 (8)
C42—N41—Fe1	114.7 (2)	C821—C822—H82C	108.8
N41—C42—C43	121.6 (3)	C823—C822—H82C	108.8
N41—C42—C32	114.0 (3)	C821—C822—H82D	108.8
C43—C42—C32	124.3 (3)	C823—C822—H82D	108.8
C44—C43—C42	118.9 (4)	H82C—C822—H82D	107.7
C44—C43—H43	120.5	C822—C823—H82E	109.5
C42—C43—H43	120.5	C822—C823—H82F	109.5
C45—C44—C43	119.7 (4)	H82E—C823—H82F	109.5
C45—C44—H44	120.2	C822—C823—H82G	109.5
C43—C44—H44	120.2	H82E—C823—H82G	109.5
C44—C45—C46	118.6 (4)	H82F—C823—H82G	109.5
C44—C45—H45	120.7	N831—C831—C83	171 (3)
C46—C45—H45	120.7	N832—C832—C83	174.8 (16)
N41—C46—C45	122.8 (4)	O4—Cl91—O2	110.0 (8)
N41—C46—H46	118.6	O4—Cl91—O1	111.6 (7)
C45—C46—H46	118.6	O2—Cl91—O1	110.2 (7)
C56—N51—C52	117.9 (3)	O4—Cl91—O3	108.7 (6)
C56—N51—Fe1	127.2 (2)	O2—Cl91—O3	107.5 (8)
C52—N51—Fe1	114.9 (2)	O1—Cl91—O3	108.8 (6)
N51—C52—C53	121.2 (3)	O8—Cl92—O6	110.3 (8)
N51—C52—C62	114.3 (3)	O8—Cl92—O5	111.2 (8)
C53—C52—C62	124.5 (3)	O6—Cl92—O5	109.7 (6)
C54—C53—C52	119.7 (4)	O8—Cl92—O7	108.8 (7)
C54—C53—H53	120.1	O6—Cl92—O7	108.0 (9)
C52—C53—H53	120.1	O5—Cl92—O7	108.7 (7)
C55—C54—C53	118.9 (3)	O12—Cl93—O10	110.0 (9)
C55—C54—H54	120.6	O12—Cl93—O9	111.3 (9)
C53—C54—H54	120.6	O10—Cl93—O9	109.8 (8)
C54—C55—C56	119.2 (4)	O12—Cl93—O11	108.9 (8)
C54—C55—H55	120.4	O10—Cl93—O11	107.9 (9)
C56—C55—H55	120.4	O9—Cl93—O11	108.8 (8)
N51—C56—C55	123.1 (3)		
			
C16—N11—C12—C13	−2.1 (5)	C44—C45—C46—N41	1.8 (6)
Fe1—N11—C12—C13	178.9 (3)	C56—N51—C52—C53	−2.8 (5)
C16—N11—C12—C22	178.4 (3)	Fe1—N51—C52—C53	177.9 (3)
Fe1—N11—C12—C22	−0.6 (4)	C56—N51—C52—C62	175.9 (3)
N11—C12—C13—C14	0.6 (5)	Fe1—N51—C52—C62	−3.3 (4)
C22—C12—C13—C14	−179.9 (3)	N51—C52—C53—C54	2.2 (6)
C12—C13—C14—C15	1.3 (6)	C62—C52—C53—C54	−176.4 (4)
C13—C14—C15—C16	−1.7 (6)	C52—C53—C54—C55	0.2 (7)
C12—N11—C16—C15	1.7 (5)	C53—C54—C55—C56	−1.8 (7)
Fe1—N11—C16—C15	−179.4 (3)	C52—N51—C56—C55	1.1 (6)
C14—C15—C16—N11	0.1 (6)	Fe1—N51—C56—C55	−179.8 (3)
C26—N21—C22—C23	−3.9 (5)	C54—C55—C56—N51	1.2 (7)
Fe1—N21—C22—C23	174.7 (3)	C66—N61—C62—C63	−1.0 (4)
C26—N21—C22—C12	175.2 (3)	Fe1—N61—C62—C63	179.5 (2)
Fe1—N21—C22—C12	−6.2 (4)	C66—N61—C62—C52	179.5 (3)
N11—C12—C22—N21	4.4 (4)	Fe1—N61—C62—C52	0.0 (3)
C13—C12—C22—N21	−175.1 (3)	N51—C52—C62—N61	2.2 (4)
N11—C12—C22—C23	−176.5 (3)	C53—C52—C62—N61	−179.1 (3)
C13—C12—C22—C23	4.0 (6)	N51—C52—C62—C63	−177.3 (3)
N21—C22—C23—C24	2.3 (6)	C53—C52—C62—C63	1.4 (6)
C12—C22—C23—C24	−176.7 (3)	N61—C62—C63—C64	0.0 (5)
C22—C23—C24—C25	1.0 (6)	C52—C62—C63—C64	179.5 (3)
C23—C24—C25—C26	−2.5 (6)	C62—C63—C64—C65	1.2 (5)
C22—N21—C26—C25	2.2 (5)	C63—C64—C65—C66	−1.5 (5)
Fe1—N21—C26—C25	−176.2 (3)	C62—N61—C66—C65	0.8 (5)
C24—C25—C26—N21	1.0 (6)	Fe1—N61—C66—C65	−179.8 (3)
C36—N31—C32—C33	0.2 (4)	C64—C65—C66—N61	0.4 (6)
Fe1—N31—C32—C33	−176.1 (2)	C712—C71—C72—C73	−17.2 (8)
C36—N31—C32—C42	−179.3 (3)	C711—C71—C72—C73	164.0 (5)
Fe1—N31—C32—C42	4.4 (3)	C712—C71—C72—S721	155.9 (5)
N31—C32—C33—C34	0.0 (5)	C711—C71—C72—S721	−22.9 (6)
C42—C32—C33—C34	179.4 (3)	C71—C72—C73—C731	163.1 (4)
C32—C33—C34—C35	0.6 (5)	S721—C72—C73—C731	−9.5 (6)
C33—C34—C35—C36	−1.3 (5)	C71—C72—C73—C732	−18.5 (7)
C32—N31—C36—C35	−1.0 (5)	S721—C72—C73—C732	168.9 (4)
Fe1—N31—C36—C35	174.8 (3)	C73—C72—S721—C721	−50.0 (4)
C34—C35—C36—N31	1.5 (5)	C71—C72—S721—C721	136.8 (3)
C46—N41—C42—C43	−3.0 (5)	C72—S721—C721—C722	−60.9 (4)
Fe1—N41—C42—C43	174.0 (3)	S721—C721—C722—C723	−60.9 (6)
C46—N41—C42—C32	175.0 (3)	C812—C81—C82—C83	−16 (2)
Fe1—N41—C42—C32	−8.0 (3)	C811—C81—C82—C83	165.3 (13)
N31—C32—C42—N41	2.4 (4)	C812—C81—C82—S821	157.4 (13)
C33—C32—C42—N41	−177.0 (3)	C811—C81—C82—S821	−20.8 (16)
N31—C32—C42—C43	−179.6 (3)	C81—C82—C83—C831	165.9 (16)
C33—C32—C42—C43	0.9 (5)	S821—C82—C83—C831	−8 (2)
N41—C42—C43—C44	1.8 (6)	C81—C82—C83—C832	−25 (2)
C32—C42—C43—C44	−176.0 (4)	S821—C82—C83—C832	161.3 (12)
C42—C43—C44—C45	1.3 (7)	C83—C82—S821—C821	−57.3 (12)
C43—C44—C45—C46	−3.0 (7)	C81—C82—S821—C821	128.5 (9)
C42—N41—C46—C45	1.2 (5)	C82—S821—C821—C822	−63.1 (13)
Fe1—N41—C46—C45	−175.4 (3)	S821—C821—C822—C823	−58.0 (18)

### Tris(2,2'-bipyridine)iron(II) 1,1,3,3-tetracyano-2-(propylsulfanyl)propenide perchlorate (II) Hydrogen-bond geometry (Å, º)

**Table:**

*D*—H···*A*	*D*—H	H···*A*	*D*···*A*	*D*—H···*A*
C15—H15···N832^i^	0.95	2.50	3.267 (13)	138
C24—H24···N731	0.95	2.59	3.471 (6)	154
C35—H35···N712^ii^	0.95	2.57	3.207 (7)	125
C54—H54···N812^iii^	0.95	2.54	3.215 (15)	128
C13—H13···O7^iv^	0.95	2.34	3.258 (10)	163
C33—H33···O10	0.95	2.41	3.351 (17)	172
C43—H43···O10	0.95	2.57	3.521 (17)	174
C53—H53···O3^v^	0.95	2.51	3.432 (9)	165
C63—H63···O5^v^	0.95	2.59	3.512 (8)	163

Symmetry codes: (i) *x*−1, *y*, *z*−1; (ii) *x*−1, *y*, *z*; (iii) −*x*+1/2, *y*−1/2, −*z*+1/2; (iv) −*x*+1/2, *y*+1/2, −*z*+1/2; (v) *x*, *y*, *z*−1.

### Tris(5,5'-dimethyl-2,2'-bipyridine)iron(II) 1,1,3,3-tetracyano-2-methoxypropenide tetrafluoridoborate ethanol 0.926-solvate (III) Crystal data

**Table:**

[Fe(C_12_H_12_N_2_)_3_](C_8_H_3_N_4_O)(BF_4_)·0.926C_2_H_2_O	*F*(000) = 1888
*M**_r_* = 909.18	*D*_x_ = 1.444 Mg m^−^^3^
Monoclinic, *P*2_1_/*n*	Mo *K*α radiation, λ = 0.71073 Å
*a* = 11.6979 (4) Å	Cell parameters from 9605 reflections
*b* = 25.7716 (7) Å	θ = 1.6–28.3°
*c* = 14.1055 (4) Å	µ = 0.43 mm^−^^1^
β = 100.444 (3)°	*T* = 100 K
*V* = 4182.0 (2) Å^3^	Block, red
*Z* = 4	0.29 × 0.24 × 0.20 mm

### Tris(5,5'-dimethyl-2,2'-bipyridine)iron(II) 1,1,3,3-tetracyano-2-methoxypropenide tetrafluoridoborate ethanol 0.926-solvate (III) Data collection

**Table:**

SuperNova, Single source at offset, Eos diffractometer	8711 independent reflections
Radiation source: SuperNova (Mo) X-ray Source	5956 reflections with *I* > 2σ(*I*)
Mirror monochromator	*R*_int_ = 0.090
ω scans	θ_max_ = 26.6°, θ_min_ = 1.6°
Absorption correction: multi-scan (CrysAlis PRO; Rigaku OD, 2015)	*h* = −14→14
*T*_min_ = 0.540, *T*_max_ = 0.917	*k* = −28→32
32301 measured reflections	*l* = −17→17

### Tris(5,5'-dimethyl-2,2'-bipyridine)iron(II) 1,1,3,3-tetracyano-2-methoxypropenide tetrafluoridoborate ethanol 0.926-solvate (III) Refinement

**Table:**

Refinement on *F*^2^	10 restraints
Least-squares matrix: full	Hydrogen site location: inferred from neighbouring sites
*R*[*F*^2^ > 2σ(*F*^2^)] = 0.062	H-atom parameters constrained
*wR*(*F*^2^) = 0.123	*w* = 1/[σ^2^(*F*_o_^2^) + (0.0166*P*)^2^ + 4.487*P*] where *P* = (*F*_o_^2^ + 2*F*_c_^2^)/3
*S* = 1.05	(Δ/σ)_max_ < 0.001
8711 reflections	Δρ_max_ = 0.46 e Å^−^^3^
627 parameters	Δρ_min_ = −0.50 e Å^−^^3^

### Tris(5,5'-dimethyl-2,2'-bipyridine)iron(II) 1,1,3,3-tetracyano-2-methoxypropenide tetrafluoridoborate ethanol 0.926-solvate (III) Special details

**Table:**

Geometry. All esds (except the esd in the dihedral angle between two l.s. planes) are estimated using the full covariance matrix. The cell esds are taken into account individually in the estimation of esds in distances, angles and torsion angles; correlations between esds in cell parameters are only used when they are defined by crystal symmetry. An approximate (isotropic) treatment of cell esds is used for estimating esds involving l.s. planes.

### Tris(5,5'-dimethyl-2,2'-bipyridine)iron(II) 1,1,3,3-tetracyano-2-methoxypropenide tetrafluoridoborate ethanol 0.926-solvate (III) Fractional atomic coordinates and isotropic or equivalent isotropic displacement parameters (Å^2^)

**Table:**

	*x*	*y*	*z*	*U*_iso_*/*U*_eq_	Occ. (<1)
Fe1	0.23513 (4)	0.66041 (2)	0.54608 (3)	0.01398 (12)	
N11	0.1597 (2)	0.70958 (10)	0.62205 (18)	0.0152 (6)	
C12	0.2127 (3)	0.71417 (12)	0.7160 (2)	0.0179 (7)	
C13	0.1676 (3)	0.74606 (13)	0.7796 (2)	0.0245 (8)	
H13	0.2059	0.7491	0.8448	0.029*	
C14	0.0661 (3)	0.77334 (13)	0.7471 (3)	0.0253 (8)	
H14	0.0337	0.7947	0.7904	0.030*	
C15	0.0119 (3)	0.76961 (12)	0.6517 (2)	0.0202 (8)	
C16	0.0621 (3)	0.73702 (12)	0.5925 (2)	0.0169 (7)	
H16	0.0251	0.7339	0.5270	0.020*	
C17	−0.0978 (3)	0.79900 (13)	0.6126 (3)	0.0287 (9)	
H17A	−0.0777	0.8331	0.5895	0.043*	
H17B	−0.1435	0.8035	0.6637	0.043*	
H17C	−0.1434	0.7795	0.5591	0.043*	
N21	0.3519 (2)	0.65828 (10)	0.66713 (18)	0.0154 (6)	
C22	0.3204 (3)	0.68431 (12)	0.7421 (2)	0.0190 (7)	
C23	0.3891 (3)	0.68416 (14)	0.8329 (2)	0.0278 (9)	
H23	0.3648	0.7019	0.8849	0.033*	
C24	0.4934 (3)	0.65773 (13)	0.8467 (2)	0.0255 (8)	
H24	0.5412	0.6571	0.9088	0.031*	
C25	0.5287 (3)	0.63222 (12)	0.7712 (2)	0.0210 (8)	
C26	0.4541 (3)	0.63336 (12)	0.6824 (2)	0.0186 (7)	
H26	0.4768	0.6155	0.6298	0.022*	
C27	0.6410 (3)	0.60315 (13)	0.7820 (3)	0.0296 (9)	
H27A	0.6286	0.5671	0.7994	0.044*	
H27B	0.6983	0.6193	0.8327	0.044*	
H27C	0.6698	0.6040	0.7209	0.044*	
N31	0.1547 (2)	0.59932 (10)	0.58809 (18)	0.0149 (6)	
C32	0.1888 (3)	0.55282 (12)	0.5563 (2)	0.0160 (7)	
C33	0.1390 (3)	0.50670 (12)	0.5792 (2)	0.0202 (8)	
H33	0.1642	0.4745	0.5573	0.024*	
C34	0.0527 (3)	0.50802 (13)	0.6338 (2)	0.0238 (8)	
H34	0.0190	0.4765	0.6503	0.029*	
C35	0.0147 (3)	0.55486 (12)	0.6650 (2)	0.0190 (7)	
C36	0.0702 (3)	0.59912 (13)	0.6410 (2)	0.0175 (7)	
H36	0.0470	0.6315	0.6635	0.021*	
C37	−0.0803 (3)	0.55859 (14)	0.7231 (3)	0.0286 (9)	
H37A	−0.1519	0.5437	0.6864	0.043*	
H37B	−0.0935	0.5951	0.7373	0.043*	
H37C	−0.0576	0.5394	0.7836	0.043*	
N41	0.3126 (2)	0.60601 (9)	0.48313 (18)	0.0142 (6)	
C42	0.2776 (3)	0.55644 (12)	0.4960 (2)	0.0155 (7)	
C43	0.3250 (3)	0.51450 (12)	0.4546 (2)	0.0207 (8)	
H43	0.2994	0.4802	0.4643	0.025*	
C44	0.4091 (3)	0.52288 (12)	0.3993 (2)	0.0201 (8)	
H44	0.4406	0.4944	0.3699	0.024*	
C45	0.4477 (3)	0.57270 (12)	0.3866 (2)	0.0188 (7)	
C46	0.3958 (3)	0.61268 (12)	0.4302 (2)	0.0169 (7)	
H46	0.4213	0.6471	0.4218	0.020*	
C47	0.5417 (3)	0.58434 (13)	0.3303 (3)	0.0277 (9)	
H47A	0.6043	0.5588	0.3462	0.042*	
H47B	0.5726	0.6192	0.3467	0.042*	
H47C	0.5097	0.5827	0.2612	0.042*	
N51	0.1182 (2)	0.67020 (10)	0.42817 (18)	0.0146 (6)	
C52	0.1447 (3)	0.70711 (12)	0.3669 (2)	0.0153 (7)	
C53	0.0688 (3)	0.71869 (12)	0.2819 (2)	0.0210 (8)	
H53	0.0883	0.7449	0.2403	0.025*	
C54	−0.0346 (3)	0.69226 (13)	0.2579 (2)	0.0207 (8)	
H54	−0.0860	0.6997	0.1992	0.025*	
C55	−0.0636 (3)	0.65454 (12)	0.3200 (2)	0.0173 (7)	
C56	0.0161 (3)	0.64516 (11)	0.4042 (2)	0.0155 (7)	
H56	−0.0026	0.6195	0.4472	0.019*	
C57	−0.1747 (3)	0.62445 (13)	0.2997 (2)	0.0243 (8)	
H57A	−0.1635	0.5931	0.2630	0.036*	
H57B	−0.2357	0.6459	0.2620	0.036*	
H57C	−0.1977	0.6146	0.3606	0.036*	
N61	0.3121 (2)	0.71704 (9)	0.48812 (18)	0.0134 (6)	
C62	0.2554 (3)	0.73341 (12)	0.4005 (2)	0.0151 (7)	
C63	0.3026 (3)	0.77140 (12)	0.3495 (2)	0.0181 (7)	
H63	0.2622	0.7824	0.2882	0.022*	
C64	0.4085 (3)	0.79319 (12)	0.3884 (2)	0.0188 (7)	
H64	0.4411	0.8192	0.3537	0.023*	
C65	0.4674 (3)	0.77724 (12)	0.4779 (2)	0.0173 (7)	
C66	0.4149 (3)	0.73861 (12)	0.5243 (2)	0.0159 (7)	
H66	0.4545	0.7269	0.5853	0.019*	
C67	0.5825 (3)	0.79909 (13)	0.5253 (3)	0.0266 (9)	
H67A	0.6215	0.8145	0.4762	0.040*	
H67B	0.6309	0.7713	0.5587	0.040*	
H67C	0.5701	0.8258	0.5719	0.040*	
C71	0.3083 (3)	0.43282 (12)	0.9330 (2)	0.0183 (7)	
C72	0.2302 (3)	0.47463 (13)	0.9136 (2)	0.0191 (7)	
C73	0.2549 (3)	0.52081 (12)	0.8689 (2)	0.0193 (8)	
C711	0.2742 (3)	0.38040 (13)	0.9414 (2)	0.0198 (8)	
N711	0.2510 (3)	0.33711 (11)	0.9471 (2)	0.0269 (7)	
C712	0.4297 (3)	0.43981 (12)	0.9357 (2)	0.0222 (8)	
N712	0.5284 (3)	0.44332 (12)	0.9393 (2)	0.0318 (8)	
O721	0.1229 (2)	0.47321 (9)	0.93472 (16)	0.0235 (6)	
C721	0.1002 (3)	0.44260 (14)	1.0155 (3)	0.0303 (9)	
H72A	0.0900	0.4061	0.9961	0.046*	
H72B	0.0293	0.4553	1.0357	0.046*	
H72C	0.1658	0.4457	1.0693	0.046*	
C731	0.1897 (3)	0.56680 (13)	0.8767 (2)	0.0225 (8)	
N731	0.1388 (3)	0.60490 (12)	0.8799 (2)	0.0308 (8)	
C732	0.3408 (3)	0.52372 (12)	0.8095 (3)	0.0240 (8)	
N732	0.4049 (3)	0.52646 (11)	0.7569 (2)	0.0340 (8)	
B81	0.2551 (7)	0.8374 (3)	1.0460 (5)	0.0243 (19)	0.671 (4)
F81	0.2913 (3)	0.80432 (12)	0.9803 (2)	0.0349 (11)	0.671 (4)
F82	0.2858 (4)	0.88810 (11)	1.0297 (2)	0.0321 (10)	0.671 (4)
F83	0.3080 (4)	0.82296 (13)	1.1381 (2)	0.0556 (15)	0.671 (4)
F84	0.1373 (4)	0.83503 (16)	1.0391 (4)	0.0762 (19)	0.671 (4)
B82	0.2184 (10)	0.8355 (4)	1.0461 (7)	0.0243 (19)	0.329 (4)
F85	0.1924 (7)	0.8123 (2)	1.1278 (4)	0.032 (2)	0.329 (4)
F86	0.1539 (7)	0.8123 (2)	0.9653 (4)	0.040 (2)	0.329 (4)
F87	0.1890 (9)	0.8875 (2)	1.0447 (5)	0.050 (3)	0.329 (4)
F88	0.3339 (6)	0.8302 (4)	1.0444 (8)	0.083 (4)	0.329 (4)
O91	0.7238 (3)	0.47482 (10)	0.8529 (2)	0.0379 (9)	0.926 (5)
H91	0.6601	0.4744	0.8724	0.057*	0.926 (5)
C91	0.7638 (4)	0.42326 (16)	0.8462 (3)	0.0373 (12)	0.926 (5)
H91A	0.6982	0.4007	0.8174	0.045*	0.926 (5)
H91B	0.7958	0.4099	0.9115	0.045*	0.926 (5)
C92	0.8554 (4)	0.42211 (16)	0.7854 (3)	0.0350 (12)	0.926 (5)
H92A	0.9213	0.4436	0.8153	0.053*	0.926 (5)
H92B	0.8236	0.4357	0.7212	0.053*	0.926 (5)
H92C	0.8816	0.3863	0.7798	0.053*	0.926 (5)

### Tris(5,5'-dimethyl-2,2'-bipyridine)iron(II) 1,1,3,3-tetracyano-2-methoxypropenide tetrafluoridoborate ethanol 0.926-solvate (III) Atomic displacement parameters (Å^2^)

**Table:**

	*U*^11^	*U*^22^	*U*^33^	*U*^12^	*U*^13^	*U*^23^
Fe1	0.0157 (3)	0.0132 (2)	0.0134 (2)	−0.00042 (19)	0.00361 (18)	0.00030 (19)
N11	0.0179 (16)	0.0129 (14)	0.0150 (15)	−0.0026 (11)	0.0039 (11)	−0.0022 (11)
C12	0.022 (2)	0.0170 (18)	0.0157 (18)	−0.0081 (14)	0.0073 (14)	−0.0027 (13)
C13	0.028 (2)	0.029 (2)	0.0163 (19)	−0.0043 (16)	0.0047 (15)	−0.0059 (15)
C14	0.030 (2)	0.0208 (19)	0.030 (2)	−0.0067 (15)	0.0173 (17)	−0.0109 (16)
C15	0.021 (2)	0.0148 (18)	0.028 (2)	−0.0031 (14)	0.0127 (15)	−0.0019 (14)
C16	0.024 (2)	0.0129 (17)	0.0150 (18)	−0.0041 (13)	0.0053 (14)	0.0012 (13)
C17	0.031 (2)	0.024 (2)	0.034 (2)	0.0074 (16)	0.0136 (18)	−0.0023 (16)
N21	0.0181 (16)	0.0144 (14)	0.0144 (14)	−0.0049 (11)	0.0047 (11)	0.0021 (11)
C22	0.024 (2)	0.0182 (18)	0.0160 (18)	−0.0042 (14)	0.0069 (14)	−0.0004 (14)
C23	0.029 (2)	0.037 (2)	0.017 (2)	−0.0055 (17)	0.0042 (16)	−0.0018 (16)
C24	0.029 (2)	0.029 (2)	0.0146 (19)	−0.0038 (16)	−0.0066 (15)	0.0050 (15)
C25	0.021 (2)	0.0194 (19)	0.021 (2)	−0.0036 (14)	0.0013 (15)	0.0064 (15)
C26	0.022 (2)	0.0138 (17)	0.0208 (19)	−0.0020 (13)	0.0069 (15)	0.0050 (14)
C27	0.024 (2)	0.025 (2)	0.035 (2)	0.0012 (15)	−0.0073 (17)	0.0044 (17)
N31	0.0175 (16)	0.0146 (14)	0.0116 (14)	0.0016 (11)	0.0004 (11)	0.0018 (11)
C32	0.0179 (19)	0.0169 (17)	0.0116 (17)	0.0000 (13)	−0.0020 (13)	0.0005 (13)
C33	0.023 (2)	0.0143 (17)	0.023 (2)	−0.0011 (14)	0.0047 (15)	0.0027 (14)
C34	0.024 (2)	0.0187 (19)	0.028 (2)	−0.0075 (15)	0.0047 (16)	0.0044 (15)
C35	0.021 (2)	0.0202 (18)	0.0156 (18)	−0.0050 (14)	0.0028 (14)	0.0015 (14)
C36	0.017 (2)	0.0199 (18)	0.0158 (18)	0.0003 (13)	0.0035 (14)	−0.0014 (14)
C37	0.030 (2)	0.033 (2)	0.025 (2)	−0.0075 (17)	0.0104 (17)	0.0005 (16)
N41	0.0172 (16)	0.0106 (14)	0.0146 (15)	−0.0014 (10)	0.0024 (11)	−0.0011 (11)
C42	0.0160 (19)	0.0131 (17)	0.0162 (18)	−0.0003 (13)	−0.0006 (13)	0.0009 (13)
C43	0.027 (2)	0.0129 (17)	0.022 (2)	−0.0021 (14)	0.0038 (15)	−0.0003 (14)
C44	0.021 (2)	0.0186 (18)	0.0209 (19)	0.0028 (14)	0.0049 (15)	−0.0045 (14)
C45	0.017 (2)	0.0184 (18)	0.0207 (19)	0.0004 (13)	0.0038 (14)	−0.0022 (14)
C46	0.0154 (19)	0.0167 (18)	0.0179 (18)	−0.0035 (13)	0.0015 (14)	−0.0002 (13)
C47	0.031 (2)	0.021 (2)	0.036 (2)	0.0030 (16)	0.0183 (18)	−0.0041 (16)
N51	0.0161 (16)	0.0153 (15)	0.0138 (14)	0.0023 (11)	0.0066 (11)	−0.0006 (11)
C52	0.0175 (19)	0.0143 (17)	0.0150 (18)	0.0018 (13)	0.0058 (13)	−0.0029 (13)
C53	0.027 (2)	0.0189 (19)	0.0187 (19)	−0.0006 (14)	0.0071 (15)	0.0027 (14)
C54	0.020 (2)	0.0243 (19)	0.0162 (18)	0.0020 (14)	−0.0010 (14)	0.0021 (14)
C55	0.0194 (19)	0.0154 (17)	0.0171 (18)	0.0013 (13)	0.0031 (14)	−0.0024 (14)
C56	0.0185 (19)	0.0107 (16)	0.0192 (18)	0.0011 (13)	0.0090 (14)	−0.0012 (13)
C57	0.021 (2)	0.028 (2)	0.022 (2)	−0.0022 (15)	−0.0011 (15)	0.0037 (15)
N61	0.0173 (16)	0.0094 (14)	0.0133 (14)	0.0018 (10)	0.0022 (11)	−0.0001 (10)
C62	0.0188 (19)	0.0132 (17)	0.0139 (17)	0.0029 (13)	0.0043 (13)	−0.0001 (13)
C63	0.021 (2)	0.0182 (18)	0.0158 (18)	0.0032 (14)	0.0060 (14)	0.0030 (14)
C64	0.025 (2)	0.0143 (17)	0.0198 (19)	−0.0014 (14)	0.0109 (15)	0.0016 (14)
C65	0.019 (2)	0.0152 (17)	0.0195 (19)	−0.0006 (13)	0.0072 (14)	−0.0001 (14)
C66	0.0134 (18)	0.0157 (17)	0.0198 (18)	0.0003 (13)	0.0061 (14)	−0.0030 (13)
C67	0.027 (2)	0.026 (2)	0.027 (2)	−0.0096 (16)	0.0059 (16)	0.0036 (16)
C71	0.019 (2)	0.0197 (18)	0.0163 (18)	−0.0005 (14)	0.0046 (14)	0.0006 (14)
C72	0.020 (2)	0.0237 (19)	0.0134 (18)	−0.0018 (14)	0.0029 (14)	−0.0017 (14)
C73	0.023 (2)	0.0177 (18)	0.0167 (19)	−0.0025 (14)	0.0036 (14)	0.0005 (14)
C711	0.017 (2)	0.024 (2)	0.0180 (19)	0.0024 (15)	0.0028 (14)	0.0005 (15)
N711	0.0275 (19)	0.0246 (18)	0.0283 (18)	−0.0004 (14)	0.0043 (13)	0.0047 (14)
C712	0.027 (2)	0.0166 (18)	0.024 (2)	−0.0003 (15)	0.0067 (16)	−0.0055 (14)
N712	0.019 (2)	0.0362 (19)	0.040 (2)	0.0001 (14)	0.0063 (15)	−0.0056 (15)
O721	0.0138 (14)	0.0333 (14)	0.0249 (14)	0.0001 (10)	0.0075 (10)	0.0084 (11)
C721	0.025 (2)	0.033 (2)	0.036 (2)	0.0036 (17)	0.0157 (17)	0.0071 (18)
C731	0.031 (2)	0.025 (2)	0.0104 (18)	−0.0027 (16)	0.0027 (15)	−0.0017 (14)
N731	0.039 (2)	0.0284 (19)	0.0265 (19)	0.0048 (15)	0.0104 (15)	−0.0040 (14)
C732	0.029 (2)	0.0137 (18)	0.030 (2)	−0.0018 (15)	0.0067 (17)	0.0002 (15)
N732	0.045 (2)	0.0175 (17)	0.046 (2)	−0.0011 (14)	0.0280 (18)	−0.0004 (14)
B81	0.029 (6)	0.023 (3)	0.024 (3)	0.003 (3)	0.015 (3)	−0.001 (2)
F81	0.059 (3)	0.0235 (19)	0.023 (2)	−0.0039 (16)	0.0113 (18)	−0.0096 (14)
F82	0.048 (3)	0.0201 (18)	0.028 (2)	0.0005 (16)	0.0066 (17)	0.0020 (13)
F83	0.110 (4)	0.035 (2)	0.022 (2)	0.020 (2)	0.013 (2)	0.0060 (16)
F84	0.036 (3)	0.055 (3)	0.145 (6)	−0.002 (2)	0.035 (3)	0.006 (3)
B82	0.029 (6)	0.023 (3)	0.024 (3)	0.003 (3)	0.015 (3)	−0.001 (2)
F85	0.061 (6)	0.021 (4)	0.013 (4)	0.001 (3)	0.005 (3)	0.006 (3)
F86	0.077 (7)	0.029 (4)	0.010 (4)	−0.015 (4)	−0.002 (3)	0.001 (3)
F87	0.101 (9)	0.010 (4)	0.031 (4)	0.009 (4)	−0.012 (5)	−0.004 (3)
F88	0.031 (6)	0.098 (9)	0.131 (11)	0.019 (5)	0.041 (6)	0.056 (8)
O91	0.035 (2)	0.0248 (17)	0.062 (2)	0.0035 (13)	0.0311 (16)	−0.0011 (14)
C91	0.045 (3)	0.032 (3)	0.039 (3)	0.012 (2)	0.018 (2)	0.014 (2)
C92	0.039 (3)	0.034 (3)	0.035 (3)	0.012 (2)	0.014 (2)	0.006 (2)

### Tris(5,5'-dimethyl-2,2'-bipyridine)iron(II) 1,1,3,3-tetracyano-2-methoxypropenide tetrafluoridoborate ethanol 0.926-solvate (III) Geometric parameters (Å, º)

**Table:**

Fe1—N41	1.967 (3)	C47—H47C	0.9800
Fe1—N11	1.968 (3)	N51—C56	1.345 (4)
Fe1—N61	1.969 (3)	N51—C52	1.359 (4)
Fe1—N51	1.969 (3)	C52—C53	1.388 (4)
Fe1—N31	1.979 (3)	C52—C62	1.462 (4)
Fe1—N21	1.985 (3)	C53—C54	1.376 (5)
N11—C16	1.344 (4)	C53—H53	0.9500
N11—C12	1.362 (4)	C54—C55	1.391 (4)
C12—C13	1.390 (4)	C54—H54	0.9500
C12—C22	1.465 (5)	C55—C56	1.391 (4)
C13—C14	1.384 (5)	C55—C57	1.496 (4)
C13—H13	0.9500	C56—H56	0.9500
C14—C15	1.384 (5)	C57—H57A	0.9800
C14—H14	0.9500	C57—H57B	0.9800
C15—C16	1.387 (4)	C57—H57C	0.9800
C15—C17	1.505 (5)	N61—C66	1.339 (4)
C16—H16	0.9500	N61—C62	1.360 (4)
C17—H17A	0.9800	C62—C63	1.387 (4)
C17—H17B	0.9800	C63—C64	1.380 (5)
C17—H17C	0.9800	C63—H63	0.9500
N21—C26	1.340 (4)	C64—C65	1.386 (4)
N21—C22	1.359 (4)	C64—H64	0.9500
C22—C23	1.384 (5)	C65—C66	1.394 (4)
C23—C24	1.380 (5)	C65—C67	1.500 (5)
C23—H23	0.9500	C66—H66	0.9500
C24—C25	1.378 (5)	C67—H67A	0.9800
C24—H24	0.9500	C67—H67B	0.9800
C25—C26	1.391 (5)	C67—H67C	0.9800
C25—C27	1.496 (5)	C71—C72	1.407 (4)
C26—H26	0.9500	C71—C711	1.420 (5)
C27—H27A	0.9800	C71—C712	1.425 (5)
C27—H27B	0.9800	C72—O721	1.341 (4)
C27—H27C	0.9800	C72—C73	1.402 (4)
N31—C36	1.342 (4)	C73—C732	1.423 (5)
N31—C32	1.364 (4)	C73—C731	1.424 (5)
C32—C33	1.387 (4)	C711—N711	1.154 (4)
C32—C42	1.460 (4)	C712—N712	1.151 (4)
C33—C34	1.377 (5)	O721—C721	1.450 (4)
C33—H33	0.9500	C721—H72A	0.9800
C34—C35	1.386 (5)	C721—H72B	0.9800
C34—H34	0.9500	C721—H72C	0.9800
C35—C36	1.385 (4)	C731—N731	1.153 (4)
C35—C37	1.498 (5)	C732—N732	1.148 (4)
C36—H36	0.9500	B81—F84	1.365 (9)
C37—H37A	0.9800	B81—F81	1.382 (8)
C37—H37B	0.9800	B81—F83	1.385 (8)
C37—H37C	0.9800	B81—F82	1.385 (8)
N41—C46	1.341 (4)	B82—F88	1.363 (10)
N41—C42	1.364 (4)	B82—F85	1.380 (9)
C42—C43	1.391 (4)	B82—F87	1.383 (9)
C43—C44	1.378 (5)	B82—F86	1.384 (9)
C43—H43	0.9500	O91—C91	1.418 (4)
C44—C45	1.384 (4)	O91—H91	0.8400
C44—H44	0.9500	C91—C92	1.489 (6)
C45—C46	1.394 (4)	C91—H91A	0.9900
C45—C47	1.498 (5)	C91—H91B	0.9900
C46—H46	0.9500	C92—H92A	0.9800
C47—H47A	0.9800	C92—H92B	0.9800
C47—H47B	0.9800	C92—H92C	0.9800
			
N41—Fe1—N11	173.43 (11)	N41—C46—C45	124.7 (3)
N41—Fe1—N61	93.32 (11)	N41—C46—H46	117.7
N11—Fe1—N61	91.88 (10)	C45—C46—H46	117.7
N41—Fe1—N51	90.95 (11)	C45—C47—H47A	109.5
N11—Fe1—N51	93.79 (11)	C45—C47—H47B	109.5
N61—Fe1—N51	81.61 (11)	H47A—C47—H47B	109.5
N41—Fe1—N31	81.45 (11)	C45—C47—H47C	109.5
N11—Fe1—N31	93.71 (11)	H47A—C47—H47C	109.5
N61—Fe1—N31	172.76 (11)	H47B—C47—H47C	109.5
N51—Fe1—N31	93.42 (11)	C56—N51—C52	118.0 (3)
N41—Fe1—N21	93.83 (11)	C56—N51—Fe1	126.9 (2)
N11—Fe1—N21	81.72 (11)	C52—N51—Fe1	115.1 (2)
N61—Fe1—N21	94.73 (11)	N51—C52—C53	121.0 (3)
N51—Fe1—N21	174.15 (10)	N51—C52—C62	114.2 (3)
N31—Fe1—N21	90.62 (10)	C53—C52—C62	124.8 (3)
C16—N11—C12	117.9 (3)	C54—C53—C52	120.2 (3)
C16—N11—Fe1	127.6 (2)	C54—C53—H53	119.9
C12—N11—Fe1	114.5 (2)	C52—C53—H53	119.9
N11—C12—C13	121.3 (3)	C53—C54—C55	119.6 (3)
N11—C12—C22	114.7 (3)	C53—C54—H54	120.2
C13—C12—C22	123.9 (3)	C55—C54—H54	120.2
C14—C13—C12	119.3 (3)	C56—C55—C54	117.2 (3)
C14—C13—H13	120.3	C56—C55—C57	119.9 (3)
C12—C13—H13	120.3	C54—C55—C57	122.9 (3)
C13—C14—C15	120.1 (3)	N51—C56—C55	124.0 (3)
C13—C14—H14	120.0	N51—C56—H56	118.0
C15—C14—H14	120.0	C55—C56—H56	118.0
C14—C15—C16	117.2 (3)	C55—C57—H57A	109.5
C14—C15—C17	122.1 (3)	C55—C57—H57B	109.5
C16—C15—C17	120.7 (3)	H57A—C57—H57B	109.5
N11—C16—C15	124.1 (3)	C55—C57—H57C	109.5
N11—C16—H16	117.9	H57A—C57—H57C	109.5
C15—C16—H16	117.9	H57B—C57—H57C	109.5
C15—C17—H17A	109.5	C66—N61—C62	118.3 (3)
C15—C17—H17B	109.5	C66—N61—Fe1	126.6 (2)
H17A—C17—H17B	109.5	C62—N61—Fe1	115.1 (2)
C15—C17—H17C	109.5	N61—C62—C63	121.1 (3)
H17A—C17—H17C	109.5	N61—C62—C52	114.1 (3)
H17B—C17—H17C	109.5	C63—C62—C52	124.8 (3)
C26—N21—C22	118.2 (3)	C64—C63—C62	119.6 (3)
C26—N21—Fe1	127.3 (2)	C64—C63—H63	120.2
C22—N21—Fe1	114.5 (2)	C62—C63—H63	120.2
N21—C22—C23	121.7 (3)	C63—C64—C65	120.2 (3)
N21—C22—C12	114.0 (3)	C63—C64—H64	119.9
C23—C22—C12	124.3 (3)	C65—C64—H64	119.9
C24—C23—C22	118.8 (3)	C64—C65—C66	116.8 (3)
C24—C23—H23	120.6	C64—C65—C67	123.5 (3)
C22—C23—H23	120.6	C66—C65—C67	119.7 (3)
C25—C24—C23	120.5 (3)	N61—C66—C65	124.0 (3)
C25—C24—H24	119.7	N61—C66—H66	118.0
C23—C24—H24	119.7	C65—C66—H66	118.0
C24—C25—C26	117.3 (3)	C65—C67—H67A	109.5
C24—C25—C27	122.8 (3)	C65—C67—H67B	109.5
C26—C25—C27	119.9 (3)	H67A—C67—H67B	109.5
N21—C26—C25	123.4 (3)	C65—C67—H67C	109.5
N21—C26—H26	118.3	H67A—C67—H67C	109.5
C25—C26—H26	118.3	H67B—C67—H67C	109.5
C25—C27—H27A	109.5	C72—C71—C711	124.2 (3)
C25—C27—H27B	109.5	C72—C71—C712	121.1 (3)
H27A—C27—H27B	109.5	C711—C71—C712	114.3 (3)
C25—C27—H27C	109.5	O721—C72—C73	113.6 (3)
H27A—C27—H27C	109.5	O721—C72—C71	122.4 (3)
H27B—C27—H27C	109.5	C73—C72—C71	123.9 (3)
C36—N31—C32	117.9 (3)	C72—C73—C732	122.4 (3)
C36—N31—Fe1	127.4 (2)	C72—C73—C731	121.0 (3)
C32—N31—Fe1	114.7 (2)	C732—C73—C731	116.5 (3)
N31—C32—C33	121.1 (3)	N711—C711—C71	177.0 (4)
N31—C32—C42	114.6 (3)	N712—C712—C71	177.1 (4)
C33—C32—C42	124.3 (3)	C72—O721—C721	120.1 (3)
C34—C33—C32	119.3 (3)	O721—C721—H72A	109.5
C34—C33—H33	120.3	O721—C721—H72B	109.5
C32—C33—H33	120.3	H72A—C721—H72B	109.5
C33—C34—C35	120.6 (3)	O721—C721—H72C	109.5
C33—C34—H34	119.7	H72A—C721—H72C	109.5
C35—C34—H34	119.7	H72B—C721—H72C	109.5
C36—C35—C34	116.6 (3)	N731—C731—C73	177.2 (4)
C36—C35—C37	120.6 (3)	N732—C732—C73	175.9 (4)
C34—C35—C37	122.8 (3)	F84—B81—F81	110.7 (6)
N31—C36—C35	124.4 (3)	F84—B81—F83	109.2 (6)
N31—C36—H36	117.8	F81—B81—F83	108.9 (6)
C35—C36—H36	117.8	F84—B81—F82	108.6 (6)
C35—C37—H37A	109.5	F81—B81—F82	110.5 (5)
C35—C37—H37B	109.5	F83—B81—F82	108.9 (6)
H37A—C37—H37B	109.5	F88—B82—F85	109.9 (8)
C35—C37—H37C	109.5	F88—B82—F87	109.9 (8)
H37A—C37—H37C	109.5	F85—B82—F87	110.0 (8)
H37B—C37—H37C	109.5	F88—B82—F86	109.7 (8)
C46—N41—C42	117.4 (3)	F85—B82—F86	109.3 (7)
C46—N41—Fe1	126.9 (2)	F87—B82—F86	108.0 (7)
C42—N41—Fe1	115.7 (2)	C91—O91—H91	109.5
N41—C42—C43	121.3 (3)	O91—C91—C92	109.7 (3)
N41—C42—C32	113.6 (3)	O91—C91—H91A	109.7
C43—C42—C32	125.1 (3)	C92—C91—H91A	109.7
C44—C43—C42	119.7 (3)	O91—C91—H91B	109.7
C44—C43—H43	120.1	C92—C91—H91B	109.7
C42—C43—H43	120.1	H91A—C91—H91B	108.2
C43—C44—C45	120.2 (3)	C91—C92—H92A	109.5
C43—C44—H44	119.9	C91—C92—H92B	109.5
C45—C44—H44	119.9	H92A—C92—H92B	109.5
C44—C45—C46	116.7 (3)	C91—C92—H92C	109.5
C44—C45—C47	122.9 (3)	H92A—C92—H92C	109.5
C46—C45—C47	120.4 (3)	H92B—C92—H92C	109.5
			
C16—N11—C12—C13	0.2 (5)	C33—C32—C42—C43	−0.8 (5)
Fe1—N11—C12—C13	−177.8 (2)	N41—C42—C43—C44	0.0 (5)
C16—N11—C12—C22	−178.3 (3)	C32—C42—C43—C44	−179.6 (3)
Fe1—N11—C12—C22	3.7 (3)	C42—C43—C44—C45	1.2 (5)
N11—C12—C13—C14	0.6 (5)	C43—C44—C45—C46	−1.3 (5)
C22—C12—C13—C14	178.9 (3)	C43—C44—C45—C47	177.9 (3)
C12—C13—C14—C15	−1.3 (5)	C42—N41—C46—C45	0.9 (5)
C13—C14—C15—C16	1.3 (5)	Fe1—N41—C46—C45	−179.7 (2)
C13—C14—C15—C17	−179.3 (3)	C44—C45—C46—N41	0.3 (5)
C12—N11—C16—C15	−0.1 (5)	C47—C45—C46—N41	−178.9 (3)
Fe1—N11—C16—C15	177.5 (2)	C56—N51—C52—C53	0.0 (4)
C14—C15—C16—N11	−0.6 (5)	Fe1—N51—C52—C53	−178.7 (2)
C17—C15—C16—N11	180.0 (3)	C56—N51—C52—C62	178.1 (3)
C26—N21—C22—C23	−2.0 (5)	Fe1—N51—C52—C62	−0.7 (3)
Fe1—N21—C22—C23	176.3 (3)	N51—C52—C53—C54	−0.8 (5)
C26—N21—C22—C12	174.6 (3)	C62—C52—C53—C54	−178.6 (3)
Fe1—N21—C22—C12	−7.1 (3)	C52—C53—C54—C55	1.2 (5)
N11—C12—C22—N21	2.2 (4)	C53—C54—C55—C56	−0.7 (5)
C13—C12—C22—N21	−176.2 (3)	C53—C54—C55—C57	179.4 (3)
N11—C12—C22—C23	178.8 (3)	C52—N51—C56—C55	0.4 (5)
C13—C12—C22—C23	0.3 (5)	Fe1—N51—C56—C55	179.0 (2)
N21—C22—C23—C24	1.4 (5)	C54—C55—C56—N51	−0.1 (5)
C12—C22—C23—C24	−174.8 (3)	C57—C55—C56—N51	179.8 (3)
C22—C23—C24—C25	0.4 (5)	C66—N61—C62—C63	0.1 (4)
C23—C24—C25—C26	−1.4 (5)	Fe1—N61—C62—C63	−177.0 (2)
C23—C24—C25—C27	179.8 (3)	C66—N61—C62—C52	179.4 (3)
C22—N21—C26—C25	0.9 (5)	Fe1—N61—C62—C52	2.3 (3)
Fe1—N21—C26—C25	−177.2 (2)	N51—C52—C62—N61	−1.0 (4)
C24—C25—C26—N21	0.8 (5)	C53—C52—C62—N61	176.9 (3)
C27—C25—C26—N21	179.6 (3)	N51—C52—C62—C63	178.2 (3)
C36—N31—C32—C33	−0.9 (4)	C53—C52—C62—C63	−3.8 (5)
Fe1—N31—C32—C33	−179.3 (2)	N61—C62—C63—C64	−0.3 (5)
C36—N31—C32—C42	177.6 (3)	C52—C62—C63—C64	−179.5 (3)
Fe1—N31—C32—C42	−0.7 (3)	C62—C63—C64—C65	0.0 (5)
N31—C32—C33—C34	0.8 (5)	C63—C64—C65—C66	0.5 (5)
C42—C32—C33—C34	−177.6 (3)	C63—C64—C65—C67	−179.8 (3)
C32—C33—C34—C35	0.7 (5)	C62—N61—C66—C65	0.5 (5)
C33—C34—C35—C36	−2.0 (5)	Fe1—N61—C66—C65	177.2 (2)
C33—C34—C35—C37	178.9 (3)	C64—C65—C66—N61	−0.8 (5)
C32—N31—C36—C35	−0.5 (5)	C67—C65—C66—N61	179.5 (3)
Fe1—N31—C36—C35	177.6 (2)	C711—C71—C72—O721	24.6 (5)
C34—C35—C36—N31	1.9 (5)	C712—C71—C72—O721	−162.2 (3)
C37—C35—C36—N31	−178.9 (3)	C711—C71—C72—C73	−153.8 (3)
C46—N41—C42—C43	−1.0 (5)	C712—C71—C72—C73	19.5 (5)
Fe1—N41—C42—C43	179.4 (2)	O721—C72—C73—C732	−156.0 (3)
C46—N41—C42—C32	178.7 (3)	C71—C72—C73—C732	22.5 (5)
Fe1—N41—C42—C32	−0.9 (3)	O721—C72—C73—C731	20.5 (5)
N31—C32—C42—N41	1.0 (4)	C71—C72—C73—C731	−161.0 (3)
C33—C32—C42—N41	179.5 (3)	C73—C72—O721—C721	−151.6 (3)
N31—C32—C42—C43	−179.3 (3)	C71—C72—O721—C721	29.9 (5)

### Tris(5,5'-dimethyl-2,2'-bipyridine)iron(II) 1,1,3,3-tetracyano-2-methoxypropenide tetrafluoridoborate ethanol 0.926-solvate (III) Hydrogen-bond geometry (Å, º)

**Table:**

*D*—H···*A*	*D*—H	H···*A*	*D*···*A*	*D*—H···*A*
O91—H91···N712	0.84	2.11	2.895 (5)	156
C13—H13···F81	0.95	2.45	3.298 (4)	149
C43—H43···F87^i^	0.95	2.40	3.277 (6)	154
C63—H63···F83^ii^	0.95	2.50	3.276 (4)	138
C63—H63···F85^ii^	0.95	2.39	3.330 (6)	170

Symmetry codes: (i) −*x*+1/2, *y*−1/2, −*z*+3/2; (ii) *x*, *y*, *z*−1.

### Tris(5,5'-dimethyl-2,2'-bipyridine)iron(II) 1,1,3,3-tetracyano-2-ethoxypropenide tetrafluoridoborate (IV) Crystal data

**Table:**

[Fe(C_12_H_12_N_2_)_3_](C_9_H_5_N_4_O)(BF_4_)	*F*(000) = 1824
*M**_r_* = 880.54	*D*_x_ = 1.406 Mg m^−^^3^
Monoclinic, *P*2_1_/*n*	Cu *K*α radiation, λ = 1.54184 Å
*a* = 11.5865 (3) Å	Cell parameters from 7609 reflections
*b* = 25.5914 (5) Å	θ = 4.3–68.3°
*c* = 14.4997 (3) Å	µ = 3.48 mm^−^^1^
β = 104.641 (3)°	*T* = 100 K
*V* = 4159.77 (17) Å^3^	Needle, red
*Z* = 4	0.14 × 0.03 × 0.02 mm

### Tris(5,5'-dimethyl-2,2'-bipyridine)iron(II) 1,1,3,3-tetracyano-2-ethoxypropenide tetrafluoridoborate (IV) Data collection

**Table:**

Rigaku XtaLAB Synergy-S diffractometer	7607 independent reflections
Radiation source: sealed tube	5392 reflections with *I* > 2σ(*I*)
Detector resolution: 5.811 pixels mm^-1^	*R*_int_ = 0.079
ω scans	θ_max_ = 68.3°, θ_min_ = 4.3°
Absorption correction: multi-scan (CrysAlis PRO; Rigaku OD, 2015)	*h* = −13→13
*T*_min_ = 0.746, *T*_max_ = 0.920	*k* = −30→28
30853 measured reflections	*l* = −17→17

### Tris(5,5'-dimethyl-2,2'-bipyridine)iron(II) 1,1,3,3-tetracyano-2-ethoxypropenide tetrafluoridoborate (IV) Refinement

**Table:**

Refinement on *F*^2^	0 restraints
Least-squares matrix: full	Hydrogen site location: inferred from neighbouring sites
*R*[*F*^2^ > 2σ(*F*^2^)] = 0.059	H-atom parameters constrained
*wR*(*F*^2^) = 0.162	*w* = 1/[σ^2^(*F*_o_^2^) + (0.0959*P*)^2^ + 0.2678*P*] where *P* = (*F*_o_^2^ + 2*F*_c_^2^)/3
*S* = 1.02	(Δ/σ)_max_ = 0.001
7607 reflections	Δρ_max_ = 1.71 e Å^−^^3^
566 parameters	Δρ_min_ = −0.45 e Å^−^^3^

### Tris(5,5'-dimethyl-2,2'-bipyridine)iron(II) 1,1,3,3-tetracyano-2-ethoxypropenide tetrafluoridoborate (IV) Special details

**Table:**

Geometry. All esds (except the esd in the dihedral angle between two l.s. planes) are estimated using the full covariance matrix. The cell esds are taken into account individually in the estimation of esds in distances, angles and torsion angles; correlations between esds in cell parameters are only used when they are defined by crystal symmetry. An approximate (isotropic) treatment of cell esds is used for estimating esds involving l.s. planes.

### Tris(5,5'-dimethyl-2,2'-bipyridine)iron(II) 1,1,3,3-tetracyano-2-ethoxypropenide tetrafluoridoborate (IV) Fractional atomic coordinates and isotropic or equivalent isotropic displacement parameters (Å^2^)

**Table:**

	*x*	*y*	*z*	*U*_iso_*/*U*_eq_	
Fe1	0.71216 (4)	0.66268 (2)	0.53105 (3)	0.01552 (15)	
N11	0.8424 (2)	0.66448 (10)	0.64907 (18)	0.0187 (6)	
C12	0.8204 (3)	0.69464 (13)	0.7197 (2)	0.0212 (7)	
C13	0.9020 (3)	0.69792 (15)	0.8081 (2)	0.0284 (8)	
H13	0.8847	0.7186	0.8573	0.034*	
C14	1.0090 (3)	0.67082 (15)	0.8243 (2)	0.0299 (8)	
H14	1.0650	0.6727	0.8846	0.036*	
C15	1.0336 (3)	0.64100 (14)	0.7520 (2)	0.0247 (7)	
C16	0.9465 (3)	0.63894 (13)	0.6657 (2)	0.0217 (7)	
H16	0.9621	0.6182	0.6158	0.026*	
C17	1.1465 (3)	0.61016 (15)	0.7631 (3)	0.0309 (8)	
H17A	1.2087	0.6247	0.8156	0.037*	
H17B	1.1725	0.6121	0.7039	0.037*	
H17C	1.1319	0.5736	0.7768	0.037*	
N21	0.6437 (2)	0.71507 (10)	0.60122 (18)	0.0191 (6)	
C22	0.7071 (3)	0.72324 (13)	0.6930 (2)	0.0202 (7)	
C23	0.6654 (3)	0.75624 (14)	0.7527 (2)	0.0276 (8)	
H23	0.7096	0.7607	0.8170	0.033*	
C24	0.5595 (3)	0.78264 (14)	0.7189 (3)	0.0287 (8)	
H24	0.5306	0.8054	0.7598	0.034*	
C25	0.4944 (3)	0.77580 (13)	0.6242 (2)	0.0236 (7)	
C26	0.5406 (3)	0.74147 (12)	0.5692 (2)	0.0204 (7)	
H26	0.4972	0.7362	0.5049	0.024*	
C27	0.3798 (3)	0.80440 (15)	0.5836 (3)	0.0330 (9)	
H27A	0.3350	0.7866	0.5258	0.040*	
H27B	0.3974	0.8403	0.5679	0.040*	
H27C	0.3322	0.8050	0.6306	0.040*	
N31	0.7832 (2)	0.71674 (10)	0.46615 (17)	0.0161 (5)	
C32	0.7264 (3)	0.72422 (13)	0.3727 (2)	0.0197 (7)	
C33	0.7738 (3)	0.75744 (14)	0.3157 (2)	0.0258 (8)	
H33	0.7327	0.7627	0.2508	0.031*	
C34	0.8809 (3)	0.78276 (14)	0.3536 (2)	0.0256 (8)	
H34	0.9147	0.8048	0.3146	0.031*	
C35	0.9388 (3)	0.77567 (13)	0.4498 (2)	0.0239 (7)	
C36	0.8858 (3)	0.74261 (13)	0.5023 (2)	0.0208 (7)	
H36	0.9241	0.7378	0.5679	0.025*	
C37	1.0541 (3)	0.80248 (16)	0.4964 (3)	0.0342 (9)	
H37A	1.1047	0.7787	0.5423	0.041*	
H37B	1.0375	0.8339	0.5296	0.041*	
H37C	1.0952	0.8124	0.4478	0.041*	
N41	0.5859 (2)	0.66637 (10)	0.41076 (18)	0.0194 (6)	
C42	0.6155 (3)	0.69527 (13)	0.3415 (2)	0.0216 (7)	
C43	0.5431 (3)	0.69665 (15)	0.2490 (2)	0.0292 (8)	
H43	0.5661	0.7164	0.2012	0.035*	
C44	0.4366 (3)	0.66876 (14)	0.2273 (2)	0.0277 (8)	
H44	0.3870	0.6690	0.1641	0.033*	
C45	0.4032 (3)	0.64068 (13)	0.2980 (2)	0.0233 (7)	
C46	0.4806 (3)	0.64057 (13)	0.3890 (2)	0.0207 (7)	
H46	0.4583	0.6215	0.4380	0.025*	
C47	0.2875 (3)	0.61117 (15)	0.2799 (3)	0.0293 (8)	
H47A	0.2616	0.6090	0.3391	0.035*	
H47B	0.2988	0.5759	0.2575	0.035*	
H47C	0.2267	0.6294	0.2313	0.035*	
N51	0.7866 (2)	0.60583 (10)	0.47408 (18)	0.0180 (6)	
C52	0.7539 (3)	0.55665 (13)	0.4919 (2)	0.0216 (7)	
C53	0.7990 (3)	0.51341 (14)	0.4555 (3)	0.0280 (8)	
H53	0.7727	0.4794	0.4666	0.034*	
C54	0.8826 (3)	0.51987 (14)	0.4030 (3)	0.0296 (8)	
H54	0.9144	0.4903	0.3783	0.035*	
C55	0.9196 (3)	0.57003 (14)	0.3869 (2)	0.0265 (8)	
C56	0.8673 (3)	0.61166 (13)	0.4231 (2)	0.0223 (7)	
H56	0.8902	0.6461	0.4109	0.027*	
C57	1.0124 (4)	0.58083 (16)	0.3331 (3)	0.0379 (9)	
H57A	1.0691	0.5517	0.3422	0.046*	
H57B	1.0550	0.6131	0.3570	0.046*	
H57C	0.9736	0.5847	0.2651	0.046*	
N61	0.6386 (2)	0.60250 (10)	0.58053 (18)	0.0190 (6)	
C62	0.6703 (3)	0.55499 (13)	0.5527 (2)	0.0221 (7)	
C63	0.6272 (3)	0.50910 (14)	0.5827 (3)	0.0274 (8)	
H63	0.6493	0.4762	0.5623	0.033*	
C64	0.5518 (3)	0.51195 (14)	0.6428 (3)	0.0288 (8)	
H64	0.5225	0.4808	0.6643	0.035*	
C65	0.5187 (3)	0.56011 (14)	0.6717 (2)	0.0233 (7)	
C66	0.5639 (3)	0.60411 (14)	0.6381 (2)	0.0229 (7)	
H66	0.5411	0.6374	0.6566	0.028*	
C67	0.4379 (3)	0.56508 (16)	0.7378 (3)	0.0322 (9)	
H67A	0.3721	0.5401	0.7190	0.039*	
H67B	0.4058	0.6007	0.7341	0.039*	
H67C	0.4833	0.5578	0.8032	0.039*	
C71	0.1744 (4)	0.54550 (15)	0.0579 (2)	0.0318 (9)	
C72	0.2338 (3)	0.49738 (16)	0.0758 (2)	0.0299 (8)	
C73	0.2126 (3)	0.45855 (14)	0.1363 (2)	0.0271 (8)	
C711	0.2278 (4)	0.58963 (16)	0.0278 (3)	0.0423 (11)	
N711	0.2731 (4)	0.62652 (16)	0.0060 (3)	0.0597 (12)	
C712	0.0602 (4)	0.55320 (15)	0.0755 (3)	0.0363 (10)	
N712	−0.0338 (4)	0.56125 (15)	0.0871 (3)	0.0471 (9)	
O721	0.3239 (3)	0.48641 (11)	0.03499 (18)	0.0379 (7)	
C721	0.3072 (5)	0.49718 (19)	−0.0669 (3)	0.0469 (11)	
H71A	0.3536	0.5284	−0.0761	0.056*	
H71B	0.2219	0.5036	−0.0978	0.056*	
C722	0.3505 (5)	0.4498 (2)	−0.1091 (3)	0.0586 (14)	
H72A	0.3005	0.4198	−0.1032	0.070*	
H72B	0.4334	0.4426	−0.0751	0.070*	
H72C	0.3459	0.4562	−0.1766	0.070*	
C731	0.2634 (3)	0.40833 (15)	0.1350 (2)	0.0279 (8)	
N731	0.3046 (3)	0.36701 (13)	0.1381 (2)	0.0368 (8)	
C732	0.1465 (3)	0.46658 (14)	0.2054 (3)	0.0280 (8)	
N732	0.0969 (3)	0.47074 (13)	0.2642 (3)	0.0400 (8)	
B81	0.7572 (4)	0.33719 (17)	0.4655 (3)	0.0304 (9)	
F81	0.7288 (2)	0.30678 (9)	0.53652 (14)	0.0371 (5)	
F82	0.7235 (2)	0.38882 (9)	0.47707 (16)	0.0406 (6)	
F83	0.6936 (2)	0.31925 (9)	0.37666 (15)	0.0424 (6)	
F84	0.8774 (2)	0.33502 (12)	0.4723 (2)	0.0586 (8)	

### Tris(5,5'-dimethyl-2,2'-bipyridine)iron(II) 1,1,3,3-tetracyano-2-ethoxypropenide tetrafluoridoborate (IV) Atomic displacement parameters (Å^2^)

**Table:**

	*U*^11^	*U*^22^	*U*^33^	*U*^12^	*U*^13^	*U*^23^
Fe1	0.0185 (3)	0.0136 (3)	0.0135 (2)	−0.0002 (2)	0.00227 (18)	0.0000 (2)
N11	0.0212 (14)	0.0166 (13)	0.0174 (13)	−0.0026 (11)	0.0032 (11)	0.0036 (11)
C12	0.0249 (17)	0.0207 (17)	0.0183 (15)	−0.0061 (14)	0.0059 (13)	−0.0022 (13)
C13	0.0299 (19)	0.036 (2)	0.0177 (16)	−0.0055 (16)	0.0025 (14)	−0.0042 (15)
C14	0.0292 (19)	0.035 (2)	0.0202 (16)	−0.0035 (16)	−0.0033 (15)	0.0032 (15)
C15	0.0220 (17)	0.0239 (18)	0.0244 (17)	−0.0041 (14)	−0.0014 (14)	0.0061 (14)
C16	0.0226 (17)	0.0181 (16)	0.0226 (16)	−0.0019 (13)	0.0027 (14)	0.0030 (13)
C17	0.0262 (19)	0.036 (2)	0.0264 (18)	0.0037 (16)	−0.0008 (15)	0.0050 (16)
N21	0.0223 (14)	0.0164 (13)	0.0187 (13)	−0.0051 (11)	0.0055 (11)	0.0002 (11)
C22	0.0248 (17)	0.0210 (16)	0.0157 (14)	−0.0046 (14)	0.0065 (13)	−0.0036 (13)
C23	0.0290 (19)	0.0295 (19)	0.0245 (17)	−0.0058 (15)	0.0067 (15)	−0.0085 (15)
C24	0.032 (2)	0.0247 (19)	0.0331 (19)	−0.0049 (16)	0.0157 (16)	−0.0106 (16)
C25	0.0283 (18)	0.0155 (16)	0.0293 (17)	−0.0027 (14)	0.0113 (15)	−0.0019 (14)
C26	0.0233 (17)	0.0173 (16)	0.0208 (15)	−0.0018 (13)	0.0063 (13)	0.0017 (13)
C27	0.031 (2)	0.0255 (19)	0.043 (2)	0.0042 (16)	0.0101 (17)	−0.0031 (17)
N31	0.0184 (13)	0.0156 (13)	0.0145 (12)	0.0032 (11)	0.0045 (10)	−0.0015 (11)
C32	0.0254 (17)	0.0163 (16)	0.0177 (15)	0.0041 (13)	0.0062 (13)	0.0010 (13)
C33	0.0321 (19)	0.0274 (18)	0.0185 (15)	0.0062 (15)	0.0075 (14)	0.0073 (14)
C34	0.0279 (19)	0.0256 (19)	0.0265 (17)	0.0005 (15)	0.0130 (15)	0.0058 (15)
C35	0.0300 (19)	0.0190 (17)	0.0254 (17)	−0.0025 (14)	0.0118 (15)	0.0021 (14)
C36	0.0235 (17)	0.0198 (16)	0.0193 (15)	−0.0006 (13)	0.0059 (13)	−0.0026 (13)
C37	0.037 (2)	0.036 (2)	0.0319 (19)	−0.0149 (17)	0.0124 (17)	0.0009 (17)
N41	0.0236 (14)	0.0172 (14)	0.0170 (13)	0.0015 (11)	0.0043 (11)	−0.0015 (11)
C42	0.0258 (18)	0.0210 (17)	0.0174 (15)	0.0026 (14)	0.0042 (13)	0.0004 (13)
C43	0.032 (2)	0.035 (2)	0.0190 (16)	−0.0017 (16)	0.0037 (15)	0.0048 (15)
C44	0.0319 (19)	0.0293 (19)	0.0170 (16)	0.0015 (16)	−0.0031 (14)	−0.0021 (15)
C45	0.0207 (17)	0.0224 (17)	0.0237 (16)	0.0009 (14)	0.0000 (14)	−0.0043 (14)
C46	0.0211 (17)	0.0201 (16)	0.0190 (15)	−0.0015 (13)	0.0015 (13)	−0.0037 (13)
C47	0.0253 (18)	0.030 (2)	0.0282 (18)	−0.0005 (15)	−0.0007 (15)	−0.0065 (16)
N51	0.0172 (13)	0.0154 (13)	0.0186 (12)	−0.0024 (11)	−0.0004 (10)	−0.0025 (11)
C52	0.0211 (16)	0.0183 (17)	0.0234 (16)	−0.0006 (13)	0.0020 (13)	0.0021 (14)
C53	0.0302 (19)	0.0169 (17)	0.0341 (19)	0.0002 (14)	0.0028 (15)	−0.0049 (15)
C54	0.031 (2)	0.0236 (19)	0.0345 (19)	0.0043 (15)	0.0093 (16)	−0.0081 (16)
C55	0.0274 (18)	0.0266 (19)	0.0260 (17)	0.0032 (15)	0.0080 (15)	−0.0048 (15)
C56	0.0263 (18)	0.0212 (17)	0.0186 (15)	0.0013 (14)	0.0041 (14)	−0.0001 (14)
C57	0.045 (2)	0.032 (2)	0.042 (2)	0.0077 (18)	0.0222 (19)	−0.0039 (18)
N61	0.0199 (14)	0.0187 (14)	0.0164 (12)	−0.0014 (11)	0.0006 (11)	0.0017 (11)
C62	0.0207 (17)	0.0210 (17)	0.0225 (16)	−0.0010 (14)	0.0017 (13)	0.0024 (14)
C63	0.0261 (19)	0.0174 (16)	0.0375 (19)	0.0005 (14)	0.0060 (16)	0.0047 (15)
C64	0.0274 (19)	0.0206 (18)	0.037 (2)	−0.0035 (15)	0.0058 (16)	0.0113 (16)
C65	0.0236 (17)	0.0243 (17)	0.0202 (15)	−0.0005 (14)	0.0023 (13)	0.0050 (14)
C66	0.0236 (17)	0.0244 (18)	0.0189 (15)	−0.0027 (14)	0.0020 (13)	0.0052 (14)
C67	0.032 (2)	0.032 (2)	0.036 (2)	−0.0054 (16)	0.0149 (17)	0.0083 (17)
C71	0.042 (2)	0.0245 (19)	0.0227 (17)	−0.0045 (16)	−0.0027 (16)	0.0022 (15)
C72	0.0319 (19)	0.035 (2)	0.0190 (16)	−0.0082 (16)	−0.0013 (14)	−0.0009 (15)
C73	0.0299 (19)	0.0261 (18)	0.0243 (17)	0.0000 (15)	0.0052 (15)	0.0015 (15)
C711	0.067 (3)	0.028 (2)	0.0206 (18)	−0.012 (2)	−0.0098 (18)	0.0010 (16)
N711	0.094 (3)	0.043 (2)	0.0292 (18)	−0.027 (2)	−0.008 (2)	0.0089 (17)
C712	0.048 (3)	0.0228 (19)	0.0282 (19)	−0.0002 (18)	−0.0096 (18)	−0.0027 (16)
N712	0.053 (2)	0.042 (2)	0.0389 (19)	0.0116 (19)	−0.0035 (18)	−0.0059 (17)
O721	0.0441 (16)	0.0434 (17)	0.0290 (13)	−0.0046 (13)	0.0143 (12)	0.0063 (12)
C721	0.072 (3)	0.044 (3)	0.031 (2)	−0.008 (2)	0.025 (2)	0.004 (2)
C722	0.092 (4)	0.049 (3)	0.044 (3)	−0.014 (3)	0.034 (3)	−0.005 (2)
C731	0.035 (2)	0.030 (2)	0.0206 (16)	−0.0010 (16)	0.0105 (15)	0.0032 (15)
N731	0.050 (2)	0.0315 (19)	0.0342 (17)	0.0039 (16)	0.0196 (16)	0.0004 (15)
C732	0.034 (2)	0.0223 (18)	0.0273 (18)	0.0003 (15)	0.0078 (16)	0.0020 (15)
N732	0.053 (2)	0.0253 (17)	0.046 (2)	0.0025 (16)	0.0213 (18)	0.0020 (16)
B81	0.043 (3)	0.024 (2)	0.024 (2)	−0.0002 (18)	0.0084 (18)	−0.0013 (17)
F81	0.0558 (15)	0.0291 (12)	0.0258 (10)	0.0012 (10)	0.0094 (10)	0.0062 (9)
F82	0.0552 (15)	0.0262 (12)	0.0370 (12)	0.0000 (10)	0.0056 (11)	0.0002 (10)
F83	0.0625 (16)	0.0371 (13)	0.0258 (11)	−0.0089 (11)	0.0077 (11)	−0.0041 (10)
F84	0.0356 (14)	0.071 (2)	0.0698 (18)	0.0020 (13)	0.0150 (13)	−0.0023 (15)

### Tris(5,5'-dimethyl-2,2'-bipyridine)iron(II) 1,1,3,3-tetracyano-2-ethoxypropenide tetrafluoridoborate (IV) Geometric parameters (Å, º)

**Table:**

Fe1—N31	1.967 (3)	C45—C47	1.503 (5)
Fe1—N21	1.967 (3)	C46—H46	0.9500
Fe1—N11	1.975 (3)	C47—H47A	0.9800
Fe1—N41	1.975 (3)	C47—H47B	0.9800
Fe1—N51	1.976 (3)	C47—H47C	0.9800
Fe1—N61	1.981 (3)	N51—C56	1.339 (4)
N11—C16	1.340 (4)	N51—C52	1.358 (4)
N11—C12	1.357 (4)	C52—C53	1.384 (5)
C12—C13	1.390 (5)	C52—C62	1.466 (5)
C12—C22	1.467 (5)	C53—C54	1.385 (5)
C13—C14	1.387 (5)	C53—H53	0.9500
C13—H13	0.9500	C54—C55	1.392 (5)
C14—C15	1.383 (5)	C54—H54	0.9500
C14—H14	0.9500	C55—C56	1.392 (5)
C15—C16	1.396 (5)	C55—C57	1.505 (5)
C15—C17	1.501 (5)	C56—H56	0.9500
C16—H16	0.9500	C57—H57A	0.9800
C17—H17A	0.9800	C57—H57B	0.9800
C17—H17B	0.9800	C57—H57C	0.9800
C17—H17C	0.9800	N61—C66	1.345 (4)
N21—C26	1.348 (4)	N61—C62	1.360 (4)
N21—C22	1.364 (4)	C62—C63	1.388 (5)
C22—C23	1.381 (5)	C63—C64	1.383 (5)
C23—C24	1.378 (5)	C63—H63	0.9500
C23—H23	0.9500	C64—C65	1.387 (5)
C24—C25	1.401 (5)	C64—H64	0.9500
C24—H24	0.9500	C65—C66	1.382 (5)
C25—C26	1.383 (5)	C65—C67	1.504 (5)
C25—C27	1.500 (5)	C66—H66	0.9500
C26—H26	0.9500	C67—H67A	0.9800
C27—H27A	0.9800	C67—H67B	0.9800
C27—H27B	0.9800	C67—H67C	0.9800
C27—H27C	0.9800	C71—C72	1.403 (6)
N31—C36	1.346 (4)	C71—C711	1.409 (6)
N31—C32	1.362 (4)	C71—C712	1.423 (6)
C32—C33	1.391 (5)	C72—O721	1.353 (5)
C32—C42	1.453 (5)	C72—C73	1.388 (5)
C33—C34	1.385 (5)	C73—C731	1.415 (5)
C33—H33	0.9500	C73—C732	1.422 (5)
C34—C35	1.398 (5)	C711—N711	1.162 (6)
C34—H34	0.9500	C712—N712	1.162 (6)
C35—C36	1.382 (5)	O721—C721	1.467 (5)
C35—C37	1.502 (5)	C721—C722	1.500 (7)
C36—H36	0.9500	C721—H71A	0.9900
C37—H37A	0.9800	C721—H71B	0.9900
C37—H37B	0.9800	C722—H72A	0.9800
C37—H37C	0.9800	C722—H72B	0.9800
N41—C46	1.352 (4)	C722—H72C	0.9800
N41—C42	1.360 (4)	C731—N731	1.156 (5)
C42—C43	1.390 (5)	C732—N732	1.148 (5)
C43—C44	1.391 (5)	B81—F84	1.372 (5)
C43—H43	0.9500	B81—F83	1.391 (5)
C44—C45	1.386 (5)	B81—F81	1.394 (5)
C44—H44	0.9500	B81—F82	1.399 (5)
C45—C46	1.396 (4)		
			
N31—Fe1—N21	92.32 (11)	C45—C44—C43	119.7 (3)
N31—Fe1—N11	94.45 (11)	C45—C44—H44	120.1
N21—Fe1—N11	81.70 (11)	C43—C44—H44	120.1
N31—Fe1—N41	81.39 (11)	C44—C45—C46	117.9 (3)
N21—Fe1—N41	96.80 (11)	C44—C45—C47	122.3 (3)
N11—Fe1—N41	175.54 (11)	C46—C45—C47	119.7 (3)
N31—Fe1—N51	92.18 (11)	N41—C46—C45	123.1 (3)
N21—Fe1—N51	173.64 (11)	N41—C46—H46	118.5
N11—Fe1—N51	93.48 (11)	C45—C46—H46	118.5
N41—Fe1—N51	88.30 (11)	C45—C47—H47A	109.5
N31—Fe1—N61	172.21 (11)	C45—C47—H47B	109.5
N21—Fe1—N61	94.34 (11)	H47A—C47—H47B	109.5
N11—Fe1—N61	90.50 (11)	C45—C47—H47C	109.5
N41—Fe1—N61	93.80 (11)	H47A—C47—H47C	109.5
N51—Fe1—N61	81.50 (11)	H47B—C47—H47C	109.5
C16—N11—C12	118.3 (3)	C56—N51—C52	118.4 (3)
C16—N11—Fe1	126.9 (2)	C56—N51—Fe1	126.0 (2)
C12—N11—Fe1	114.8 (2)	C52—N51—Fe1	115.5 (2)
N11—C12—C13	121.1 (3)	N51—C52—C53	121.2 (3)
N11—C12—C22	114.3 (3)	N51—C52—C62	113.6 (3)
C13—C12—C22	124.6 (3)	C53—C52—C62	125.2 (3)
C14—C13—C12	119.7 (3)	C52—C53—C54	119.9 (3)
C14—C13—H13	120.1	C52—C53—H53	120.1
C12—C13—H13	120.1	C54—C53—H53	120.1
C15—C14—C13	119.7 (3)	C53—C54—C55	119.4 (3)
C15—C14—H14	120.2	C53—C54—H54	120.3
C13—C14—H14	120.2	C55—C54—H54	120.3
C14—C15—C16	117.4 (3)	C54—C55—C56	117.4 (3)
C14—C15—C17	123.4 (3)	C54—C55—C57	123.2 (3)
C16—C15—C17	119.2 (3)	C56—C55—C57	119.4 (3)
N11—C16—C15	123.8 (3)	N51—C56—C55	123.6 (3)
N11—C16—H16	118.1	N51—C56—H56	118.2
C15—C16—H16	118.1	C55—C56—H56	118.2
C15—C17—H17A	109.5	C55—C57—H57A	109.5
C15—C17—H17B	109.5	C55—C57—H57B	109.5
H17A—C17—H17B	109.5	H57A—C57—H57B	109.5
C15—C17—H17C	109.5	C55—C57—H57C	109.5
H17A—C17—H17C	109.5	H57A—C57—H57C	109.5
H17B—C17—H17C	109.5	H57B—C57—H57C	109.5
C26—N21—C22	118.1 (3)	C66—N61—C62	118.4 (3)
C26—N21—Fe1	126.9 (2)	C66—N61—Fe1	127.1 (2)
C22—N21—Fe1	114.9 (2)	C62—N61—Fe1	114.5 (2)
N21—C22—C23	121.2 (3)	N61—C62—C63	121.2 (3)
N21—C22—C12	114.0 (3)	N61—C62—C52	114.9 (3)
C23—C22—C12	124.9 (3)	C63—C62—C52	123.9 (3)
C24—C23—C22	119.9 (3)	C64—C63—C62	119.2 (3)
C24—C23—H23	120.0	C64—C63—H63	120.4
C22—C23—H23	120.0	C62—C63—H63	120.4
C23—C24—C25	119.8 (3)	C63—C64—C65	120.3 (3)
C23—C24—H24	120.1	C63—C64—H64	119.9
C25—C24—H24	120.1	C65—C64—H64	119.9
C26—C25—C24	117.0 (3)	C66—C65—C64	117.3 (3)
C26—C25—C27	121.1 (3)	C66—C65—C67	120.5 (3)
C24—C25—C27	121.8 (3)	C64—C65—C67	122.1 (3)
N21—C26—C25	123.9 (3)	N61—C66—C65	123.7 (3)
N21—C26—H26	118.0	N61—C66—H66	118.2
C25—C26—H26	118.0	C65—C66—H66	118.2
C25—C27—H27A	109.5	C65—C67—H67A	109.5
C25—C27—H27B	109.5	C65—C67—H67B	109.5
H27A—C27—H27B	109.5	H67A—C67—H67B	109.5
C25—C27—H27C	109.5	C65—C67—H67C	109.5
H27A—C27—H27C	109.5	H67A—C67—H67C	109.5
H27B—C27—H27C	109.5	H67B—C67—H67C	109.5
C36—N31—C32	118.4 (3)	C72—C71—C711	121.8 (4)
C36—N31—Fe1	126.5 (2)	C72—C71—C712	121.6 (4)
C32—N31—Fe1	114.8 (2)	C711—C71—C712	116.5 (4)
N31—C32—C33	120.9 (3)	O721—C72—C73	114.0 (3)
N31—C32—C42	113.9 (3)	O721—C72—C71	119.9 (3)
C33—C32—C42	125.2 (3)	C73—C72—C71	126.0 (4)
C34—C33—C32	119.9 (3)	C72—C73—C731	120.1 (3)
C34—C33—H33	120.0	C72—C73—C732	123.7 (3)
C32—C33—H33	120.0	C731—C73—C732	116.1 (3)
C33—C34—C35	119.4 (3)	N711—C711—C71	177.8 (4)
C33—C34—H34	120.3	N712—C712—C71	177.1 (4)
C35—C34—H34	120.3	C72—O721—C721	118.8 (3)
C36—C35—C34	117.5 (3)	O721—C721—C722	106.6 (4)
C36—C35—C37	120.0 (3)	O721—C721—H71A	110.4
C34—C35—C37	122.5 (3)	C722—C721—H71A	110.4
N31—C36—C35	123.9 (3)	O721—C721—H71B	110.4
N31—C36—H36	118.0	C722—C721—H71B	110.4
C35—C36—H36	118.0	H71A—C721—H71B	108.6
C35—C37—H37A	109.5	C721—C722—H72A	109.5
C35—C37—H37B	109.5	C721—C722—H72B	109.5
H37A—C37—H37B	109.5	H72A—C722—H72B	109.5
C35—C37—H37C	109.5	C721—C722—H72C	109.5
H37A—C37—H37C	109.5	H72A—C722—H72C	109.5
H37B—C37—H37C	109.5	H72B—C722—H72C	109.5
C46—N41—C42	118.3 (3)	N731—C731—C73	176.9 (4)
C46—N41—Fe1	127.3 (2)	N732—C732—C73	176.0 (4)
C42—N41—Fe1	114.2 (2)	F84—B81—F83	110.1 (3)
N41—C42—C43	121.5 (3)	F84—B81—F81	110.4 (3)
N41—C42—C32	114.6 (3)	F83—B81—F81	109.3 (3)
C43—C42—C32	123.9 (3)	F84—B81—F82	109.8 (3)
C42—C43—C44	119.3 (3)	F83—B81—F82	108.9 (3)
C42—C43—H43	120.3	F81—B81—F82	108.3 (3)
C44—C43—H43	120.3		
			
C16—N11—C12—C13	−1.8 (5)	N41—C42—C43—C44	1.4 (5)
Fe1—N11—C12—C13	177.4 (3)	C32—C42—C43—C44	−177.9 (3)
C16—N11—C12—C22	176.8 (3)	C42—C43—C44—C45	1.0 (6)
Fe1—N11—C12—C22	−4.0 (4)	C43—C44—C45—C46	−1.6 (5)
N11—C12—C13—C14	1.3 (5)	C43—C44—C45—C47	178.0 (3)
C22—C12—C13—C14	−177.2 (3)	C42—N41—C46—C45	2.4 (5)
C12—C13—C14—C15	0.3 (5)	Fe1—N41—C46—C45	−171.9 (3)
C13—C14—C15—C16	−1.3 (5)	C44—C45—C46—N41	−0.1 (5)
C13—C14—C15—C17	−179.7 (3)	C47—C45—C46—N41	−179.7 (3)
C12—N11—C16—C15	0.8 (5)	C56—N51—C52—C53	−2.4 (5)
Fe1—N11—C16—C15	−178.3 (2)	Fe1—N51—C52—C53	179.5 (3)
C14—C15—C16—N11	0.8 (5)	C56—N51—C52—C62	176.8 (3)
C17—C15—C16—N11	179.2 (3)	Fe1—N51—C52—C62	−1.3 (3)
C26—N21—C22—C23	2.2 (5)	N51—C52—C53—C54	2.6 (5)
Fe1—N21—C22—C23	−175.5 (3)	C62—C52—C53—C54	−176.5 (3)
C26—N21—C22—C12	−177.3 (3)	C52—C53—C54—C55	−0.5 (5)
Fe1—N21—C22—C12	5.0 (4)	C53—C54—C55—C56	−1.6 (5)
N11—C12—C22—N21	−0.7 (4)	C53—C54—C55—C57	178.2 (3)
C13—C12—C22—N21	177.9 (3)	C52—N51—C56—C55	0.2 (5)
N11—C12—C22—C23	179.9 (3)	Fe1—N51—C56—C55	178.1 (2)
C13—C12—C22—C23	−1.5 (5)	C54—C55—C56—N51	1.8 (5)
N21—C22—C23—C24	−1.8 (5)	C57—C55—C56—N51	−178.0 (3)
C12—C22—C23—C24	177.6 (3)	C66—N61—C62—C63	0.0 (5)
C22—C23—C24—C25	0.1 (5)	Fe1—N61—C62—C63	179.1 (3)
C23—C24—C25—C26	1.0 (5)	C66—N61—C62—C52	−178.7 (3)
C23—C24—C25—C27	−178.8 (3)	Fe1—N61—C62—C52	0.5 (3)
C22—N21—C26—C25	−1.0 (5)	N51—C52—C62—N61	0.5 (4)
Fe1—N21—C26—C25	176.3 (2)	C53—C52—C62—N61	179.6 (3)
C24—C25—C26—N21	−0.5 (5)	N51—C52—C62—C63	−178.1 (3)
C27—C25—C26—N21	179.3 (3)	C53—C52—C62—C63	1.0 (5)
C36—N31—C32—C33	0.6 (5)	N61—C62—C63—C64	−0.7 (5)
Fe1—N31—C32—C33	−173.5 (2)	C52—C62—C63—C64	177.8 (3)
C36—N31—C32—C42	−178.9 (3)	C62—C63—C64—C65	0.7 (5)
Fe1—N31—C32—C42	7.0 (3)	C63—C64—C65—C66	0.0 (5)
N31—C32—C33—C34	1.0 (5)	C63—C64—C65—C67	−179.3 (3)
C42—C32—C33—C34	−179.7 (3)	C62—N61—C66—C65	0.8 (5)
C32—C33—C34—C35	−1.7 (5)	Fe1—N61—C66—C65	−178.2 (2)
C33—C34—C35—C36	0.9 (5)	C64—C65—C66—N61	−0.8 (5)
C33—C34—C35—C37	−178.9 (3)	C67—C65—C66—N61	178.5 (3)
C32—N31—C36—C35	−1.4 (5)	C711—C71—C72—O721	−22.7 (5)
Fe1—N31—C36—C35	171.9 (3)	C712—C71—C72—O721	161.7 (3)
C34—C35—C36—N31	0.7 (5)	C711—C71—C72—C73	154.7 (4)
C37—C35—C36—N31	−179.5 (3)	C712—C71—C72—C73	−20.9 (6)
C46—N41—C42—C43	−3.1 (5)	O721—C72—C73—C731	−13.9 (5)
Fe1—N41—C42—C43	172.0 (3)	C71—C72—C73—C731	168.6 (3)
C46—N41—C42—C32	176.3 (3)	O721—C72—C73—C732	162.6 (3)
Fe1—N41—C42—C32	−8.6 (4)	C71—C72—C73—C732	−14.9 (6)
N31—C32—C42—N41	1.1 (4)	C73—C72—O721—C721	136.0 (4)
C33—C32—C42—N41	−178.3 (3)	C71—C72—O721—C721	−46.4 (5)
N31—C32—C42—C43	−179.5 (3)	C72—O721—C721—C722	−133.9 (4)
C33—C32—C42—C43	1.1 (5)		

### Tris(5,5'-dimethyl-2,2'-bipyridine)iron(II) 1,1,3,3-tetracyano-2-ethoxypropenide tetrafluoridoborate (IV) Hydrogen-bond geometry (Å, º)

**Table:**

*D*—H···*A*	*D*—H	H···*A*	*D*···*A*	*D*—H···*A*
C23—H23···F81^i^	0.95	2.38	3.259 (4)	154
C44—H44···N711	0.95	2.58	3.461 (5)	155
C53—H53···F82	0.95	2.40	3.342 (4)	171

Symmetry code: (i) −*x*+3/2, *y*+1/2, −*z*+3/2.

### Tris(5,5'-dimethyl-2,2'-bipyridine)iron(II) 1,1,3,3-tetracyano-2-(ethylsufanyl)propenide tetrafluoridoborate (V) Crystal data

**Table:**

[Fe(C_12_H_12_N_2_)_3_](C_9_H_5_N_4_S)(BF_4_)	*F*(000) = 1856
*M**_r_* = 896.60	*D*_x_ = 1.432 Mg m^−^^3^
Monoclinic, *P*2_1_/*n*	Ga *K*α radiation, λ = 1.34139 Å
*a* = 11.6027 (5) Å	Cell parameters from 9564 reflections
*b* = 25.0774 (10) Å	θ = 3.1–60.7°
*c* = 14.7438 (6) Å	µ = 2.67 mm^−^^1^
β = 104.211 (2)°	*T* = 100 K
*V* = 4158.7 (3) Å^3^	Plate, red
*Z* = 4	0.13 × 0.11 × 0.03 mm

### Tris(5,5'-dimethyl-2,2'-bipyridine)iron(II) 1,1,3,3-tetracyano-2-(ethylsufanyl)propenide tetrafluoridoborate (V) Data collection

**Table:**

Bruker Venture Metaljet diffractometer	9563 independent reflections
Helios MX Mirror Optics monochromator	8430 reflections with *I* > 2σ(*I*)
Detector resolution: 10.24 pixels mm^-1^	*R*_int_ = 0.037
ω and φ scans	θ_max_ = 60.7°, θ_min_ = 3.1°
Absorption correction: multi-scan (SADABS; Bruker, 2014)	*h* = −15→14
*T*_min_ = 0.832, *T*_max_ = 0.923	*k* = −32→32
64342 measured reflections	*l* = −18→19

### Tris(5,5'-dimethyl-2,2'-bipyridine)iron(II) 1,1,3,3-tetracyano-2-(ethylsufanyl)propenide tetrafluoridoborate (V) Refinement

**Table:**

Refinement on *F*^2^	0 restraints
Least-squares matrix: full	Hydrogen site location: inferred from neighbouring sites
*R*[*F*^2^ > 2σ(*F*^2^)] = 0.033	H-atom parameters constrained
*wR*(*F*^2^) = 0.086	*w* = 1/[σ^2^(*F*_o_^2^) + (0.0437*P*)^2^ + 2.086*P*] where *P* = (*F*_o_^2^ + 2*F*_c_^2^)/3
*S* = 1.04	(Δ/σ)_max_ = 0.002
9563 reflections	Δρ_max_ = 0.40 e Å^−^^3^
566 parameters	Δρ_min_ = −0.35 e Å^−^^3^

### Tris(5,5'-dimethyl-2,2'-bipyridine)iron(II) 1,1,3,3-tetracyano-2-(ethylsufanyl)propenide tetrafluoridoborate (V) Special details

**Table:**

Geometry. All esds (except the esd in the dihedral angle between two l.s. planes) are estimated using the full covariance matrix. The cell esds are taken into account individually in the estimation of esds in distances, angles and torsion angles; correlations between esds in cell parameters are only used when they are defined by crystal symmetry. An approximate (isotropic) treatment of cell esds is used for estimating esds involving l.s. planes.

### Tris(5,5'-dimethyl-2,2'-bipyridine)iron(II) 1,1,3,3-tetracyano-2-(ethylsufanyl)propenide tetrafluoridoborate (V) Fractional atomic coordinates and isotropic or equivalent isotropic displacement parameters (Å^2^)

**Table:**

	*x*	*y*	*z*	*U*_iso_*/*U*_eq_	
Fe1	0.71596 (2)	0.66554 (2)	0.52112 (2)	0.01007 (6)	
N11	0.84219 (10)	0.66916 (4)	0.63839 (8)	0.0125 (2)	
C12	0.81638 (12)	0.70018 (5)	0.70612 (9)	0.0138 (3)	
C13	0.89422 (13)	0.70445 (6)	0.79410 (10)	0.0186 (3)	
H13	0.8743	0.7260	0.8410	0.022*	
C14	1.00143 (13)	0.67691 (6)	0.81287 (10)	0.0192 (3)	
H14	1.0547	0.6791	0.8730	0.023*	
C15	1.03034 (12)	0.64624 (6)	0.74317 (10)	0.0168 (3)	
C16	0.94719 (12)	0.64362 (6)	0.65714 (10)	0.0145 (3)	
H16	0.9658	0.6226	0.6091	0.017*	
C17	1.14363 (13)	0.61489 (7)	0.75751 (11)	0.0226 (3)	
H17A	1.2015	0.6284	0.8128	0.027*	
H17B	1.1760	0.6186	0.7024	0.027*	
H17C	1.1274	0.5772	0.7667	0.027*	
N21	0.64337 (10)	0.71929 (4)	0.58661 (8)	0.0123 (2)	
C22	0.70287 (12)	0.72867 (5)	0.67683 (9)	0.0138 (3)	
C23	0.65779 (13)	0.76269 (6)	0.73392 (10)	0.0195 (3)	
H23	0.6992	0.7680	0.7973	0.023*	
C24	0.55151 (13)	0.78882 (6)	0.69702 (11)	0.0215 (3)	
H24	0.5196	0.8121	0.7355	0.026*	
C25	0.49103 (13)	0.78129 (6)	0.60398 (11)	0.0178 (3)	
C26	0.54064 (12)	0.74543 (5)	0.55225 (10)	0.0146 (3)	
H26	0.4996	0.7390	0.4891	0.017*	
C27	0.37654 (14)	0.80949 (7)	0.56091 (12)	0.0255 (3)	
H27A	0.3317	0.7890	0.5072	0.031*	
H27B	0.3938	0.8450	0.5400	0.031*	
H27C	0.3293	0.8129	0.6074	0.031*	
N31	0.78708 (10)	0.71997 (5)	0.45662 (8)	0.0120 (2)	
C32	0.73629 (12)	0.72487 (5)	0.36348 (9)	0.0140 (3)	
C33	0.78629 (13)	0.75674 (6)	0.30626 (10)	0.0183 (3)	
H33	0.7502	0.7592	0.2412	0.022*	
C34	0.88940 (13)	0.78486 (6)	0.34503 (10)	0.0196 (3)	
H34	0.9254	0.8062	0.3064	0.023*	
C35	0.94000 (13)	0.78164 (6)	0.44087 (10)	0.0182 (3)	
C36	0.88539 (12)	0.74847 (6)	0.49304 (10)	0.0147 (3)	
H36	0.9196	0.7458	0.5584	0.018*	
C37	1.04908 (16)	0.81237 (8)	0.48835 (12)	0.0326 (4)	
H37A	1.1002	0.7900	0.5363	0.039*	
H37B	1.0258	0.8443	0.5178	0.039*	
H37C	1.0926	0.8229	0.4420	0.039*	
N41	0.59339 (10)	0.66703 (4)	0.40162 (8)	0.0124 (2)	
C42	0.62642 (12)	0.69393 (6)	0.33208 (9)	0.0145 (3)	
C43	0.55766 (13)	0.69260 (6)	0.24049 (10)	0.0196 (3)	
H43	0.5833	0.7110	0.1926	0.024*	
C44	0.45179 (14)	0.66446 (6)	0.21930 (10)	0.0202 (3)	
H44	0.4051	0.6628	0.1566	0.024*	
C45	0.41424 (12)	0.63861 (6)	0.29061 (10)	0.0162 (3)	
C46	0.48861 (12)	0.64090 (5)	0.38047 (9)	0.0139 (3)	
H46	0.4642	0.6231	0.4295	0.017*	
C47	0.29815 (13)	0.60906 (6)	0.27283 (11)	0.0205 (3)	
H47A	0.2709	0.6076	0.3306	0.025*	
H47B	0.3091	0.5727	0.2520	0.025*	
H47C	0.2388	0.6276	0.2243	0.025*	
N51	0.79439 (10)	0.60732 (5)	0.46979 (8)	0.0126 (2)	
C52	0.76141 (12)	0.55728 (6)	0.48779 (9)	0.0140 (3)	
C53	0.80893 (13)	0.51254 (6)	0.45444 (10)	0.0181 (3)	
H53	0.7836	0.4778	0.4666	0.022*	
C54	0.89356 (13)	0.51915 (6)	0.40334 (10)	0.0188 (3)	
H54	0.9260	0.4889	0.3797	0.023*	
C55	0.93094 (12)	0.57018 (6)	0.38671 (10)	0.0166 (3)	
C56	0.87784 (12)	0.61264 (6)	0.42123 (9)	0.0146 (3)	
H56	0.9020	0.6477	0.4098	0.018*	
C57	1.02484 (14)	0.58081 (6)	0.33517 (11)	0.0228 (3)	
H57A	1.0881	0.5542	0.3530	0.027*	
H57B	1.0581	0.6165	0.3511	0.027*	
H57C	0.9897	0.5787	0.2677	0.027*	
N61	0.64235 (10)	0.60455 (5)	0.57084 (8)	0.0129 (2)	
C62	0.67602 (12)	0.55573 (6)	0.54650 (10)	0.0146 (3)	
C63	0.63323 (13)	0.50912 (6)	0.57694 (11)	0.0190 (3)	
H63	0.6578	0.4754	0.5593	0.023*	
C64	0.55394 (13)	0.51249 (6)	0.63357 (11)	0.0201 (3)	
H64	0.5243	0.4809	0.6552	0.024*	
C65	0.51805 (12)	0.56191 (6)	0.65863 (10)	0.0170 (3)	
C66	0.56466 (12)	0.60678 (6)	0.62507 (9)	0.0150 (3)	
H66	0.5403	0.6409	0.6414	0.018*	
C67	0.43329 (13)	0.56785 (6)	0.72049 (11)	0.0216 (3)	
H67A	0.3751	0.5388	0.7078	0.026*	
H67B	0.3919	0.6021	0.7077	0.026*	
H67C	0.4777	0.5665	0.7862	0.026*	
C71	0.17682 (14)	0.54214 (6)	0.06097 (10)	0.0205 (3)	
C72	0.24020 (13)	0.49426 (6)	0.08130 (10)	0.0190 (3)	
C73	0.21931 (13)	0.45510 (6)	0.14303 (10)	0.0186 (3)	
C711	0.22519 (15)	0.58724 (7)	0.02518 (11)	0.0266 (4)	
N711	0.26243 (16)	0.62531 (7)	−0.00091 (11)	0.0405 (4)	
C712	0.06257 (14)	0.54985 (6)	0.07816 (10)	0.0221 (3)	
N712	−0.03085 (13)	0.55716 (6)	0.08989 (10)	0.0293 (3)	
S721	0.36106 (3)	0.48122 (2)	0.03181 (3)	0.02750 (10)	
C721	0.30215 (15)	0.49586 (7)	−0.09246 (11)	0.0260 (3)	
H71A	0.2154	0.5023	−0.1054	0.031*	
H71B	0.3403	0.5285	−0.1094	0.031*	
C722	0.32656 (19)	0.44924 (8)	−0.15030 (13)	0.0365 (4)	
H72A	0.2869	0.4173	−0.1345	0.044*	
H72B	0.4124	0.4429	−0.1369	0.044*	
H72C	0.2962	0.4573	−0.2169	0.044*	
C731	0.27242 (13)	0.40366 (6)	0.14903 (10)	0.0204 (3)	
N731	0.31371 (13)	0.36176 (6)	0.16050 (9)	0.0263 (3)	
C732	0.14904 (14)	0.46374 (6)	0.20863 (11)	0.0206 (3)	
N732	0.09621 (14)	0.46811 (5)	0.26472 (10)	0.0282 (3)	
B81	0.75542 (17)	0.33470 (7)	0.47638 (12)	0.0214 (3)	
F81	0.72973 (10)	0.30477 (4)	0.54880 (7)	0.0319 (2)	
F82	0.72315 (10)	0.38754 (4)	0.48730 (7)	0.0336 (2)	
F83	0.68953 (9)	0.31552 (4)	0.39074 (6)	0.0301 (2)	
F84	0.87496 (10)	0.33149 (5)	0.48029 (9)	0.0499 (3)	

### Tris(5,5'-dimethyl-2,2'-bipyridine)iron(II) 1,1,3,3-tetracyano-2-(ethylsufanyl)propenide tetrafluoridoborate (V) Atomic displacement parameters (Å^2^)

**Table:**

	*U*^11^	*U*^22^	*U*^33^	*U*^12^	*U*^13^	*U*^23^
Fe1	0.01110 (10)	0.00990 (10)	0.00925 (10)	−0.00014 (7)	0.00259 (7)	0.00031 (7)
N11	0.0142 (5)	0.0119 (5)	0.0118 (5)	−0.0016 (4)	0.0038 (4)	0.0016 (4)
C12	0.0144 (6)	0.0139 (6)	0.0135 (6)	−0.0030 (5)	0.0041 (5)	0.0006 (5)
C13	0.0195 (7)	0.0220 (7)	0.0142 (7)	−0.0030 (6)	0.0041 (5)	−0.0020 (5)
C14	0.0186 (7)	0.0228 (8)	0.0137 (7)	−0.0034 (6)	−0.0009 (5)	0.0012 (5)
C15	0.0152 (6)	0.0174 (7)	0.0168 (7)	−0.0020 (5)	0.0020 (5)	0.0041 (5)
C16	0.0145 (6)	0.0143 (7)	0.0142 (6)	−0.0002 (5)	0.0027 (5)	0.0019 (5)
C17	0.0177 (7)	0.0260 (8)	0.0212 (7)	0.0037 (6)	−0.0006 (6)	0.0017 (6)
N21	0.0137 (5)	0.0115 (5)	0.0120 (5)	−0.0024 (4)	0.0033 (4)	0.0006 (4)
C22	0.0145 (6)	0.0138 (6)	0.0134 (6)	−0.0032 (5)	0.0039 (5)	−0.0004 (5)
C23	0.0200 (7)	0.0222 (8)	0.0165 (7)	−0.0022 (6)	0.0046 (6)	−0.0059 (6)
C24	0.0208 (7)	0.0211 (8)	0.0240 (8)	−0.0003 (6)	0.0082 (6)	−0.0089 (6)
C25	0.0164 (7)	0.0137 (7)	0.0237 (7)	−0.0007 (5)	0.0061 (6)	−0.0025 (5)
C26	0.0152 (6)	0.0129 (6)	0.0155 (6)	−0.0001 (5)	0.0036 (5)	0.0002 (5)
C27	0.0199 (7)	0.0210 (8)	0.0341 (9)	0.0065 (6)	0.0036 (6)	−0.0055 (7)
N31	0.0134 (5)	0.0113 (5)	0.0122 (5)	0.0016 (4)	0.0047 (4)	0.0002 (4)
C32	0.0161 (6)	0.0133 (6)	0.0130 (6)	0.0021 (5)	0.0043 (5)	0.0006 (5)
C33	0.0213 (7)	0.0198 (7)	0.0146 (6)	0.0016 (6)	0.0059 (5)	0.0036 (5)
C34	0.0222 (7)	0.0194 (7)	0.0198 (7)	−0.0017 (6)	0.0105 (6)	0.0044 (6)
C35	0.0181 (7)	0.0182 (7)	0.0202 (7)	−0.0026 (5)	0.0082 (6)	−0.0001 (6)
C36	0.0157 (6)	0.0146 (7)	0.0143 (6)	−0.0008 (5)	0.0048 (5)	−0.0010 (5)
C37	0.0297 (9)	0.0429 (11)	0.0261 (8)	−0.0218 (8)	0.0087 (7)	0.0005 (7)
N41	0.0146 (5)	0.0113 (5)	0.0117 (5)	0.0012 (4)	0.0038 (4)	−0.0004 (4)
C42	0.0163 (6)	0.0145 (7)	0.0131 (6)	0.0016 (5)	0.0045 (5)	0.0009 (5)
C43	0.0225 (7)	0.0236 (8)	0.0123 (6)	−0.0016 (6)	0.0035 (5)	0.0017 (5)
C44	0.0221 (7)	0.0235 (8)	0.0128 (6)	0.0000 (6)	−0.0001 (5)	−0.0008 (5)
C45	0.0169 (6)	0.0145 (7)	0.0161 (7)	0.0012 (5)	0.0020 (5)	−0.0029 (5)
C46	0.0150 (6)	0.0127 (6)	0.0141 (6)	0.0004 (5)	0.0034 (5)	−0.0005 (5)
C47	0.0177 (7)	0.0214 (8)	0.0195 (7)	−0.0030 (6)	−0.0006 (6)	−0.0026 (6)
N51	0.0134 (5)	0.0128 (6)	0.0109 (5)	−0.0001 (4)	0.0018 (4)	−0.0003 (4)
C52	0.0137 (6)	0.0127 (7)	0.0144 (6)	−0.0006 (5)	0.0009 (5)	0.0004 (5)
C53	0.0203 (7)	0.0120 (7)	0.0218 (7)	0.0002 (5)	0.0047 (6)	−0.0008 (5)
C54	0.0209 (7)	0.0150 (7)	0.0204 (7)	0.0032 (5)	0.0049 (6)	−0.0039 (5)
C55	0.0163 (6)	0.0186 (7)	0.0145 (6)	0.0022 (5)	0.0032 (5)	−0.0012 (5)
C56	0.0168 (6)	0.0141 (7)	0.0133 (6)	0.0001 (5)	0.0043 (5)	0.0008 (5)
C57	0.0254 (8)	0.0219 (8)	0.0252 (8)	0.0033 (6)	0.0141 (6)	−0.0015 (6)
N61	0.0130 (5)	0.0137 (6)	0.0111 (5)	−0.0003 (4)	0.0014 (4)	0.0010 (4)
C62	0.0149 (6)	0.0133 (7)	0.0148 (6)	0.0001 (5)	0.0021 (5)	0.0006 (5)
C63	0.0191 (7)	0.0139 (7)	0.0240 (7)	0.0001 (5)	0.0053 (6)	0.0019 (5)
C64	0.0190 (7)	0.0163 (7)	0.0252 (8)	−0.0024 (6)	0.0058 (6)	0.0064 (6)
C65	0.0153 (6)	0.0199 (7)	0.0153 (6)	−0.0018 (5)	0.0029 (5)	0.0033 (5)
C66	0.0155 (6)	0.0159 (7)	0.0135 (6)	−0.0003 (5)	0.0033 (5)	0.0013 (5)
C67	0.0202 (7)	0.0248 (8)	0.0220 (7)	−0.0021 (6)	0.0096 (6)	0.0053 (6)
C71	0.0224 (7)	0.0222 (8)	0.0148 (7)	−0.0071 (6)	0.0007 (6)	0.0011 (6)
C72	0.0164 (7)	0.0257 (8)	0.0133 (6)	−0.0063 (6)	0.0008 (5)	0.0004 (6)
C73	0.0203 (7)	0.0202 (7)	0.0158 (7)	−0.0022 (6)	0.0052 (6)	0.0007 (5)
C711	0.0321 (9)	0.0266 (9)	0.0167 (7)	−0.0103 (7)	−0.0023 (6)	0.0013 (6)
N711	0.0542 (10)	0.0360 (9)	0.0254 (8)	−0.0223 (8)	−0.0012 (7)	0.0058 (6)
C712	0.0288 (8)	0.0179 (7)	0.0167 (7)	−0.0032 (6)	−0.0001 (6)	−0.0013 (6)
N712	0.0296 (8)	0.0286 (8)	0.0274 (7)	0.0027 (6)	0.0026 (6)	−0.0028 (6)
S721	0.01711 (18)	0.0465 (3)	0.01950 (18)	0.00009 (16)	0.00564 (14)	0.01157 (16)
C721	0.0242 (8)	0.0375 (9)	0.0170 (7)	−0.0022 (7)	0.0063 (6)	0.0087 (6)
C722	0.0457 (11)	0.0358 (10)	0.0267 (9)	−0.0070 (8)	0.0067 (8)	0.0018 (7)
C731	0.0232 (7)	0.0255 (8)	0.0145 (7)	−0.0035 (6)	0.0082 (6)	−0.0004 (6)
N731	0.0335 (7)	0.0262 (7)	0.0236 (7)	0.0018 (6)	0.0152 (6)	0.0007 (5)
C732	0.0274 (8)	0.0140 (7)	0.0212 (7)	−0.0025 (6)	0.0076 (6)	0.0012 (6)
N732	0.0427 (8)	0.0181 (7)	0.0306 (7)	−0.0004 (6)	0.0218 (7)	0.0012 (6)
B81	0.0234 (8)	0.0215 (9)	0.0192 (8)	−0.0014 (7)	0.0051 (7)	−0.0006 (6)
F81	0.0494 (6)	0.0259 (5)	0.0207 (5)	−0.0017 (4)	0.0093 (4)	0.0037 (4)
F82	0.0488 (6)	0.0187 (5)	0.0299 (5)	0.0003 (4)	0.0029 (4)	−0.0007 (4)
F83	0.0417 (6)	0.0282 (5)	0.0190 (4)	−0.0062 (4)	0.0046 (4)	−0.0031 (4)
F84	0.0238 (5)	0.0716 (9)	0.0564 (8)	−0.0013 (5)	0.0137 (5)	−0.0088 (6)

### Tris(5,5'-dimethyl-2,2'-bipyridine)iron(II) 1,1,3,3-tetracyano-2-(ethylsufanyl)propenide tetrafluoridoborate (V) Geometric parameters (Å, º)

**Table:**

Fe1—N31	1.9579 (12)	C45—C47	1.503 (2)
Fe1—N21	1.9642 (12)	C46—H46	0.9500
Fe1—N51	1.9673 (12)	C47—H47A	0.9800
Fe1—N41	1.9743 (12)	C47—H47B	0.9800
Fe1—N11	1.9747 (12)	C47—H47C	0.9800
Fe1—N61	1.9782 (12)	N51—C56	1.3448 (18)
N11—C16	1.3437 (18)	N51—C52	1.3570 (18)
N11—C12	1.3560 (18)	C52—C53	1.392 (2)
C12—C13	1.3909 (19)	C52—C62	1.467 (2)
C12—C22	1.4671 (19)	C53—C54	1.387 (2)
C13—C14	1.390 (2)	C53—H53	0.9500
C13—H13	0.9500	C54—C55	1.392 (2)
C14—C15	1.389 (2)	C54—H54	0.9500
C14—H14	0.9500	C55—C56	1.388 (2)
C15—C16	1.3939 (19)	C55—C57	1.497 (2)
C15—C17	1.501 (2)	C56—H56	0.9500
C16—H16	0.9500	C57—H57A	0.9800
C17—H17A	0.9800	C57—H57B	0.9800
C17—H17B	0.9800	C57—H57C	0.9800
C17—H17C	0.9800	N61—C66	1.3450 (18)
N21—C26	1.3456 (18)	N61—C62	1.3598 (18)
N21—C22	1.3603 (17)	C62—C63	1.387 (2)
C22—C23	1.388 (2)	C63—C64	1.388 (2)
C23—C24	1.384 (2)	C63—H63	0.9500
C23—H23	0.9500	C64—C65	1.386 (2)
C24—C25	1.392 (2)	C64—H64	0.9500
C24—H24	0.9500	C65—C66	1.391 (2)
C25—C26	1.392 (2)	C65—C67	1.504 (2)
C25—C27	1.501 (2)	C66—H66	0.9500
C26—H26	0.9500	C67—H67A	0.9800
C27—H27A	0.9800	C67—H67B	0.9800
C27—H27B	0.9800	C67—H67C	0.9800
C27—H27C	0.9800	C71—C72	1.401 (2)
N31—C36	1.3415 (18)	C71—C711	1.421 (2)
N31—C32	1.3605 (17)	C71—C712	1.422 (2)
C32—C33	1.3882 (19)	C72—C73	1.400 (2)
C32—C42	1.4668 (19)	C72—S721	1.7630 (16)
C33—C34	1.386 (2)	C73—C731	1.423 (2)
C33—H33	0.9500	C73—C732	1.426 (2)
C34—C35	1.393 (2)	C711—N711	1.152 (2)
C34—H34	0.9500	C712—N712	1.154 (2)
C35—C36	1.387 (2)	S721—C721	1.8287 (16)
C35—C37	1.500 (2)	C721—C722	1.514 (3)
C36—H36	0.9500	C721—H71A	0.9900
C37—H37A	0.9800	C721—H71B	0.9900
C37—H37B	0.9800	C722—H72A	0.9800
C37—H37C	0.9800	C722—H72B	0.9800
N41—C46	1.3485 (18)	C722—H72C	0.9800
N41—C42	1.3588 (17)	C731—N731	1.150 (2)
C42—C43	1.3900 (19)	C732—N732	1.149 (2)
C43—C44	1.384 (2)	B81—F84	1.376 (2)
C43—H43	0.9500	B81—F83	1.391 (2)
C44—C45	1.393 (2)	B81—F81	1.396 (2)
C44—H44	0.9500	B81—F82	1.397 (2)
C45—C46	1.3935 (19)		
			
N31—Fe1—N21	92.47 (5)	C43—C44—C45	119.44 (13)
N31—Fe1—N51	92.33 (5)	C43—C44—H44	120.3
N21—Fe1—N51	173.24 (5)	C45—C44—H44	120.3
N31—Fe1—N41	81.40 (5)	C44—C45—C46	117.59 (13)
N21—Fe1—N41	97.11 (5)	C44—C45—C47	122.06 (13)
N51—Fe1—N41	88.32 (5)	C46—C45—C47	120.35 (13)
N31—Fe1—N11	94.75 (5)	N41—C46—C45	123.54 (13)
N21—Fe1—N11	81.57 (5)	N41—C46—H46	118.2
N51—Fe1—N11	93.28 (5)	C45—C46—H46	118.2
N41—Fe1—N11	175.90 (5)	C45—C47—H47A	109.5
N31—Fe1—N61	172.19 (5)	C45—C47—H47B	109.5
N21—Fe1—N61	94.14 (5)	H47A—C47—H47B	109.5
N51—Fe1—N61	81.45 (5)	C45—C47—H47C	109.5
N41—Fe1—N61	93.63 (5)	H47A—C47—H47C	109.5
N11—Fe1—N61	90.34 (5)	H47B—C47—H47C	109.5
C16—N11—C12	118.35 (12)	C56—N51—C52	118.05 (12)
C16—N11—Fe1	126.75 (9)	C56—N51—Fe1	126.35 (10)
C12—N11—Fe1	114.88 (9)	C52—N51—Fe1	115.58 (9)
N11—C12—C13	121.38 (13)	N51—C52—C53	121.41 (13)
N11—C12—C22	114.03 (12)	N51—C52—C62	113.79 (12)
C13—C12—C22	124.58 (13)	C53—C52—C62	124.77 (13)
C14—C13—C12	119.47 (13)	C54—C53—C52	119.36 (13)
C14—C13—H13	120.3	C54—C53—H53	120.3
C12—C13—H13	120.3	C52—C53—H53	120.3
C15—C14—C13	119.65 (13)	C53—C54—C55	119.89 (13)
C15—C14—H14	120.2	C53—C54—H54	120.1
C13—C14—H14	120.2	C55—C54—H54	120.1
C14—C15—C16	117.41 (13)	C56—C55—C54	117.07 (13)
C14—C15—C17	123.22 (13)	C56—C55—C57	119.57 (13)
C16—C15—C17	119.34 (13)	C54—C55—C57	123.36 (13)
N11—C16—C15	123.69 (13)	N51—C56—C55	124.17 (13)
N11—C16—H16	118.2	N51—C56—H56	117.9
C15—C16—H16	118.2	C55—C56—H56	117.9
C15—C17—H17A	109.5	C55—C57—H57A	109.5
C15—C17—H17B	109.5	C55—C57—H57B	109.5
H17A—C17—H17B	109.5	H57A—C57—H57B	109.5
C15—C17—H17C	109.5	C55—C57—H57C	109.5
H17A—C17—H17C	109.5	H57A—C57—H57C	109.5
H17B—C17—H17C	109.5	H57B—C57—H57C	109.5
C26—N21—C22	118.28 (12)	C66—N61—C62	118.19 (12)
C26—N21—Fe1	126.62 (9)	C66—N61—Fe1	126.97 (10)
C22—N21—Fe1	115.05 (9)	C62—N61—Fe1	114.84 (9)
N21—C22—C23	121.54 (13)	N61—C62—C63	121.63 (13)
N21—C22—C12	114.02 (12)	N61—C62—C52	114.28 (12)
C23—C22—C12	124.44 (12)	C63—C62—C52	124.08 (13)
C24—C23—C22	118.98 (13)	C62—C63—C64	119.08 (14)
C24—C23—H23	120.5	C62—C63—H63	120.5
C22—C23—H23	120.5	C64—C63—H63	120.5
C23—C24—C25	120.52 (14)	C65—C64—C63	120.10 (13)
C23—C24—H24	119.7	C65—C64—H64	120.0
C25—C24—H24	119.7	C63—C64—H64	120.0
C26—C25—C24	116.85 (13)	C64—C65—C66	117.39 (13)
C26—C25—C27	120.97 (13)	C64—C65—C67	122.29 (13)
C24—C25—C27	122.18 (13)	C66—C65—C67	120.31 (13)
N21—C26—C25	123.76 (13)	N61—C66—C65	123.61 (13)
N21—C26—H26	118.1	N61—C66—H66	118.2
C25—C26—H26	118.1	C65—C66—H66	118.2
C25—C27—H27A	109.5	C65—C67—H67A	109.5
C25—C27—H27B	109.5	C65—C67—H67B	109.5
H27A—C27—H27B	109.5	H67A—C67—H67B	109.5
C25—C27—H27C	109.5	C65—C67—H67C	109.5
H27A—C27—H27C	109.5	H67A—C67—H67C	109.5
H27B—C27—H27C	109.5	H67B—C67—H67C	109.5
C36—N31—C32	118.09 (12)	C72—C71—C711	121.81 (15)
C36—N31—Fe1	126.54 (9)	C72—C71—C712	122.78 (14)
C32—N31—Fe1	115.06 (9)	C711—C71—C712	115.36 (15)
N31—C32—C33	121.56 (13)	C73—C72—C71	125.07 (14)
N31—C32—C42	113.47 (12)	C73—C72—S721	114.91 (12)
C33—C32—C42	124.97 (12)	C71—C72—S721	119.96 (11)
C34—C33—C32	119.32 (13)	C72—C73—C731	122.34 (14)
C34—C33—H33	120.3	C72—C73—C732	123.60 (14)
C32—C33—H33	120.3	C731—C73—C732	113.97 (13)
C33—C34—C35	119.68 (13)	N711—C711—C71	176.7 (2)
C33—C34—H34	120.2	N712—C712—C71	177.96 (18)
C35—C34—H34	120.2	C72—S721—C721	103.75 (7)
C36—C35—C34	117.46 (13)	C722—C721—S721	109.49 (12)
C36—C35—C37	119.81 (13)	C722—C721—H71A	109.8
C34—C35—C37	122.73 (13)	S721—C721—H71A	109.8
N31—C36—C35	123.83 (13)	C722—C721—H71B	109.8
N31—C36—H36	118.1	S721—C721—H71B	109.8
C35—C36—H36	118.1	H71A—C721—H71B	108.2
C35—C37—H37A	109.5	C721—C722—H72A	109.5
C35—C37—H37B	109.5	C721—C722—H72B	109.5
H37A—C37—H37B	109.5	H72A—C722—H72B	109.5
C35—C37—H37C	109.5	C721—C722—H72C	109.5
H37A—C37—H37C	109.5	H72A—C722—H72C	109.5
H37B—C37—H37C	109.5	H72B—C722—H72C	109.5
C46—N41—C42	118.17 (12)	N731—C731—C73	174.81 (16)
C46—N41—Fe1	127.46 (9)	N732—C732—C73	175.72 (17)
C42—N41—Fe1	114.08 (9)	F84—B81—F83	109.95 (14)
N41—C42—C43	121.34 (13)	F84—B81—F81	109.80 (14)
N41—C42—C32	114.13 (12)	F83—B81—F81	109.59 (14)
C43—C42—C32	124.50 (13)	F84—B81—F82	110.27 (14)
C44—C43—C42	119.83 (13)	F83—B81—F82	109.23 (14)
C44—C43—H43	120.1	F81—B81—F82	107.96 (13)
C42—C43—H43	120.1		
			
C16—N11—C12—C13	−2.03 (19)	N41—C42—C43—C44	1.6 (2)
Fe1—N11—C12—C13	176.76 (11)	C32—C42—C43—C44	−176.32 (14)
C16—N11—C12—C22	176.66 (12)	C42—C43—C44—C45	1.3 (2)
Fe1—N11—C12—C22	−4.55 (15)	C43—C44—C45—C46	−2.3 (2)
N11—C12—C13—C14	0.8 (2)	C43—C44—C45—C47	177.76 (14)
C22—C12—C13—C14	−177.75 (13)	C42—N41—C46—C45	2.1 (2)
C12—C13—C14—C15	1.1 (2)	Fe1—N41—C46—C45	−171.23 (10)
C13—C14—C15—C16	−1.6 (2)	C44—C45—C46—N41	0.6 (2)
C13—C14—C15—C17	−179.51 (14)	C47—C45—C46—N41	−179.42 (13)
C12—N11—C16—C15	1.5 (2)	C56—N51—C52—C53	−2.25 (19)
Fe1—N11—C16—C15	−177.17 (10)	Fe1—N51—C52—C53	179.12 (10)
C14—C15—C16—N11	0.4 (2)	C56—N51—C52—C62	176.07 (12)
C17—C15—C16—N11	178.34 (13)	Fe1—N51—C52—C62	−2.56 (15)
C26—N21—C22—C23	2.4 (2)	N51—C52—C53—C54	1.3 (2)
Fe1—N21—C22—C23	−175.25 (11)	C62—C52—C53—C54	−176.79 (13)
C26—N21—C22—C12	−177.28 (12)	C52—C53—C54—C55	0.6 (2)
Fe1—N21—C22—C12	5.12 (15)	C53—C54—C55—C56	−1.5 (2)
N11—C12—C22—N21	−0.35 (17)	C53—C54—C55—C57	177.80 (14)
C13—C12—C22—N21	178.30 (13)	C52—N51—C56—C55	1.3 (2)
N11—C12—C22—C23	−179.97 (13)	Fe1—N51—C56—C55	179.74 (10)
C13—C12—C22—C23	−1.3 (2)	C54—C55—C56—N51	0.6 (2)
N21—C22—C23—C24	−2.0 (2)	C57—C55—C56—N51	−178.75 (13)
C12—C22—C23—C24	177.64 (14)	C66—N61—C62—C63	−0.66 (19)
C22—C23—C24—C25	−0.3 (2)	Fe1—N61—C62—C63	179.17 (11)
C23—C24—C25—C26	2.0 (2)	C66—N61—C62—C52	−179.66 (12)
C23—C24—C25—C27	−179.05 (15)	Fe1—N61—C62—C52	0.17 (15)
C22—N21—C26—C25	−0.5 (2)	N51—C52—C62—N61	1.55 (17)
Fe1—N21—C26—C25	176.76 (11)	C53—C52—C62—N61	179.80 (13)
C24—C25—C26—N21	−1.6 (2)	N51—C52—C62—C63	−177.42 (13)
C27—C25—C26—N21	179.42 (13)	C53—C52—C62—C63	0.8 (2)
C36—N31—C32—C33	2.4 (2)	N61—C62—C63—C64	0.1 (2)
Fe1—N31—C32—C33	−171.60 (11)	C52—C62—C63—C64	178.99 (13)
C36—N31—C32—C42	−177.30 (12)	C62—C63—C64—C65	0.3 (2)
Fe1—N31—C32—C42	8.70 (15)	C63—C64—C65—C66	−0.1 (2)
N31—C32—C33—C34	−1.0 (2)	C63—C64—C65—C67	−179.42 (14)
C42—C32—C33—C34	178.70 (13)	C62—N61—C66—C65	0.9 (2)
C32—C33—C34—C35	−1.3 (2)	Fe1—N61—C66—C65	−178.94 (10)
C33—C34—C35—C36	2.0 (2)	C64—C65—C66—N61	−0.5 (2)
C33—C34—C35—C37	−177.85 (16)	C67—C65—C66—N61	178.83 (13)
C32—N31—C36—C35	−1.7 (2)	C711—C71—C72—C73	157.89 (15)
Fe1—N31—C36—C35	171.57 (11)	C712—C71—C72—C73	−19.4 (2)
C34—C35—C36—N31	−0.5 (2)	C711—C71—C72—S721	−19.0 (2)
C37—C35—C36—N31	179.32 (15)	C712—C71—C72—S721	163.71 (12)
C46—N41—C42—C43	−3.2 (2)	C71—C72—C73—C731	169.13 (14)
Fe1—N41—C42—C43	171.02 (11)	S721—C72—C73—C731	−13.85 (19)
C46—N41—C42—C32	174.89 (12)	C71—C72—C73—C732	−14.4 (2)
Fe1—N41—C42—C32	−10.90 (15)	S721—C72—C73—C732	162.61 (12)
N31—C32—C42—N41	1.54 (17)	C73—C72—S721—C721	133.85 (12)
C33—C32—C42—N41	−178.15 (13)	C71—C72—S721—C721	−48.97 (14)
N31—C32—C42—C43	179.55 (13)	C72—S721—C721—C722	−128.88 (12)
C33—C32—C42—C43	−0.1 (2)		

### Tris(5,5'-dimethyl-2,2'-bipyridine)iron(II) 1,1,3,3-tetracyano-2-(ethylsufanyl)propenide tetrafluoridoborate (V) Hydrogen-bond geometry (Å, º)

**Table:**

*D*—H···*A*	*D*—H	H···*A*	*D*···*A*	*D*—H···*A*
C23—H23···F81^i^	0.95	2.40	3.3206 (18)	163
C44—H44···N711	0.95	2.67	3.582 (2)	161
C53—H53···F82	0.95	2.41	3.3598 (18)	176

Symmetry code: (i) −*x*+3/2, *y*+1/2, −*z*+3/2.

### Tris(5,5'-dimethyl-2,2'-bipyridine)iron(II) 1,1,3,3-tetracyano-2-propoxypropenide tetrafluoridoborate (VI) Crystal data

**Table:**

[Fe(C_12_H_12_N_2_)_3_](C_10_H_7_N_4_O)(BF_4_)	*Z* = 2
*M**_r_* = 894.56	*F*(000) = 928
Triclinic, *P*1	*D*_x_ = 1.424 Mg m^−^^3^
*a* = 11.6246 (5) Å	Ga *K*α radiation, λ = 1.34139 Å
*b* = 14.2404 (6) Å	Cell parameters from 9590 reflections
*c* = 14.3224 (6) Å	θ = 3.0–60.8°
α = 65.340 (2)°	µ = 2.37 mm^−^^1^
β = 76.040 (3)°	*T* = 100 K
γ = 87.571 (3)°	Block, orange
*V* = 2086.49 (16) Å^3^	0.06 × 0.03 × 0.03 mm

### Tris(5,5'-dimethyl-2,2'-bipyridine)iron(II) 1,1,3,3-tetracyano-2-propoxypropenide tetrafluoridoborate (VI) Data collection

**Table:**

Bruker Venture Metaljet diffractometer	9584 independent reflections
Helios MX Mirror Optics monochromator	7914 reflections with *I* > 2σ(*I*)
Detector resolution: 10.24 pixels mm^-1^	*R*_int_ = 0.052
ω and φ scans	θ_max_ = 60.8°, θ_min_ = 3.0°
Absorption correction: multi-scan (SADABS; Bruker, 2014)	*h* = −14→15
*T*_min_ = 0.868, *T*_max_ = 0.931	*k* = −18→18
60005 measured reflections	*l* = −18→18

### Tris(5,5'-dimethyl-2,2'-bipyridine)iron(II) 1,1,3,3-tetracyano-2-propoxypropenide tetrafluoridoborate (VI) Refinement

**Table:**

Refinement on *F*^2^	30 restraints
Least-squares matrix: full	Hydrogen site location: inferred from neighbouring sites
*R*[*F*^2^ > 2σ(*F*^2^)] = 0.045	H-atom parameters constrained
*wR*(*F*^2^) = 0.111	*w* = 1/[σ^2^(*F*_o_^2^) + (0.0391*P*)^2^ + 2.3998*P*] where *P* = (*F*_o_^2^ + 2*F*_c_^2^)/3
*S* = 1.08	(Δ/σ)_max_ = 0.001
9584 reflections	Δρ_max_ = 0.68 e Å^−^^3^
712 parameters	Δρ_min_ = −0.37 e Å^−^^3^

### Tris(5,5'-dimethyl-2,2'-bipyridine)iron(II) 1,1,3,3-tetracyano-2-propoxypropenide tetrafluoridoborate (VI) Special details

**Table:**

Geometry. All esds (except the esd in the dihedral angle between two l.s. planes) are estimated using the full covariance matrix. The cell esds are taken into account individually in the estimation of esds in distances, angles and torsion angles; correlations between esds in cell parameters are only used when they are defined by crystal symmetry. An approximate (isotropic) treatment of cell esds is used for estimating esds involving l.s. planes.

### Tris(5,5'-dimethyl-2,2'-bipyridine)iron(II) 1,1,3,3-tetracyano-2-propoxypropenide tetrafluoridoborate (VI) Fractional atomic coordinates and isotropic or equivalent isotropic displacement parameters (Å^2^)

**Table:**

	*x*	*y*	*z*	*U*_iso_*/*U*_eq_	Occ. (<1)
Fe1	0.24146 (3)	0.17454 (2)	0.67013 (2)	0.01112 (8)	
N11	0.32652 (15)	0.29366 (13)	0.66720 (13)	0.0127 (3)	
C12	0.30515 (18)	0.38786 (16)	0.59637 (16)	0.0144 (4)	
C13	0.3589 (2)	0.47846 (17)	0.58509 (18)	0.0192 (5)	
H13	0.3435	0.5436	0.5348	0.023*	
C14	0.4351 (2)	0.47293 (17)	0.64792 (19)	0.0200 (5)	
H14	0.4722	0.5345	0.6408	0.024*	
C15	0.45740 (19)	0.37735 (17)	0.72129 (18)	0.0171 (4)	
C16	0.40122 (18)	0.28975 (16)	0.72716 (17)	0.0148 (4)	
H16	0.4165	0.2238	0.7761	0.018*	
C17	0.5376 (2)	0.36626 (19)	0.7924 (2)	0.0246 (5)	
H17A	0.5586	0.2942	0.8231	0.037*	
H17B	0.4966	0.3858	0.8494	0.037*	
H17C	0.6099	0.4115	0.7513	0.037*	
N21	0.17782 (15)	0.28698 (13)	0.56251 (13)	0.0130 (3)	
C22	0.22070 (18)	0.38414 (16)	0.53661 (17)	0.0144 (4)	
C23	0.1823 (2)	0.47079 (17)	0.46233 (18)	0.0199 (5)	
H23	0.2156	0.5380	0.4431	0.024*	
C24	0.0951 (2)	0.45799 (18)	0.41681 (19)	0.0232 (5)	
H24	0.0682	0.5165	0.3660	0.028*	
C25	0.0471 (2)	0.35859 (17)	0.44602 (18)	0.0190 (5)	
C26	0.09314 (19)	0.27640 (17)	0.51761 (17)	0.0159 (4)	
H26	0.0630	0.2083	0.5361	0.019*	
C27	−0.0523 (2)	0.33819 (19)	0.4051 (2)	0.0273 (5)	
H27A	−0.0929	0.2704	0.4540	0.041*	
H27B	−0.0198	0.3388	0.3350	0.041*	
H27C	−0.1089	0.3921	0.3996	0.041*	
N31	0.36701 (15)	0.16262 (13)	0.55529 (13)	0.0125 (3)	
C32	0.33629 (18)	0.09799 (16)	0.51615 (16)	0.0135 (4)	
C33	0.41024 (19)	0.08850 (17)	0.42852 (17)	0.0175 (4)	
H33	0.3866	0.0433	0.4020	0.021*	
C34	0.5184 (2)	0.14548 (17)	0.38053 (17)	0.0179 (4)	
H34	0.5690	0.1405	0.3201	0.021*	
C35	0.55254 (19)	0.21009 (16)	0.42137 (17)	0.0161 (4)	
C36	0.47370 (19)	0.21541 (16)	0.50880 (16)	0.0143 (4)	
H36	0.4966	0.2590	0.5374	0.017*	
C37	0.6697 (2)	0.27300 (18)	0.37358 (18)	0.0207 (5)	
H37A	0.6918	0.2899	0.4269	0.031*	
H37B	0.6625	0.3371	0.3130	0.031*	
H37C	0.7310	0.2329	0.3498	0.031*	
N41	0.16178 (15)	0.06476 (13)	0.65441 (13)	0.0128 (3)	
C42	0.22084 (19)	0.04074 (16)	0.57398 (16)	0.0143 (4)	
C43	0.1719 (2)	−0.03130 (17)	0.54922 (18)	0.0197 (5)	
H43	0.2145	−0.0475	0.4929	0.024*	
C44	0.0611 (2)	−0.07844 (18)	0.6076 (2)	0.0227 (5)	
H44	0.0269	−0.1273	0.5913	0.027*	
C45	−0.0010 (2)	−0.05504 (17)	0.69040 (18)	0.0189 (5)	
C46	0.05368 (19)	0.01673 (16)	0.71034 (17)	0.0155 (4)	
H46	0.0125	0.0332	0.7670	0.019*	
C47	−0.1230 (2)	−0.10338 (19)	0.7548 (2)	0.0265 (5)	
H47A	−0.1662	−0.0566	0.7831	0.040*	
H47B	−0.1162	−0.1692	0.8135	0.040*	
H47C	−0.1662	−0.1159	0.7097	0.040*	
N51	0.30211 (15)	0.07388 (13)	0.78841 (13)	0.0126 (3)	
C52	0.24036 (19)	0.06351 (17)	0.88668 (17)	0.0163 (4)	
C53	0.2798 (2)	0.00377 (18)	0.97607 (18)	0.0217 (5)	
H53	0.2374	−0.0007	1.0435	0.026*	
C54	0.3808 (2)	−0.04917 (18)	0.96694 (18)	0.0208 (5)	
H54	0.4082	−0.0903	1.0281	0.025*	
C55	0.4428 (2)	−0.04228 (16)	0.86788 (18)	0.0171 (4)	
C56	0.39989 (19)	0.02144 (16)	0.78094 (17)	0.0150 (4)	
H56	0.4422	0.0281	0.7126	0.018*	
C57	0.5510 (2)	−0.10075 (19)	0.8538 (2)	0.0263 (5)	
H57A	0.6061	−0.0599	0.7856	0.039*	
H57B	0.5277	−0.1669	0.8556	0.039*	
H57C	0.5899	−0.1137	0.9112	0.039*	
N61	0.11273 (16)	0.17489 (14)	0.78865 (14)	0.0144 (4)	
C62	0.13200 (19)	0.12057 (17)	0.88629 (17)	0.0175 (4)	
C63	0.0518 (2)	0.1174 (2)	0.97679 (19)	0.0273 (5)	
H63	0.0676	0.0797	1.0444	0.033*	
C64	−0.0512 (2)	0.1692 (2)	0.9684 (2)	0.0284 (6)	
H64	−0.1062	0.1678	1.0300	0.034*	
C65	−0.0737 (2)	0.22307 (19)	0.86942 (19)	0.0219 (5)	
C66	0.01175 (19)	0.22401 (17)	0.78174 (18)	0.0163 (4)	
H66	−0.0023	0.2614	0.7134	0.020*	
C67	−0.1825 (2)	0.2824 (2)	0.8526 (2)	0.0303 (6)	
H67A	−0.2120	0.2722	0.7987	0.045*	
H67B	−0.1623	0.3563	0.8288	0.045*	
H67C	−0.2441	0.2574	0.9193	0.045*	
C71	−0.2028 (15)	0.5851 (8)	1.0977 (13)	0.019 (4)	0.508 (6)
C72	−0.2427 (8)	0.5059 (5)	1.0779 (7)	0.015 (2)	0.508 (6)
C73	−0.1981 (9)	0.4082 (5)	1.0990 (12)	0.0202 (19)	0.508 (6)
C711	−0.2447 (18)	0.6860 (7)	1.0537 (13)	0.022 (5)	0.508 (6)
N711	−0.283 (4)	0.7653 (16)	1.021 (4)	0.032 (6)	0.508 (6)
C712	−0.1168 (11)	0.5731 (10)	1.1575 (9)	0.019 (3)	0.508 (6)
N712	−0.0540 (8)	0.5657 (10)	1.2100 (7)	0.045 (3)	0.508 (6)
O721	−0.3412 (5)	0.5281 (4)	1.0390 (4)	0.0236 (11)	0.508 (6)
C721	−0.3359 (5)	0.5228 (4)	0.9383 (3)	0.0235 (12)	0.508 (6)
H71A	−0.4008	0.4746	0.9472	0.028*	0.508 (6)
H71B	−0.2592	0.4969	0.9142	0.028*	0.508 (6)
C722	−0.3483 (6)	0.6295 (4)	0.8576 (4)	0.0280 (13)	0.508 (6)
H72A	−0.2826	0.6768	0.8490	0.034*	0.508 (6)
H72B	−0.4240	0.6555	0.8837	0.034*	0.508 (6)
C723	−0.3462 (6)	0.6307 (4)	0.7514 (4)	0.0407 (16)	0.508 (6)
H73A	−0.3494	0.7020	0.7002	0.061*	0.508 (6)
H73B	−0.4149	0.5885	0.7584	0.061*	0.508 (6)
H73C	−0.2729	0.6022	0.7267	0.061*	0.508 (6)
C731	−0.2685 (7)	0.3264 (4)	1.1040 (6)	0.0221 (15)	0.508 (6)
N731	−0.3251 (6)	0.2590 (4)	1.1109 (5)	0.0327 (14)	0.508 (6)
C732	−0.0846 (11)	0.3844 (8)	1.1195 (13)	0.022 (2)	0.508 (6)
N732	0.0050 (7)	0.3569 (7)	1.1383 (7)	0.0340 (17)	0.508 (6)
C81	−0.1928 (15)	0.5784 (7)	1.0992 (13)	0.018 (4)	0.492 (6)
C82	−0.2136 (9)	0.4983 (6)	1.0721 (8)	0.017 (2)	0.492 (6)
C83	−0.1612 (9)	0.4031 (5)	1.1011 (12)	0.018 (2)	0.492 (6)
C811	−0.2387 (19)	0.6768 (9)	1.0509 (17)	0.019 (4)	0.492 (6)
N811	−0.268 (3)	0.7593 (14)	1.014 (4)	0.025 (3)	0.492 (6)
C812	−0.1371 (12)	0.5648 (10)	1.1821 (9)	0.016 (2)	0.492 (6)
N812	−0.0887 (7)	0.5584 (9)	1.2447 (6)	0.0260 (18)	0.492 (6)
O821	−0.2983 (5)	0.5176 (4)	1.0167 (4)	0.0254 (12)	0.492 (6)
C821	−0.2769 (6)	0.4898 (4)	0.9267 (4)	0.0294 (14)	0.492 (6)
H81A	−0.3325	0.4311	0.9431	0.035*	0.492 (6)
H81B	−0.1947	0.4685	0.9121	0.035*	0.492 (6)
C822	−0.2952 (5)	0.5825 (4)	0.8318 (4)	0.0334 (15)	0.492 (6)
H82A	−0.2802	0.5647	0.7703	0.040*	0.492 (6)
H82B	−0.2368	0.6394	0.8148	0.040*	0.492 (6)
C823	−0.4186 (6)	0.6195 (5)	0.8489 (5)	0.0427 (18)	0.492 (6)
H83A	−0.4276	0.6765	0.7830	0.064*	0.492 (6)
H83B	−0.4313	0.6438	0.9051	0.064*	0.492 (6)
H83C	−0.4771	0.5624	0.8695	0.064*	0.492 (6)
C831	−0.2227 (7)	0.3133 (4)	1.1131 (7)	0.0235 (16)	0.492 (6)
N831	−0.2727 (6)	0.2391 (4)	1.1274 (5)	0.0328 (15)	0.492 (6)
C832	−0.0511 (10)	0.3892 (8)	1.1298 (13)	0.024 (2)	0.492 (6)
N832	0.0401 (6)	0.3768 (6)	1.1496 (7)	0.0312 (16)	0.492 (6)
B91	0.2839 (3)	−0.1756 (2)	0.3311 (2)	0.0236 (6)	
F91	0.32293 (13)	−0.11607 (11)	0.37559 (11)	0.0283 (3)	
F92	0.16495 (15)	−0.16515 (14)	0.33588 (15)	0.0483 (5)	
F93	0.34885 (17)	−0.14255 (12)	0.22672 (12)	0.0419 (4)	
F94	0.30316 (14)	−0.27935 (11)	0.38832 (12)	0.0334 (4)	

### Tris(5,5'-dimethyl-2,2'-bipyridine)iron(II) 1,1,3,3-tetracyano-2-propoxypropenide tetrafluoridoborate (VI) Atomic displacement parameters (Å^2^)

**Table:**

	*U*^11^	*U*^22^	*U*^33^	*U*^12^	*U*^13^	*U*^23^
Fe1	0.01230 (16)	0.01144 (15)	0.01054 (15)	0.00138 (11)	−0.00290 (11)	−0.00551 (11)
N11	0.0119 (9)	0.0146 (8)	0.0126 (8)	0.0009 (7)	−0.0016 (7)	−0.0072 (7)
C12	0.0134 (10)	0.0152 (10)	0.0151 (10)	0.0030 (8)	−0.0023 (8)	−0.0076 (8)
C13	0.0211 (12)	0.0146 (10)	0.0233 (12)	0.0030 (9)	−0.0075 (9)	−0.0084 (9)
C14	0.0186 (11)	0.0166 (11)	0.0282 (12)	−0.0008 (9)	−0.0056 (9)	−0.0127 (9)
C15	0.0136 (10)	0.0202 (11)	0.0211 (11)	0.0009 (8)	−0.0035 (9)	−0.0124 (9)
C16	0.0141 (10)	0.0167 (10)	0.0143 (10)	0.0013 (8)	−0.0033 (8)	−0.0073 (8)
C17	0.0240 (12)	0.0253 (12)	0.0321 (13)	0.0007 (10)	−0.0139 (10)	−0.0155 (11)
N21	0.0129 (9)	0.0137 (8)	0.0131 (8)	0.0013 (7)	−0.0016 (7)	−0.0072 (7)
C22	0.0139 (10)	0.0152 (10)	0.0153 (10)	0.0020 (8)	−0.0030 (8)	−0.0078 (8)
C23	0.0247 (12)	0.0145 (10)	0.0225 (11)	0.0033 (9)	−0.0097 (10)	−0.0079 (9)
C24	0.0312 (13)	0.0181 (11)	0.0235 (12)	0.0072 (10)	−0.0142 (10)	−0.0083 (9)
C25	0.0208 (12)	0.0218 (11)	0.0208 (11)	0.0076 (9)	−0.0110 (9)	−0.0125 (9)
C26	0.0158 (11)	0.0161 (10)	0.0182 (11)	0.0032 (8)	−0.0056 (9)	−0.0091 (9)
C27	0.0325 (14)	0.0260 (13)	0.0321 (14)	0.0081 (10)	−0.0223 (11)	−0.0133 (11)
N31	0.0132 (9)	0.0129 (8)	0.0119 (8)	0.0029 (7)	−0.0046 (7)	−0.0050 (7)
C32	0.0143 (10)	0.0135 (10)	0.0131 (10)	0.0033 (8)	−0.0046 (8)	−0.0056 (8)
C33	0.0182 (11)	0.0200 (11)	0.0175 (11)	0.0029 (9)	−0.0050 (9)	−0.0109 (9)
C34	0.0184 (11)	0.0215 (11)	0.0135 (10)	0.0036 (9)	−0.0021 (9)	−0.0084 (9)
C35	0.0142 (10)	0.0169 (10)	0.0134 (10)	0.0026 (8)	−0.0039 (8)	−0.0026 (8)
C36	0.0153 (10)	0.0138 (10)	0.0143 (10)	0.0019 (8)	−0.0050 (8)	−0.0057 (8)
C37	0.0178 (11)	0.0234 (11)	0.0192 (11)	−0.0012 (9)	−0.0004 (9)	−0.0094 (9)
N41	0.0135 (9)	0.0118 (8)	0.0125 (8)	0.0028 (7)	−0.0041 (7)	−0.0040 (7)
C42	0.0146 (10)	0.0126 (10)	0.0151 (10)	0.0021 (8)	−0.0034 (8)	−0.0056 (8)
C43	0.0202 (11)	0.0204 (11)	0.0233 (12)	0.0021 (9)	−0.0036 (9)	−0.0147 (9)
C44	0.0203 (12)	0.0204 (11)	0.0325 (13)	−0.0012 (9)	−0.0055 (10)	−0.0162 (10)
C45	0.0184 (11)	0.0147 (10)	0.0222 (11)	0.0000 (8)	−0.0039 (9)	−0.0068 (9)
C46	0.0160 (11)	0.0154 (10)	0.0144 (10)	0.0026 (8)	−0.0035 (8)	−0.0057 (8)
C47	0.0213 (12)	0.0247 (12)	0.0328 (14)	−0.0063 (10)	−0.0008 (10)	−0.0139 (11)
N51	0.0132 (9)	0.0119 (8)	0.0130 (8)	−0.0009 (7)	−0.0033 (7)	−0.0054 (7)
C52	0.0142 (10)	0.0203 (11)	0.0132 (10)	−0.0022 (8)	−0.0018 (8)	−0.0063 (9)
C53	0.0203 (12)	0.0289 (12)	0.0126 (10)	−0.0027 (9)	−0.0047 (9)	−0.0047 (9)
C54	0.0218 (12)	0.0203 (11)	0.0168 (11)	−0.0033 (9)	−0.0101 (9)	−0.0012 (9)
C55	0.0191 (11)	0.0110 (10)	0.0220 (11)	−0.0015 (8)	−0.0097 (9)	−0.0050 (8)
C56	0.0181 (11)	0.0128 (10)	0.0149 (10)	0.0001 (8)	−0.0052 (8)	−0.0059 (8)
C57	0.0318 (14)	0.0218 (12)	0.0282 (13)	0.0113 (10)	−0.0169 (11)	−0.0090 (10)
N61	0.0141 (9)	0.0169 (9)	0.0148 (9)	−0.0001 (7)	−0.0041 (7)	−0.0088 (7)
C62	0.0145 (11)	0.0236 (11)	0.0147 (10)	−0.0009 (8)	−0.0035 (8)	−0.0083 (9)
C63	0.0205 (12)	0.0459 (16)	0.0143 (11)	0.0021 (11)	−0.0031 (9)	−0.0121 (11)
C64	0.0184 (12)	0.0502 (16)	0.0203 (12)	0.0037 (11)	0.0002 (10)	−0.0211 (12)
C65	0.0144 (11)	0.0314 (13)	0.0246 (12)	0.0016 (9)	−0.0020 (9)	−0.0178 (10)
C66	0.0143 (10)	0.0185 (10)	0.0183 (11)	0.0009 (8)	−0.0035 (8)	−0.0100 (9)
C67	0.0204 (13)	0.0500 (16)	0.0297 (14)	0.0121 (11)	−0.0073 (10)	−0.0259 (13)
C71	0.024 (6)	0.028 (8)	0.015 (6)	0.001 (5)	−0.008 (4)	−0.016 (5)
C72	0.012 (4)	0.013 (3)	0.011 (3)	0.003 (2)	0.003 (3)	0.000 (2)
C73	0.017 (5)	0.025 (4)	0.017 (3)	0.003 (2)	0.001 (4)	−0.010 (2)
C711	0.030 (8)	0.016 (7)	0.011 (6)	−0.007 (5)	−0.005 (5)	0.003 (5)
N711	0.030 (11)	0.032 (6)	0.037 (12)	0.013 (5)	−0.014 (9)	−0.014 (6)
C712	0.031 (6)	0.012 (3)	0.008 (5)	−0.006 (3)	−0.005 (5)	0.004 (3)
N712	0.068 (6)	0.017 (3)	0.047 (5)	−0.009 (5)	−0.039 (5)	0.003 (5)
O721	0.022 (3)	0.031 (2)	0.028 (2)	0.007 (2)	−0.010 (2)	−0.0201 (18)
C721	0.028 (3)	0.022 (3)	0.026 (3)	0.005 (2)	−0.009 (2)	−0.014 (2)
C722	0.028 (3)	0.026 (3)	0.035 (3)	0.001 (2)	−0.011 (2)	−0.015 (2)
C723	0.059 (4)	0.034 (3)	0.030 (3)	0.013 (3)	−0.019 (3)	−0.011 (2)
C731	0.025 (4)	0.020 (3)	0.019 (3)	0.005 (3)	−0.002 (3)	−0.009 (2)
N731	0.042 (4)	0.024 (3)	0.028 (3)	−0.005 (3)	−0.002 (3)	−0.010 (2)
C732	0.028 (6)	0.020 (3)	0.016 (4)	0.006 (3)	−0.006 (4)	−0.006 (2)
N732	0.028 (5)	0.044 (4)	0.032 (3)	0.009 (3)	−0.007 (3)	−0.019 (3)
C81	0.016 (5)	0.006 (5)	0.018 (6)	0.003 (4)	−0.001 (4)	0.005 (4)
C82	0.011 (4)	0.028 (4)	0.019 (4)	0.004 (2)	−0.003 (3)	−0.017 (3)
C83	0.017 (6)	0.012 (3)	0.022 (3)	0.003 (3)	0.002 (5)	−0.009 (2)
C811	0.013 (6)	0.027 (8)	0.028 (7)	0.008 (4)	−0.009 (4)	−0.022 (7)
N811	0.020 (7)	0.023 (7)	0.024 (5)	0.003 (4)	−0.004 (5)	−0.004 (7)
C812	0.018 (4)	0.010 (4)	0.011 (5)	−0.007 (3)	0.006 (4)	−0.001 (4)
N812	0.041 (4)	0.016 (3)	0.023 (4)	−0.001 (3)	−0.017 (3)	−0.004 (4)
O821	0.030 (3)	0.030 (2)	0.027 (2)	0.007 (2)	−0.012 (2)	−0.0202 (19)
C821	0.042 (4)	0.032 (3)	0.025 (3)	0.003 (3)	−0.012 (3)	−0.021 (2)
C822	0.042 (3)	0.036 (3)	0.027 (3)	−0.006 (3)	−0.004 (2)	−0.019 (3)
C823	0.065 (5)	0.033 (3)	0.036 (3)	0.015 (3)	−0.024 (3)	−0.015 (3)
C831	0.025 (4)	0.023 (3)	0.021 (3)	0.006 (3)	0.003 (3)	−0.014 (2)
N831	0.045 (4)	0.026 (3)	0.027 (3)	−0.004 (3)	0.003 (3)	−0.016 (2)
C832	0.026 (6)	0.028 (4)	0.013 (4)	0.012 (4)	0.000 (5)	−0.008 (3)
N832	0.024 (4)	0.040 (4)	0.028 (3)	0.011 (3)	−0.002 (3)	−0.016 (3)
B91	0.0294 (15)	0.0218 (13)	0.0196 (13)	0.0006 (11)	−0.0064 (11)	−0.0084 (11)
F91	0.0371 (8)	0.0286 (8)	0.0246 (7)	0.0015 (6)	−0.0088 (6)	−0.0157 (6)
F92	0.0295 (9)	0.0480 (10)	0.0621 (12)	0.0023 (8)	−0.0216 (8)	−0.0124 (9)
F93	0.0698 (12)	0.0335 (9)	0.0192 (8)	−0.0089 (8)	0.0003 (8)	−0.0128 (7)
F94	0.0493 (10)	0.0218 (7)	0.0272 (8)	0.0048 (7)	−0.0060 (7)	−0.0106 (6)

### Tris(5,5'-dimethyl-2,2'-bipyridine)iron(II) 1,1,3,3-tetracyano-2-propoxypropenide tetrafluoridoborate (VI) Geometric parameters (Å, º)

**Table:**

Fe1—N41	1.9671 (17)	C54—H54	0.9500
Fe1—N21	1.9692 (18)	C55—C56	1.393 (3)
Fe1—N51	1.9712 (18)	C55—C57	1.498 (3)
Fe1—N61	1.9752 (18)	C56—H56	0.9500
Fe1—N31	1.9794 (17)	C57—H57A	0.9800
Fe1—N11	1.9798 (17)	C57—H57B	0.9800
N11—C16	1.346 (3)	C57—H57C	0.9800
N11—C12	1.359 (3)	N61—C66	1.344 (3)
C12—C13	1.388 (3)	N61—C62	1.356 (3)
C12—C22	1.465 (3)	C62—C63	1.386 (3)
C13—C14	1.386 (3)	C63—C64	1.384 (4)
C13—H13	0.9500	C63—H63	0.9500
C14—C15	1.390 (3)	C64—C65	1.385 (3)
C14—H14	0.9500	C64—H64	0.9500
C15—C16	1.394 (3)	C65—C66	1.396 (3)
C15—C17	1.498 (3)	C65—C67	1.503 (3)
C16—H16	0.9500	C66—H66	0.9500
C17—H17A	0.9800	C67—H67A	0.9800
C17—H17B	0.9800	C67—H67B	0.9800
C17—H17C	0.9800	C67—H67C	0.9800
N21—C26	1.343 (3)	C71—C72	1.394 (5)
N21—C22	1.357 (3)	C71—C711	1.425 (7)
C22—C23	1.393 (3)	C71—C712	1.428 (7)
C23—C24	1.385 (3)	C72—O721	1.361 (5)
C23—H23	0.9500	C72—C73	1.400 (6)
C24—C25	1.397 (3)	C73—C732	1.417 (8)
C24—H24	0.9500	C73—C731	1.419 (6)
C25—C26	1.385 (3)	C711—N711	1.146 (7)
C25—C27	1.503 (3)	C712—N712	1.141 (5)
C26—H26	0.9500	O721—C721	1.461 (5)
C27—H27A	0.9800	C721—C722	1.504 (6)
C27—H27B	0.9800	C721—H71A	0.9900
C27—H27C	0.9800	C721—H71B	0.9900
N31—C36	1.345 (3)	C722—C723	1.508 (6)
N31—C32	1.358 (3)	C722—H72A	0.9900
C32—C33	1.394 (3)	C722—H72B	0.9900
C32—C42	1.464 (3)	C723—H73A	0.9800
C33—C34	1.384 (3)	C723—H73B	0.9800
C33—H33	0.9500	C723—H73C	0.9800
C34—C35	1.392 (3)	C731—N731	1.143 (5)
C34—H34	0.9500	C732—N732	1.150 (9)
C35—C36	1.390 (3)	C81—C82	1.396 (5)
C35—C37	1.506 (3)	C81—C811	1.425 (7)
C36—H36	0.9500	C81—C812	1.428 (7)
C37—H37A	0.9800	C82—O821	1.359 (5)
C37—H37B	0.9800	C82—C83	1.400 (6)
C37—H37C	0.9800	C83—C832	1.418 (8)
N41—C46	1.349 (3)	C83—C831	1.418 (6)
N41—C42	1.358 (3)	C811—N811	1.147 (7)
C42—C43	1.397 (3)	C812—N812	1.140 (5)
C43—C44	1.376 (3)	O821—C821	1.464 (5)
C43—H43	0.9500	C821—C822	1.501 (6)
C44—C45	1.391 (3)	C821—H81A	0.9900
C44—H44	0.9500	C821—H81B	0.9900
C45—C46	1.385 (3)	C822—C823	1.506 (6)
C45—C47	1.502 (3)	C822—H82A	0.9900
C46—H46	0.9500	C822—H82B	0.9900
C47—H47A	0.9800	C823—H83A	0.9800
C47—H47B	0.9800	C823—H83B	0.9800
C47—H47C	0.9800	C823—H83C	0.9800
N51—C56	1.341 (3)	C831—N831	1.144 (5)
N51—C52	1.366 (3)	C832—N832	1.150 (9)
C52—C53	1.384 (3)	B91—F92	1.372 (3)
C52—C62	1.470 (3)	B91—F93	1.387 (3)
C53—C54	1.377 (3)	B91—F91	1.398 (3)
C53—H53	0.9500	B91—F94	1.398 (3)
C54—C55	1.392 (3)		
			
N41—Fe1—N21	93.63 (7)	N51—C52—C62	113.80 (18)
N41—Fe1—N51	92.53 (7)	C53—C52—C62	125.1 (2)
N21—Fe1—N51	172.24 (7)	C54—C53—C52	119.8 (2)
N41—Fe1—N61	94.58 (7)	C54—C53—H53	120.1
N21—Fe1—N61	93.22 (7)	C52—C53—H53	120.1
N51—Fe1—N61	81.58 (7)	C53—C54—C55	119.9 (2)
N41—Fe1—N31	81.57 (7)	C53—C54—H54	120.1
N21—Fe1—N31	89.27 (7)	C55—C54—H54	120.1
N51—Fe1—N31	96.29 (7)	C54—C55—C56	117.3 (2)
N61—Fe1—N31	175.55 (7)	C54—C55—C57	122.1 (2)
N41—Fe1—N11	173.13 (7)	C56—C55—C57	120.6 (2)
N21—Fe1—N11	81.38 (7)	N51—C56—C55	123.6 (2)
N51—Fe1—N11	92.85 (7)	N51—C56—H56	118.2
N61—Fe1—N11	90.42 (7)	C55—C56—H56	118.2
N31—Fe1—N11	93.60 (7)	C55—C57—H57A	109.5
C16—N11—C12	118.45 (18)	C55—C57—H57B	109.5
C16—N11—Fe1	126.73 (14)	H57A—C57—H57B	109.5
C12—N11—Fe1	114.82 (14)	C55—C57—H57C	109.5
N11—C12—C13	121.4 (2)	H57A—C57—H57C	109.5
N11—C12—C22	114.34 (18)	H57B—C57—H57C	109.5
C13—C12—C22	124.30 (19)	C66—N61—C62	118.12 (18)
C14—C13—C12	119.4 (2)	C66—N61—Fe1	127.01 (15)
C14—C13—H13	120.3	C62—N61—Fe1	114.86 (14)
C12—C13—H13	120.3	N61—C62—C63	121.4 (2)
C13—C14—C15	120.1 (2)	N61—C62—C52	114.27 (18)
C13—C14—H14	120.0	C63—C62—C52	124.3 (2)
C15—C14—H14	120.0	C64—C63—C62	119.8 (2)
C14—C15—C16	117.2 (2)	C64—C63—H63	120.1
C14—C15—C17	122.7 (2)	C62—C63—H63	120.1
C16—C15—C17	120.1 (2)	C63—C64—C65	119.5 (2)
N11—C16—C15	123.5 (2)	C63—C64—H64	120.2
N11—C16—H16	118.2	C65—C64—H64	120.2
C15—C16—H16	118.2	C64—C65—C66	117.5 (2)
C15—C17—H17A	109.5	C64—C65—C67	123.2 (2)
C15—C17—H17B	109.5	C66—C65—C67	119.3 (2)
H17A—C17—H17B	109.5	N61—C66—C65	123.6 (2)
C15—C17—H17C	109.5	N61—C66—H66	118.2
H17A—C17—H17C	109.5	C65—C66—H66	118.2
H17B—C17—H17C	109.5	C65—C67—H67A	109.5
C26—N21—C22	118.11 (18)	C65—C67—H67B	109.5
C26—N21—Fe1	126.26 (14)	H67A—C67—H67B	109.5
C22—N21—Fe1	115.54 (14)	C65—C67—H67C	109.5
N21—C22—C23	121.46 (19)	H67A—C67—H67C	109.5
N21—C22—C12	113.88 (18)	H67B—C67—H67C	109.5
C23—C22—C12	124.62 (19)	C72—C71—C711	119.9 (6)
C24—C23—C22	119.4 (2)	C72—C71—C712	123.8 (6)
C24—C23—H23	120.3	C711—C71—C712	116.3 (5)
C22—C23—H23	120.3	O721—C72—C71	113.2 (5)
C23—C24—C25	119.6 (2)	O721—C72—C73	118.8 (5)
C23—C24—H24	120.2	C71—C72—C73	127.9 (6)
C25—C24—H24	120.2	C72—C73—C732	122.9 (5)
C26—C25—C24	117.3 (2)	C72—C73—C731	120.2 (7)
C26—C25—C27	119.6 (2)	C732—C73—C731	116.8 (5)
C24—C25—C27	123.1 (2)	N711—C711—C71	177 (3)
N21—C26—C25	124.1 (2)	N712—C712—C71	175.1 (16)
N21—C26—H26	118.0	C72—O721—C721	118.0 (5)
C25—C26—H26	118.0	O721—C721—C722	108.6 (4)
C25—C27—H27A	109.5	O721—C721—H71A	110.0
C25—C27—H27B	109.5	C722—C721—H71A	110.0
H27A—C27—H27B	109.5	O721—C721—H71B	110.0
C25—C27—H27C	109.5	C722—C721—H71B	110.0
H27A—C27—H27C	109.5	H71A—C721—H71B	108.3
H27B—C27—H27C	109.5	C721—C722—C723	112.1 (4)
C36—N31—C32	117.80 (18)	C721—C722—H72A	109.2
C36—N31—Fe1	127.47 (14)	C723—C722—H72A	109.2
C32—N31—Fe1	114.59 (14)	C721—C722—H72B	109.2
N31—C32—C33	121.71 (19)	C723—C722—H72B	109.2
N31—C32—C42	114.12 (18)	H72A—C722—H72B	107.9
C33—C32—C42	124.17 (19)	C722—C723—H73A	109.5
C34—C33—C32	119.4 (2)	C722—C723—H73B	109.5
C34—C33—H33	120.3	H73A—C723—H73B	109.5
C32—C33—H33	120.3	C722—C723—H73C	109.5
C33—C34—C35	119.6 (2)	H73A—C723—H73C	109.5
C33—C34—H34	120.2	H73B—C723—H73C	109.5
C35—C34—H34	120.2	N731—C731—C73	178.1 (10)
C36—C35—C34	117.5 (2)	N732—C732—C73	174.5 (9)
C36—C35—C37	120.37 (19)	C82—C81—C811	120.1 (6)
C34—C35—C37	122.1 (2)	C82—C81—C812	123.1 (6)
N31—C36—C35	123.97 (19)	C811—C81—C812	116.4 (6)
N31—C36—H36	118.0	O821—C82—C81	113.2 (5)
C35—C36—H36	118.0	O821—C82—C83	120.2 (5)
C35—C37—H37A	109.5	C81—C82—C83	126.5 (6)
C35—C37—H37B	109.5	C82—C83—C832	122.3 (5)
H37A—C37—H37B	109.5	C82—C83—C831	120.7 (7)
C35—C37—H37C	109.5	C832—C83—C831	116.8 (6)
H37A—C37—H37C	109.5	N811—C811—C81	175 (3)
H37B—C37—H37C	109.5	N812—C812—C81	176.4 (14)
C46—N41—C42	117.91 (18)	C82—O821—C821	117.8 (5)
C46—N41—Fe1	126.94 (14)	O821—C821—C822	108.3 (4)
C42—N41—Fe1	115.03 (14)	O821—C821—H81A	110.0
N41—C42—C43	121.48 (19)	C822—C821—H81A	110.0
N41—C42—C32	114.27 (18)	O821—C821—H81B	110.0
C43—C42—C32	124.24 (19)	C822—C821—H81B	110.0
C44—C43—C42	119.0 (2)	H81A—C821—H81B	108.4
C44—C43—H43	120.5	C821—C822—C823	112.9 (4)
C42—C43—H43	120.5	C821—C822—H82A	109.0
C43—C44—C45	120.5 (2)	C823—C822—H82A	109.0
C43—C44—H44	119.8	C821—C822—H82B	109.0
C45—C44—H44	119.8	C823—C822—H82B	109.0
C46—C45—C44	117.0 (2)	H82A—C822—H82B	107.8
C46—C45—C47	120.9 (2)	C822—C823—H83A	109.5
C44—C45—C47	122.1 (2)	C822—C823—H83B	109.5
N41—C46—C45	124.1 (2)	H83A—C823—H83B	109.5
N41—C46—H46	118.0	C822—C823—H83C	109.5
C45—C46—H46	118.0	H83A—C823—H83C	109.5
C45—C47—H47A	109.5	H83B—C823—H83C	109.5
C45—C47—H47B	109.5	N831—C831—C83	177.0 (10)
H47A—C47—H47B	109.5	N832—C832—C83	177.2 (14)
C45—C47—H47C	109.5	F92—B91—F93	110.6 (2)
H47A—C47—H47C	109.5	F92—B91—F91	109.7 (2)
H47B—C47—H47C	109.5	F93—B91—F91	108.8 (2)
C56—N51—C52	118.30 (18)	F92—B91—F94	109.7 (2)
C56—N51—Fe1	126.76 (14)	F93—B91—F94	109.4 (2)
C52—N51—Fe1	114.79 (14)	F91—B91—F94	108.7 (2)
N51—C52—C53	121.1 (2)		
			
C16—N11—C12—C13	0.2 (3)	Fe1—N41—C46—C45	175.28 (16)
Fe1—N11—C12—C13	−179.76 (16)	C44—C45—C46—N41	0.4 (3)
C16—N11—C12—C22	−178.70 (18)	C47—C45—C46—N41	−178.6 (2)
Fe1—N11—C12—C22	1.3 (2)	C56—N51—C52—C53	2.5 (3)
N11—C12—C13—C14	−0.5 (3)	Fe1—N51—C52—C53	−173.31 (17)
C22—C12—C13—C14	178.3 (2)	C56—N51—C52—C62	−177.43 (18)
C12—C13—C14—C15	0.1 (3)	Fe1—N51—C52—C62	6.7 (2)
C13—C14—C15—C16	0.6 (3)	N51—C52—C53—C54	−2.2 (3)
C13—C14—C15—C17	−179.2 (2)	C62—C52—C53—C54	177.7 (2)
C12—N11—C16—C15	0.5 (3)	C52—C53—C54—C55	0.1 (3)
Fe1—N11—C16—C15	−179.47 (16)	C53—C54—C55—C56	1.6 (3)
C14—C15—C16—N11	−1.0 (3)	C53—C54—C55—C57	−178.1 (2)
C17—C15—C16—N11	178.8 (2)	C52—N51—C56—C55	−0.7 (3)
C26—N21—C22—C23	−2.7 (3)	Fe1—N51—C56—C55	174.55 (15)
Fe1—N21—C22—C23	−179.39 (16)	C54—C55—C56—N51	−1.3 (3)
C26—N21—C22—C12	175.10 (18)	C57—C55—C56—N51	178.4 (2)
Fe1—N21—C22—C12	−1.6 (2)	C66—N61—C62—C63	−1.6 (3)
N11—C12—C22—N21	0.2 (3)	Fe1—N61—C62—C63	177.26 (18)
C13—C12—C22—N21	−178.7 (2)	C66—N61—C62—C52	175.88 (18)
N11—C12—C22—C23	177.9 (2)	Fe1—N61—C62—C52	−5.3 (2)
C13—C12—C22—C23	−1.0 (3)	N51—C52—C62—N61	−0.9 (3)
N21—C22—C23—C24	2.5 (3)	C53—C52—C62—N61	179.1 (2)
C12—C22—C23—C24	−175.0 (2)	N51—C52—C62—C63	176.4 (2)
C22—C23—C24—C25	0.0 (4)	C53—C52—C62—C63	−3.5 (4)
C23—C24—C25—C26	−2.2 (3)	N61—C62—C63—C64	1.0 (4)
C23—C24—C25—C27	176.6 (2)	C52—C62—C63—C64	−176.2 (2)
C22—N21—C26—C25	0.3 (3)	C62—C63—C64—C65	0.5 (4)
Fe1—N21—C26—C25	176.61 (16)	C63—C64—C65—C66	−1.4 (4)
C24—C25—C26—N21	2.2 (3)	C63—C64—C65—C67	−179.3 (2)
C27—C25—C26—N21	−176.7 (2)	C62—N61—C66—C65	0.7 (3)
C36—N31—C32—C33	−2.1 (3)	Fe1—N61—C66—C65	−177.97 (16)
Fe1—N31—C32—C33	173.86 (16)	C64—C65—C66—N61	0.7 (3)
C36—N31—C32—C42	177.76 (18)	C67—C65—C66—N61	178.8 (2)
Fe1—N31—C32—C42	−6.3 (2)	C711—C71—C72—O721	−15 (2)
N31—C32—C33—C34	0.7 (3)	C712—C71—C72—O721	167.1 (14)
C42—C32—C33—C34	−179.2 (2)	C711—C71—C72—C73	168.6 (16)
C32—C33—C34—C35	1.0 (3)	C712—C71—C72—C73	−9 (3)
C33—C34—C35—C36	−1.1 (3)	O721—C72—C73—C732	167.3 (11)
C33—C34—C35—C37	179.2 (2)	C71—C72—C73—C732	−17 (2)
C32—N31—C36—C35	2.0 (3)	O721—C72—C73—C731	−14.8 (18)
Fe1—N31—C36—C35	−173.37 (15)	C71—C72—C73—C731	161.0 (13)
C34—C35—C36—N31	−0.4 (3)	C71—C72—O721—C721	124.3 (10)
C37—C35—C36—N31	179.24 (19)	C73—C72—O721—C721	−59.3 (12)
C46—N41—C42—C43	0.0 (3)	C72—O721—C721—C722	−115.6 (6)
Fe1—N41—C42—C43	−176.16 (16)	O721—C721—C722—C723	−178.9 (5)
C46—N41—C42—C32	178.80 (18)	C811—C81—C82—O821	−13 (2)
Fe1—N41—C42—C32	2.6 (2)	C812—C81—C82—O821	160.3 (14)
N31—C32—C42—N41	2.4 (3)	C811—C81—C82—C83	170.5 (17)
C33—C32—C42—N41	−177.69 (19)	C812—C81—C82—C83	−16 (3)
N31—C32—C42—C43	−178.9 (2)	O821—C82—C83—C832	160.8 (11)
C33—C32—C42—C43	1.0 (3)	C81—C82—C83—C832	−23 (2)
N41—C42—C43—C44	0.3 (3)	O821—C82—C83—C831	−25.4 (19)
C32—C42—C43—C44	−178.3 (2)	C81—C82—C83—C831	150.7 (14)
C42—C43—C44—C45	−0.3 (4)	C81—C82—O821—C821	138.2 (11)
C43—C44—C45—C46	−0.1 (3)	C83—C82—O821—C821	−45.1 (14)
C43—C44—C45—C47	178.9 (2)	C82—O821—C821—C822	−128.5 (7)
C42—N41—C46—C45	−0.4 (3)	O821—C821—C822—C823	−59.3 (6)

### Tris(5,5'-dimethyl-2,2'-bipyridine)iron(II) 1,1,3,3-tetracyano-2-propoxypropenide tetrafluoridoborate (VI) Hydrogen-bond geometry (Å, º)

**Table:**

*D*—H···*A*	*D*—H	H···*A*	*D*···*A*	*D*—H···*A*
C43—H43···F91	0.95	2.37	3.308 (3)	170
C54—H54···F93^i^	0.95	2.54	3.316 (3)	139
C64—H64···N831	0.95	2.54	3.414 (7)	154

Symmetry code: (i) *x*, *y*, *z*+1.
